# Mitochondrial dysfunction precedes hippocampal IL-1β transcription and cognitive impairments after low-dose lipopolysaccharide injection in aged mice

**DOI:** 10.1016/j.heliyon.2024.e28974

**Published:** 2024-03-30

**Authors:** Yulim Lee, Xianshu Ju, Jianchen Cui, Tao Zhang, Boohwi Hong, Yoon Hee Kim, Youngkwon Ko, Jiho Park, Chul Hee Choi, Jun Young Heo, Woosuk Chung

**Affiliations:** a Department of Medical Science, Chungnam National University School of Medicine, Daejeon, South Korea; b Department of Biochemistry, Chungnam National University School of Medicine, Daejeon, South Korea; c Brain Korea 21 FOUR Project for Medical Science, Chungnam National University, Daejeon, South Korea; d Brain Research Institute, Chungnam National University School of Medicine, Daejeon, South Korea; e Department of Anesthesiology and Pain Medicine, Chungnam National University Hospital, Daejeon, South Korea; f Department of Anesthesiology, The First People's Hospital of Yunnan Province. The Affiliated Hospital of Kunming University of Science and Technology, Kunming, China; g Department of Anesthesiology and Pain Medicine, Chungnam National University School of Medicine, Daejeon, South Korea; h Department of Anesthesiology and Pain Medicine, Chungnam National University Hospital, Sejong, South Korea; i Department of Microbiology, Chungnam National University School of Medicine, Daejeon, South Korea; j Infection Control Convergence Research Center, Chungnam National University School of Medicine, Daejeon, South Korea

**Keywords:** Cognition, Inflammation, Lipopolysaccharide, Mitochondria, Neuroinflammation, Old age

## Abstract

Acute cognitive impairments termed delirium often occur after inflammatory insults in elderly patients. While previous preclinical studies suggest mitochondria as a target for reducing neuroinflammation and cognitive impairments after LPS injection, fewer studies have evaluated the effects of a low-grade systemic inflammation in the aged brain. Thus, to identify the significance of mitochondrial dysfunction after a clinically relevant systemic inflammatory stimulus, we injected old-aged mice (18–20 months) with low-dose lipopolysaccharide (LPS, 0.04 mg/kg). LPS injection reduced mitochondrial respiration in the hippocampus 24 h after injection (respiratory control ratio [RCR], state3u/state4o; control = 2.82 ± 0.19, LPS = 2.57 ± 0.08). However, gene expression of the pro-inflammatory cytokine IL-1β was increased (RT-PCR, control = 1.00 ± 0.30; LPS = 2.01 ± 0.67) at a more delayed time point, 48 h after LPS injection. Such changes were associated with cognitive impairments in the Barnes maze and fear chamber tests. Notably, young mice were unaffected by low-dose LPS, suggesting that mitochondrial dysfunction precedes neuroinflammation and cognitive decline in elderly patients following a low-grade systemic insult. Our findings highlight mitochondria as a potential therapeutic target for reducing delirium in elderly patients.

## 1 Introduction

Modern societies face enormous economic and social burdens owing to rapid increases in the aging population, as the elderly are more susceptible to stressful events due to progressive declines in tissue and organ function [1,2]. Acute cognitive deterioration, termed delirium, also increases in old-age patients after stressful stimuli, such as infection, surgery, and traumatic injuries [3,4]. Despite being uncommon under the age of 65 years, delirium occurred in one-fifth of all acute medical in-patients [3], and has been identified as the most common complication after surgery in old-aged patients [5]. Recent studies have shown that delirium is associated with increased hospital stays, risk of re-institutionalization, mortality, and elevated healthcare costs [[6], [7], [8], [9]]. This has led to increasing efforts to identify novel treatment targets [[10], [11], [12], [13]].

Although the pathophysiology behind delirium is multifactorial [14,15], studies strongly suggest neuroinflammation as a significant contributing factor [[16], [17], [18], [19]]. Systemic inflammation originating from peripheral organs can activate microglia and initiate neuroinflammation via various pathways, including afferent nerve inputs, interaction between circulating immune mediators and endothelial cells, and active transport of proinflammatory cytokines and cells through the BBB (blood brain barrier) [20]. While it may be possible to reduce delirium by using anti-inflammatory agents, anti-inflammatory treatment itself can also increase the incidence of infection [21]. Therefore, there is a need to identify potential therapeutic targets with fewer adverse complications.

One potential target is the mitochondria, as there is an intricate relationship between mitochondria and neuroinflammation [[22], [23], [24]]. Inflammasomes, including the widely studied NLRP3 (nucleotide-binding domain leucine-rich repeat and pyrin domain containing receptor protein 3) inflammasome, are deeply involved with neuroinflammation development [25]. Recognition of pathogen-associated molecular patterns (PAMPs) and damage-associated molecular patterns (DAMPs) induces a cascade of signaling (MyD88, IRAKs, and TRAF6) that activates the transcription factor NF-κB. This upregulates the expression of inflammasome components and pro-inflammatory genes. Importantly, mitochondrial dysfunction can further activate inflammasome assembly and induce the release of pro-inflammatory cytokines (IL-1β, IL-18) [22]. Although the contribution of mitochondrial dysfunction to inflammasome activation is complex, mitochondria damage and mtDNA release have been suggested as down-stream events of inflammasome activation [26]. However, most studies were not performed in aged animals. Even in the absence of an acute inflammatory event, active interaction between mitochondria and inflammasomes may already exist in the aged brain [[27], [28], [29]]. This underlying interaction may contribute to the development of neuroinflammation and cognitive decline in elderly patients, especially when exposed to an inflammatory insult.

To investigate the significance of age-related mitochondrial dysfunction after a clinically relevant, acute systemic inflammatory insult, we administered a single injection of low-dose LPS in young and old mice [30]. Although LPS cannot efficiently penetrate the BBB [31], intraperitoneal LPS injection may indirectly affect the brain [20]. For example, LPS-induced inflammation can disrupt the BBB and increasing vascular permeability [32,33], resulting in increased infiltration of inflammatory mediators including inflammatory cytokines that can impair oxidative phosphorylation and affect mitochondrial function [28]. However, despite being widely used to study systemic inflammation and subsequent neuroinflammatory response [[34], [35], [36]], most studies used high doses or repeated administration of LPS. While high doses of LPS can model sepsis, a life-threatening systemic inflammation accompanied by excessive inflammation and high mortality [37], it does not accurately reflect the less severe inflammation observed in many clinical cases. Thus, the neurological consequences of a clinically relevant systemic inflammation induced by a single, low-dose LPS injection has not yet been fully studied, especially in aged animals [38,39]. In the present study, we examined age-dependent changes in mitochondrial function and expression of inflammatory cytokines in the hippocampus. Changes in cognitive function were further evaluated by assessing learning and memory in the Barnes maze and fear-chamber test. We found that low-dose LPS induced sustained increases in cytokine gene expression and cognitive deficits only in old-age mice. Most importantly, we discovered that such changes occurred after the development of mitochondrial dysfunction.

## 2 Materials and methods

### 2.1 Experimental animals

All studies were performed in C57BL/6 mice as they exhibit consistent behavioral responses, minimizing experimental variability [40,41]. Two age groups were used: young (8–10 weeks) and old (18–20 months), both sourced from Damul Science in Daejeon, South Korea. Ethical approval for the study was granted by the Institutional Review Board (IRB) of 10.13039/501100002462Chungnam National University, Daejeon, Republic of Korea (202006A-CNU-115). All experimental procedures adhered strictly to pertinent guidelines and regulatory standards. Mice were accommodated in standardized cages and kept under controlled environmental conditions of 24 °C and subjected to a diurnal 12-h light/12-h dark regime. Food and water were provided ad libitum. Authors complied with the ARRIVE guidelines.

### 2.2 LPS and rotenone treatment

For LPS treatment, both young (aged 8–10 weeks) and old (aged 18–20 months) mice were categorized into two distinct groups, control and LPS group. Old-age mice in the LPS group received a single intraperitoneal (i.p.) injection of 0.04 mg/kg LPS (from Escherichia coli O111:B4, Sigma-Aldrich, St. Louis, MO, USA, CAT# L2630) [30]. Young mice received a higher dose of LPS (0.33 mg/kg), as previous studies showed that young mice are less sensitive to LPS [42]. Control mice received an identical volume of normal saline. To further investigate the significance of mitochondrial dysfunction in the development of neuroinflammation in young mice, mice were injected with 2.5 mg/kg rotenone (i.p.), a mitochondrial complex I inhibitor, for 3 consecutive days as previously described [43]. Rotenone was first mixed with dimethyl sulfoxide (DMSO) to a final concentration of 1%, and subsequently diluted using soybean oil (4 mg/ml) [44].

### 2.3 Behavioral tests

The effects of LPS injection on cognitive function were evaluated using Barnes maze and fear-chamber tests. All behavioral tests were performed during the light-on period in the same sequence for both the Control and LPS groups by an experimenter who was blinded to the conditions and were video-recorded for post-experimental analysis.

#### 2.3.1 Barnes maze test

The Barnes maze test (Scitech Korea Inc., Korea) was conducted 24 h after intraperitoneal injection of saline (Control group) or LPS (LPS group) as previously described [45]. In brief, the maze featured a circular platform measuring 92 cm in diameter and incorporated 20 holes, each with a diameter of 5 cm. Positioned 95 cm off the ground, the maze was encircled by spatial cues aligned at its height to aid mice in learning the location of an escape box situated beneath the platform. To encourage mice to seek this escape box, the maze was intensely illuminated to a brightness of 1400 lux at its center, and an 80 dB buzzer was activated during the trial. On the initial day, the mice underwent a 3-min acclimatization period inside the escape box before being returned to their respective cages. After this habituation phase, they were placed at the maze's center inside a black cylindrical ‘start chamber’, coinciding with the activation of the buzzer. Ten seconds later, the chamber was lifted, and the mice received gentle guidance to familiarize them with entering the escape box. Once the mice entered the escape box, the buzzer was turned off and mice remained in the escape box for 2 min (pre-training trial). The first training session was performed 1 h after the pre-training trial. Training sessions were conducted for 4 days (days 1–4), with 3 sessions daily and an inter-trial interval of 20 min. Trials ended when mice entered the escape box and the buzzer was turned off. Mice remained in the escape box for 1 min. When mice did not enter the escape box within 3 min, they were gently guided. Due to a 20-min interval between sessions for each mouse, only four mice could be evaluated simultaneously during trials on days 1–4. This resulted in a total time of 60 min to complete three trial sessions for four mice. To ensure consistency in testing time, the daily maximum was set at 12 mice, requiring 3 h in total. On day 5, the escape box was removed, and mice were allowed to explore the maze for 90 s (probe trial). The probe trial on day 5 took 25 min for four mice (75 min for 12 mice).

The maze was cleaned using a 70% ethanol solution between mice. Additionally, the maze was rotated to minimize the reliance on potential intra-maze cues. Occasionally, mice might identify the correct target hole but hesitate to enter the escape box. To minimize the impact of this potential inconsistency, we evaluated the duration (primary latency), path length (primary path length), and number of errors (primary errors) to the first encounter of the escape hole. An experimenter, blinded to the experimental conditions, manually measured latency, errors, and path length. In contrast, the distance was automatically assessed (EthoVision XT tracking software, Noldus Information Technology, Netherlands).

#### 2.3.2 Fear chamber test

The fear chamber test (Coulbourn Instruments, MA, USA) was conducted as previously described [46]. During the conditioning trial, mice received three trials of a 1-mA electric stimulus at 1-min intervals after a 5-min habituation period (conditioning trial). Twenty-four hours later, contextual-fear memory was assessed by quantifying freezing behavior in the same chamber for a duration of 5 min using the FreezeFrame software (Coulbourn Instruments). The chamber was cleaned using a 70% ethanol solution between mice. Conditioning trials on day 1 and contextual fear memory assessment on day 2 took 40 and 30 min per four mice, respectively (120 and 90 min for 12 mice).

### 2.4 Mitochondrial oxygen consumption rate (OCR)

Mitochondrial oxygen consumption rate (OCR) was measured in mitochondria isolated from the hippocampus as previously described [47]. The hippocampus was first homogenized in mitochondrial isolation buffer (70 mM sucrose, 210 mM mannitol, 5 mM HEPES, 1 mM EGTA, 0.5% [w/v] fatty acid-free BSA [bovine serum albumen], pH 7.2). The homogenate was centrifuged (600×*g*, 10 min, 4 °C). The supernatant was further centrifuged (12,000×*g*, 10 min, 4 °C), and the pellet was resuspended using mitochondrial isolation buffer. Equal amounts (120 μg), quantified using the Bradford assay (Bio-Rad, Hercules, CA, USA), were uniformly diluted in 50 μl mitochondrial assay solution. This solution consists of 70 mM sucrose, 220 mM mannitol, 10 mM KH2PO4, 5 mM MgCl2, 2 mM HEPES, 1 mM EGTA, 0.2% (w/v) fatty acid-free BSA, 10 mM succinate, and 2 μM rotenone, with a pH of 7.2. The prepared mixtures were then allocated to an XF-24 plate (Seahorse Bioscience, Billerica, MA, USA). Following centrifugation of the XF plate (2000×*g*, 20 min, 4 °C), 450 μl of mitochondrial assay buffer was added to each well. Subsequently, the plates were incubated at 37 °C for a duration of 8–10 min. Oxygen consumption measurements were obtained under five different, sequentially induced respiratory states (Seahorse XF-24 extracellular flux analyzer, Seahorse Bioscience): i) basal respiration; ii) State3, with addition of ADP (final concentration, 4 mM) to measure oxygen consumption during ATP production (coupled respiration); iii) state4o, with addition of oligomycin (final concentration, 2.5 μg/ml) to measure oxygen consumption from protein leakage; iv) state3u, with addition of carbonyl cyanide *m*-chlorophenyl hydrazine (CCCP; final concentration, 4 μM) to measure oxygen consumption during maximal respiration (uncoupled respiration); and v) with addition of antimycin (final concentration, 4 μM) to measure non-mitochondrial respiration. Seahorse XF-24 software (Seahorse Bioscience) was used to automatically calculate and record OCR. In addition to measuring absolute OCR values, we also obtained the respiratory control ratios (RCRs), state3/state4o and state3u/state4o, which have been suggested as useful indicators of mitochondrial function in isolated mitochondria [48,49].

### 2.5 Real-time polymerase chain reaction (PCR)

Total RNA from the hippocampus was extracted 24 or 48 h after LPS injection. For ethical purposes, mice received 3% sevoflurane for 2 min before extracting the brain. cDNA was synthesized from total RNA using 5X reverse transcription (RT) premix. mRNA levels of hippocampal target genes were quantified with a Rotor Gene 6000 system (Corbett Life Science, Sydney, Australia) using 200 ng cDNA, 2X SYBR mix (PhileKorea, Seoul, Republic of Korea) and 10 pmol each forward/reverse primer. The indicated genes were amplified using the following primer pairs [50]: 18s rRNA, 5′-CTG GTT GAT CCT GCC AGT AG-3’ (forward) and 5′-CGA CCA AAG GAA CCA TAA CT-3’ (reverse); TNF-α, 5′-CCC ACG TCG TAG CAA ACC AC-3’ (forward) and 5′-GCA GCC TTG TCC CTT GAA GA-3’ (reverse); IL-1β, 5′-TGA CGG ACC CCA AAA GAT GA-3’ (forward) and 5′-AAA GAC ACA GGT AGC TGC CA-3’ (reverse); IL-10, 5′-ATA ACT GCA CCC ACT TCC CA-3’ (forward) and 5′-GGG CAT CAC TTC TAC CAG GT-3’ (reverse); and IL-6, 5′-ACA ACC ACG GCC TTC CCT ACT T-3’ (forward) and 5′-CAC GAT TTC CCA GAG AAC ATG TG-3’ (reverse).

### 2.6 Western blotting

The hippocampus was homogenized in RIPA lysis buffer (100 mM Tris-HCl pH 8.5, 200 mM NaCl, 5 mM EDTA, 0.2% SDS) after adding a phosphatase and protease inhibitor cocktail. The homogenate was microcentrifuged (16,000 rpm, 20 min, 4 °C), and the total protein concentrations of the supernatants were measured (Bradford assay, Bio-Rad, USA). After heating the samples at 100 °C for 7 min, proteins (10 μg each) were subjected to separation through SDS-polyacrylamide gel electrophoresis (PAGE) using 7.5–12% gels. Following the separation, the proteins were transferred to polyvinylidene fluoride (PVDF) membranes at a voltage of 70V for a duration of 2 h. After blocking for 30 min with Tris-buffered saline containing Tween 20 (TBST; 10 mM Tris-hydrochloride pH 7.6, 150 mM NaCl, 0.1% Tween 20) containing 5% skim milk, membranes were incubated at 4 °C overnight with primary antibodies. Membranes were then washed in TBST for 30 min and incubated with species-appropriate horseradish peroxidase (HRP)-conjugated anti-IgG secondary antibodies (Pierce Biotechnology, MA, USA) for 1 h at room temperature. Protein bands were detected with an enhanced chemiluminescence (ECL) system (WEST-ZOL plus, iNtRON BioTechnology, Seoul, Korea) using the Sensi-Q2000 Chemidoc system (Lugen Sci, Buchen, Republic of Korea). Primary antibodies against the following were used: OXPHOS (Abcam, Cambridge, United Kingdom, CAT#ab110413), OPA1 (BD Biosciences, Bedford, USA, CAT# 612606), DRP1 (Santa Cruz Biotechnology, California, USA, CAT# sc-32898), Mfn1 (Santa Cruz Biotechnology, CAT#sc-166644), Mfn2 (Santa Cruz Biotechnology, CAT# sc-100560), NLRP3 (AdipoGen Life Sciences, CAT# AG-20B-0014-C100), IL-1β (Cell signaling Technology, CAT#12507), Cleaved Caspase-1 (Cell signaling Technology, CAT#89332) and β-actin (Santa Cruz Biotechnology, CAT# sc-8432).

### 2.7 Statistical analysis

We utilized the R statistical software (version 4.2.0 from R Core Team, Austria) for data analysis. All continuous variables underwent tests for both normality and variance homogeneity. ANOVA (one-way analysis of variance) was applied only when these two conditions were satisfied. In cases where normality was not confirmed, the Kruskal–Wallis test was used. Welch's ANOVA was applied when there was a lack of variance homogeneity. For the analysis of repeatedly measured data, between-group, within-group, and group-time interaction effects were assessed using the “nparcomp” package, a nonparametric rank-based method [51]. P-values below 0.05 were deemed significant. Comprehensive statistical findings can be found in the Supplementary Data. All figures are presented as mean values ± standard deviation (SD).

## 3 Results

### 3.1 Hippocampal mitochondrial function is decreased in old-age mice

**Fig. 1:**
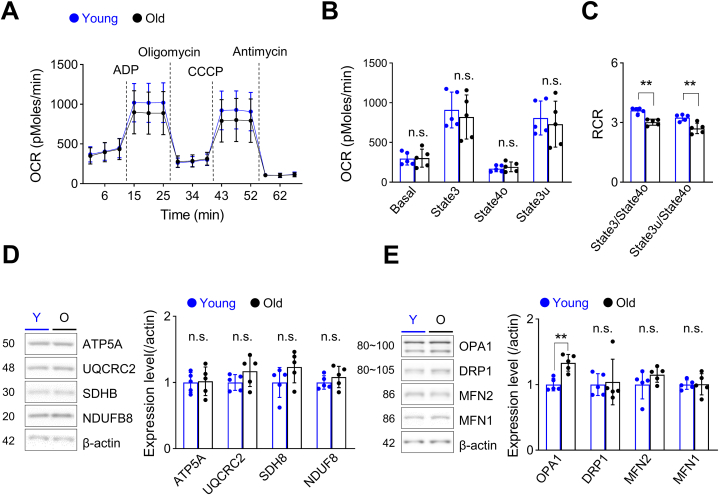
Mitochondrial function is decreased in mitochondria isolated from the hippocampus of old-age mice compared with that in young mice. **(A**–**C)** Mitochondrial function was evaluated by measuring respiration in mitochondria isolated from the hippocampus of young (n = 5) and old-age (n = 5) mice. **(A)** Mitochondrial OCR was measured while sequentially adding ADP, oligomycin, CCCP, and antimycin; dotted lines indicate the addition of compounds. **(B)** Quantification of OCR after excluding non-mitochondrial respiration. OCRs at all states were comparable between young and old mice (Student's t-test). **(C)** RCRs were significantly decreased in old-age mice (state3/state4o, p = 0.009, Kruskal-Wallis test; state3u/state4o, p = 0.004, Student's t-test). **(D)** (Left) Western blot analysis of mitochondrial complex subunits in hippocampal samples showing representative Western blot images. The number in the left-hand image indicates protein molecular weight (kDa). (Right) Summary data showing quantification of protein levels, presented as means ± SD. Expression levels of complex subunits was comparable between young (n = 5) and old (n = 5) mice (Student's t-test, Kruskal-Wallis test). **(E)** Western blot analysis of mitochondrial dynamic regulatory proteins in hippocampal samples from young (n = 5) and old (n = 5) mice showing representative Western blot images (left) and summary data (right). The number in the left-hand image indicates protein molecular weight (kDa). Only the expression level of OPA1 was significantly increased in old-age mice (p = 0.002, Student's t-test). Full Western blot images are provided in Supplementary Fig. 1. Values are presented as means ± SD (*p < 0.05, **p < 0.01, n.s., not significant).

Although aging is often linked to mitochondrial dysfunction, recent studies have reported relatively normal mitochondrial function in multiple organs (heart, liver, kidney) of old-age mice [52]. Previous studies that evaluated mitochondrial function have also reported inconsistent results [38,39,53]. Thus, we first investigated the effects of aging on mitochondrial function by measuring respiration in mitochondria isolated from the hippocampus. Although mitochondrial oxygen consumption rates (OCRs) were comparable at all states between young and old mice (Fig. 1A and B), respiratory control ratios (RCRs), which may represent the general function of isolated mitochondria [48,49], showed a significant decrease in old-aged mice (Fig. 1C). However, expression of OXPHOS complex subunits I (NDUFB8), II (SDHB), III (UQCRC2), and the ATP synthase subunit ATF5A was comparable between young and old mice (Fig. 1D, Suppl. Fig. 1). Mitochondria continuously undergo fusion and fission, and appropriate dynamic regulation is essential for maintaining mitochondrial function. Previous studies have shown that abnormalities in mitochondrial dynamics are also involved in neurodegenerative diseases and aging [[54], [55], [56]]. Thus, we next compared the protein levels of regulators of mitochondrial dynamics in young and old mice. Although we found no differences in the expression of the mitochondrial outer membrane fusion regulators mitofusion-1 (MFN1) and MFN2, or the mitochondrial fission regulator dynamin-related protein 1 (DRP1), we discovered a significant increase in OPA1 (optic atrophy 1), an inner mitochondrial membrane fusion regulator (Fig. 1E, Suppl. Fig. 1). Collectively, our results suggest reduced hippocampal mitochondrial function and altered expression of the mitochondrial dynamic protein OPA1 in the aged mouse hippocampus.

### 3.2 Intraperitoneal injection of low-dose LPS reduces mitochondria respiration in the hippocampus of old-age mice

**Fig. 2:**
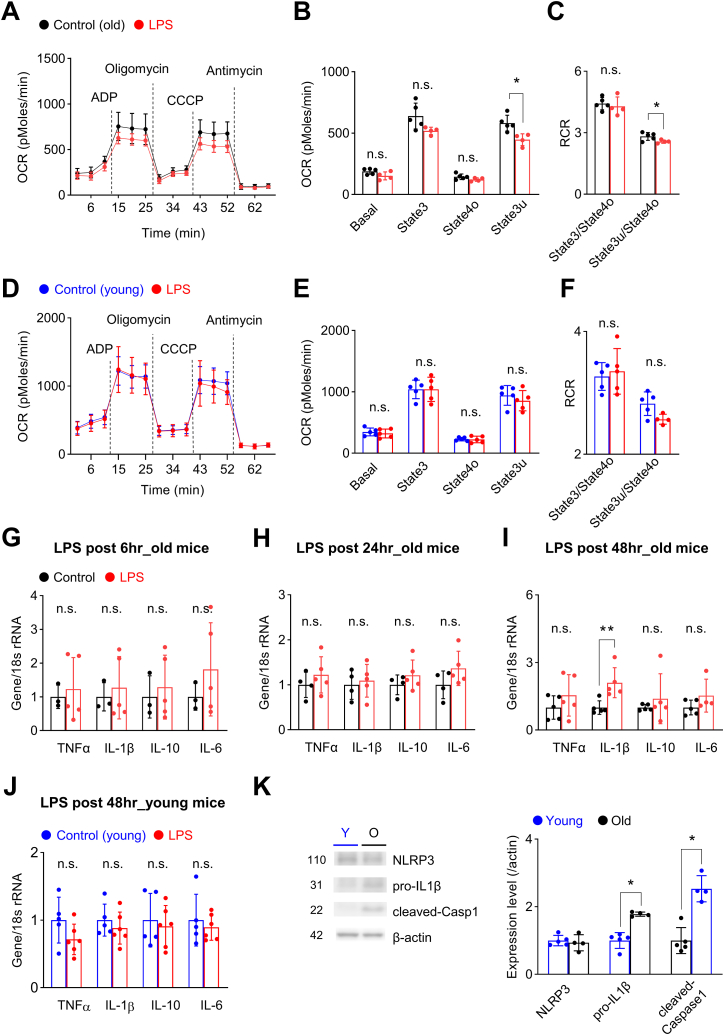
Mitochondrial dysfunction induced by low-dose LPS injection precedes increased gene transcription of IL-1β in old-age mice. **(A**–**C)** Mitochondrial respiration was measured 24 h after injection of 0.04 mg/kg LPS in old-age mice (Control, n = 5; LPS, n = 4). **(A)** Mitochondrial OCR was measured while sequentially adding ADP, oligomycin, CCCP, and antimycin; dotted lines indicate the addition of compounds. **(B)** Quantification of OCR after excluding non-mitochondrial respiration. Uncoupled respiration was significantly decreased after LPS injection (p = 0.011, Student's t-test). **(C)** RCR was significantly decreased in old-age mice (state3u/state4o, p = 0.044, Student's t-test; young). **(D**–**F)** Mitochondrial function was evaluated by measuring respiration in mitochondria isolated from the hippocampus of young mice 24 h after intraperitoneal injection of 0.33 mg/kg LPS (Control, n = 5; LPS, n = 5). **(E)** OCRs were comparable at all states between groups (Student's t-test). **(F)** Respiratory control ratios were comparable between groups (Student's t-test). **(G**–**I)** Hippocampal mRNA expression levels of inflammatory cytokines were measured 6, 24, and 48 h after injection of 0.04 mg/kg LPS in old-aged mice. **(G, H)** Expression levels were not affected 6 h (Student's t-test; Control, n = 3; LPS, n = 5) and 24 h (Student's t-test; Control, n = 4; LPS, n = 5) after LPS injection. **(I)** IL-1β mRNA expression level was significantly increased 48 h after LPS injection (Kruskal-Wallis test, p = 0.009; Control, n = 5; LPS, n = 5). **(J)** The mRNA expression levels of cytokines in the hippocampus of young mice were not affected 48 h after 0.33 mg/kg LPS (Student's t-test; Control, n = 5; LPS, n = 6). **(K)** Western blot analysis of inflammasome components in young and old mice. Representative Western blot images (left) and summary data (right). The number in the left-hand image indicates protein molecular weight (kDa). The expression level of pro-IL-1β (Kruskall-Wallis test, p = 0.014; Young, n = 5; Old, n = 4) and cleaved-caspase1 (Kruskall-Wallis test, p = 0.014; Young, n = 5; Old, n = 4) were significantly increased in aged mice (Young, n = 5; Old n = 4). Full Western blot images are provided in Supplementary Fig. 2. Values are presented as means ± SD (*p < 0.05, **p < 0.01, n.s., not significant).

To investigate the effects of systemic inflammation on mitochondrial function in the aged brain, we next injected old-age mice with low-dose (0.04 mg/kg) LPS. This dose of LPS is significantly lower than that used in previous studies, as the goal of our study was to model a clinically relevant level of systemic inflammation in old-age patients. Unlike the case for higher doses, we found that LPS at a dose of 0.04 mg/kg did not cause mortality (Table 1) but was still capable of causing mitochondrial dysfunction 24 h after LPS injection, as evidenced by significant decreases in uncoupled respiration (state3u) and RCR (state3u/state4o) (Fig. 2A–C). To further confirm that changes in mitochondria function induced by low-dose LPS were age-dependent, we also injected young mice with low-dose LPS. Because a previous study showed that aged mice are at least 6.5-times more sensitive to the toxic effects of LPS than old mice [42], we injected young mice with a higher dose of LPS (0.33 mg/kg). Interestingly, this dose of LPS induced no change in mitochondrial respiration in young mice (Fig. 2D–F), suggesting that mitochondria in the aged brain are more sensitive to systemic inflammation.

**Table 1:** Intraperitoneal LPS injection induced mortality at doses greater than 0.04 mg/kg in old-age mice.

Dose	0.33 mg/kg	0.2 mg/kg	0.16 mg/kg	0.08 mg/kg	0.04 mg/kg
**Mortality**	3/6 (50%)	4/10 (40%)	5/7 (71%)	2/6 (33%)	0/5 (0%)

### 3.3 LPS-induced mitochondrial dysfunction preceded the increase in IL-1β gene transcription in old-age mice

To determine whether LPS-induced mitochondrial dysfunction was associated with neuroinflammation, we next evaluated the hippocampal expression of inflammatory cytokines. While there were no changes in the expression of cytokines at 6 and 24 h after LPS injection (Fig. 2G and H), we discovered a significant increase in IL-1β gene transcription 48 h after LPS injection (Fig. 2I). Since mitochondrial respiration was significantly decreased 24 h after LPS injection in old mice (Fig. 2A–C), it is possible that mitochondrial dysfunction acts as a stage-setting event necessary for the late IL-1β expression (Fig. 2I). Our results in young mice also support the importance of mitochondrial dysfunction, as there was no increase in gene expression of cytokines 48 h after low-dose LPS injection (Fig. 2J). To further identify a possible mechanism for the delayed increase in IL-1β gene transcription in aged mice, we compared the expression of inflammasome components between young and old mice. Although there was no difference in the expression level of NLRP3, protein levels of pro-IL1β and cleaved caspase1 were significantly increased in old-aged mice (Fig. 2K, Suppl Fig. 2). As mitochondrial dysfunction, a representative hallmark of aging, is involved in the activation of the inflammasome signaling pathway [57,58], it is possible that low-dose LPS-induced mitochondria dysfunction in the hippocampus activated the pre-enhanced inflammasome signaling in aged mice, thus inducing the delayed expression of IL-1β.

### 3.4 Low-dose LPS induces cognitive impairments only in old-age mice

**Fig. 3:**
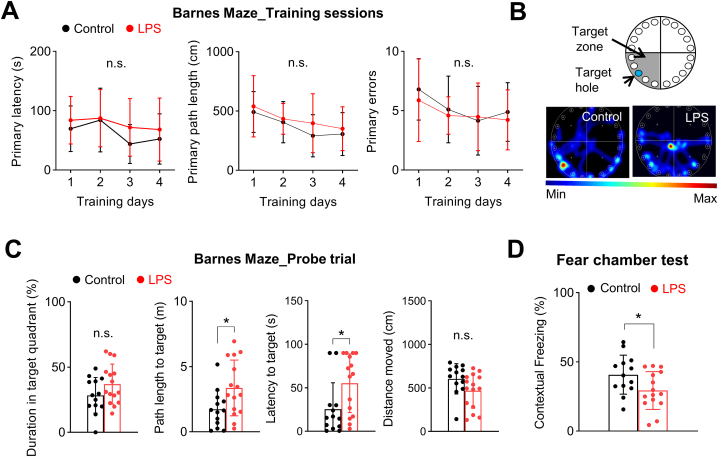
Low-dose LPS injection induces cognitive impairments in old-age mice. Barnes maze test and Fear chamber test was performed to measure learning and memory after LPS injection (Control, n = 13; LPS, n = 15). **(A)** LPS injection did not affect primary latency, length, or errors during training sessions. **(B, C)** Barnes maze probe trial. **(B)** Representative heatmap images. **(C)** LPS injection significantly increased path length and latency in the probe test (p = 0.021, Student's t-test). **(D)** LPS injection significantly reduced contextual memory in the fear-chamber test (p = 0.040, Student's t-test). Values are presented as means ± SD (*p < 0.05, n.s., not significant).

Previous studies have reported that neuroinflammation, especially increases in IL-1β, is associated with deficits in synaptic plasticity and cognitive impairments [59,60]. Thus, we next examined whether low-dose LPS was also capable of inducing cognitive impairments in old-age mice by performing Barnes maze and fear-chamber tests (Fig. 3). We first found that, although 0.04 mg/kg LPS did not cause mortality, it could induce significant weight changes indicating the development of systemic inflammation (Suppl. Fig. 3). The Barnes maze test was performed 2 days after LPS injection, the same time point associated with increased neuroinflammation (Fig. 3A–C). There were no differences in primary latency, path length, or number of errors between groups during the training period (Fig. 3A). However, although the time spent in the target zone were comparable, the distance and time required to find the escape hole were significantly longer in old-age mice that received LPS injection during the probe trial (Fig. 3B and C). Such discrepancies in the probe trial results may be due to differences in the nature of the variables, as path length and latency measures more precise memory compared to quadrant time. Cognitive function was also measured using the fear-chamber test, which revealed that contextual fear memory was significantly reduced in LPS injected old-age mice (Fig. 3D). Again, we found no evidence of cognitive dysfunction after low-dose LPS injection in young mice (Suppl. Fig. 4). These results further support the idea that mitochondrial dysfunction and neuroinflammation are important mechanisms underlying cognitive impairment after systemic inflammation in the aged brain.

### 3.5 Low-dose LPS injection in young mice pretreated with rotenone increased the expression of inflammatory cytokines and impaired learning and memory

**Fig. 4:**
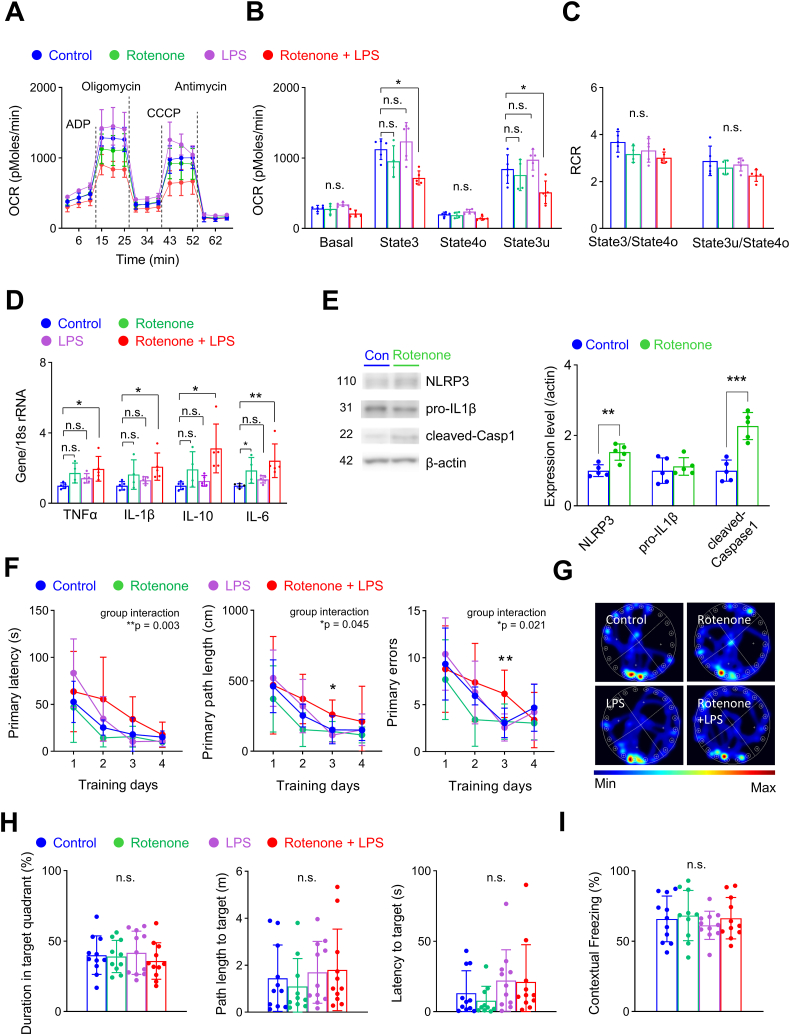
Low-dose LPS injection increases gene expression of inflammatory cytokines and induces learning impairment in young mice pretreated with rotenone. **(A**–**C)** Mitochondrial respiration was measured 24 h after LPS injection (Control, n = 5; LPS, n = 5; Rotenone, n = 4; Rotenone + LPS, n = 5). **(A)** Mitochondrial OCR was measured while sequentially adding ADP, oligomycin, CCCP, and antimycin; dotted lines indicate the addition of compounds. **(B)** Quantification of OCR after excluding non-mitochondrial respiration. Coupled (state3) and uncoupled (state3u) respiration were significantly decreased in mice that received both rotenone pretreatment and LPS injection (state3, p = 0.004; state3u, p = 0.003; one-way ANOVA with post hoc Tukey test). **(C)** RCR was not affected by rotenone or LPS injections (one-way ANOVA with post hoc Tukey test). **(D)** mRNA expression levels of cytokines, including TNFα (p = 0.033, one-way ANOVA with post hoc Tukey test), IL-1β (p = 0.021), IL-10 (p = 0.017) and IL-6 (p = 0.006; Kruskal-Wallis test), were significantly increased in mice that received both rotenone pretreatment and LPS injection (Control, n = 5; LPS, n = 5; Rotenone, n = 4; Rotenone + LPS, n = 5). **(E)** Western blot analysis of inflammasome components after rotenone pretreatment in young mice (Control, n = 5; Rotenone, n = 5). Representative Western blot images (left) and summary data (right). The number in the left-hand image indicates protein molecular weight (kDa). The expression level of NLRP3 (Student's t-test, p = 0.003) and cleaved-caspase1 (Student's t-test, p < 0.001) were significantly increased after rotenone pretreatment. Full Western blot images are provided in Supplementary Fig. 5. **(F–I)** Cognitive function was evaluated using the Barnes maze test and fear chamber test (Control, n = 11; Rotenone, n = 10; LPS, n = 11; Rotenone + LPS, n = 11). **(F)** Mice that received both rotenone pretreatment and LPS injection showed significant differences during training trials (group interaction, primary latency, p = 0.003; primary length, p = 0.045; primary errors, p = 0.021). **(G, H)** Representative heatmap images of the probe test. There were no significant differences between groups (one-way ANOVA, Kruskall-Wallis test). **(H)** There was no significant difference in contextual memory in the fear chamber test (one-way ANOVA). Values are presented as means ± SD (*p < 0.05, **p < 0.01, n.s., not significant).

To further investigate the importance of mitochondrial dysfunction in the development of neuroinflammation during low-level systemic inflammation, we pre-treated young mice with rotenone (2.5 mg/kg, i.p.) for 3 consecutive days before LPS injection. Rotenone induces mitochondrial dysfunction by inhibiting mitochondrial complex I, thus suppressing the electron transport chain and ATP production [44]. We found that only the combination of rotenone pretreatment and LPS injection reduced mitochondrial respiration (Fig. 4A–C). Although there were no changes in RCRs, both coupled (state3) and uncoupled (state3u) mitochondrial respiration were significantly reduced (Fig. 4B and C). Most importantly, the combination of rotenone pretreatment and LPS injection significantly increased gene expression of cytokines in the hippocampus of young mice (Fig. 4D). Interestingly, we also found that rotenone pretreatment increased the expression of components of the inflammasome signaling pathway NLRP3 and cleaved-caspase1 (Fig. 4E, Suppl Fig. 5). Consistent with old-aged mice, this may act as a mechanism for the increased expression of cytokines after low after low-dose LPS in young mice. To further confirm whether rotenone pretreatment could also induce cognitive impairment after low-dose LPS, we next performed the Barnes maze test and fear chamber test (Fig. 4F–H). Mice that received both rotenone pretreatment and low-dose LPS showed impaired learning during the training sessions of the Barnes maze test in primary latency, path length, and errors (Fig. 4F). However, there was no significant difference in the probe trial and the fear chamber test (Fig. 4G–I). Our results further suggest that mitochondrial dysfunction is involved in the activation of inflammasomes, increased expression of inflammatory cytokines, and subsequent cognitive impairments after low-dose LPS-induced systemic inflammation.

### 3.6 Continuous increase in cytokine mRNA levels despite recovery of mitochondrial function after low-dose LPS injection in old-age mice

**Fig. 5:**
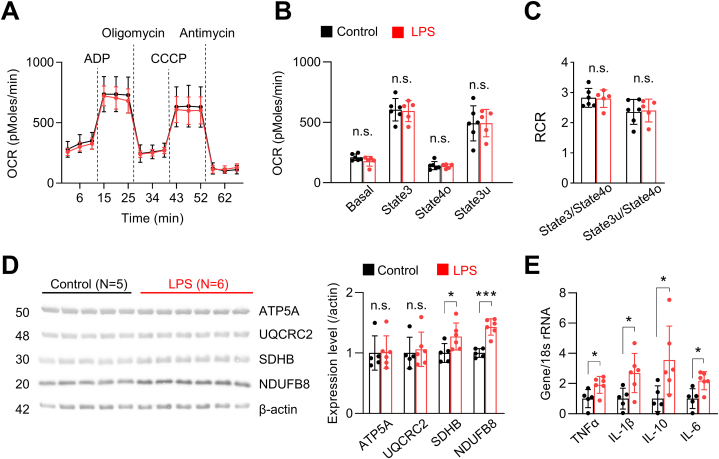
Increased cytokine mRNA levels persist in old-age mice despite recovery of mitochondrial function after low-dose LPS injection. **(A**–**C)** Mitochondrial function was evaluated in old-age mice by measuring respiration in mitochondria isolated from the hippocampus 12 days after LPS injection (Control, n = 6; LPS, n = 5). **(A)** Mitochondrial OCR was measured while sequentially adding ADP, oligomycin, CCCP, and antimycin; dotted lines indicate the addition of compounds. **(B)** Quantification of OCR after excluding non-mitochondrial respiration. OCRs in all states were comparable between groups (Student's t-test). **(C)** RCRs were comparable between groups (Student's t-test). **(D)** Western blot analysis of mitochondrial complex subunits in hippocampal samples. Expression levels of NDUFB8 (subunit of complex I) and SDHB (subunit of complex II) were significantly increased 12 days after LPS injection in old-age (n = 6), but not young (n = 5), mice (SDHB, p = 0.049, Student's t-test; NDUFB8, p < 0.001, Kruskal-Wallis test). The number in the left-hand image indicates protein molecular weight (kDa). Full Western blot images are provided in Supplementary Fig. 6. **(E)** mRNA expression levels of cytokines, including TNFα (p = 0.029), IL-1β (p = 0.028), IL-10 (p = 0.041) and IL-6 (p = 0.012), were significantly increased 12 days after LPS injection in the hippocampus of old-age mice (Student's t-test; Control, n = 5; LPS, n = 6). Values are presented as means ± SD (*p < 0.05, ***p < 0.001, n.s., not significant).

To further investigate the persistence of low-dose LPS effects, we next evaluated mitochondrial function 3 days after behavioral experiments (i.e., 12 days after LPS injection) in a subset of mice. We discovered that LPS-induced mitochondrial dysfunction was transient in old-age mice, as shown by the return of mitochondrial respiration levels and RCRs to normal (Fig. 5A–C). We also discovered that expression levels of OXPHOS complex subunits I (NDUFB8) and II (SDHB) were significantly increased compared with control mice (Fig. 5D, Suppl Fig. 6). Most importantly, in contrast to mitochondrial function, which recovered, cytokine mRNA expression levels remained significantly elevated (Fig. 5E). Whereas only IL-1β was increased 48 h after low-dose LPS injection, multiple inflammatory cytokines (TNFα, IL-1β, IL-10, and IL-6) were increased 12 days after LPS injection (Fig. 5E). Our results imply that, unlike mitochondrial dysfunction, expression of inflammatory cytokines continue to increase after a low-level systemic insult in old-age mice. The delayed and continuous rise in inflammatory cytokines after low-dose LPS injection might explain the late display of cognitive impairment the late probe trial and fear chamber test (Fig. 3).

## 4 Discussion

In the present study, we demonstrated mitochondrial dysfunction and increased gene expression of cytokines in old-age mice after a single injection of 0.04 mg/kg LPS—a low dose that produces effects that mirror the systemic inflammation often seen in old-age patients. Importantly, our results confirm that a clinically relevant, low-level systemic inflammatory insult may induce long-lasting neuroinflammation in the old-age brain and that mitochondrial dysfunction precedes the development of neuroinflammation. Notably, Barnes maze and fear chamber tests revealed that these changes were accompanied by learning and memory deficits.

Very few studies have evaluated the effects of low-dose LPS in old-age mice [61]. While symptoms of illness have been reported at doses as low as 0.02 mg/kg, the association of LPS exposure with neuroinflammation in old-age mice has primarily been investigated at higher doses capable of causing mortality [61,62]. In contrast, the effects of low-dose LPS in young mice have been relatively well studied [36,[63], [64], [65]]. Such studies have reported that LPS at a dose of 0.1 mg/kg (i.p.) is capable of acutely increasing cytokine levels in the hippocampus and inducing cognitive impairments (depression-like behavior, impaired learning and memory) [36,63,65]. Interestingly, our results are inconsistent with these studies, as we found no increases in cytokine levels or cognitive deficits after LPS injection in young mice, despite using a higher dose of LPS (0.33 mg/kg). However, various factors may affect the expression of inflammatory cytokines, such as the dose and administration route, LPS types, and animal strain [40]. Also, inconsistences in behavioral consequence of low-dose LPS may reflect behavioral effects of LPS-induced sickness, which may last more than 24 h [66]. Because sickness may affect the animal's performance during behavioral assays, we evaluated cognitive function 48 h after LPS injection, a contrast with previous studies that measured behavioral changes shortly after LPS injection [36,63,65].

Previous studies have reported inconsistent results regarding age-related mitochondrial dysfunction in the brain, despite a recognition that such dysfunction is an important hallmark of aging. While several studies have shown reduced oxygen consumption by neuronal mitochondria in old-age mice [38,39], others have reported relatively well-maintained mitochondrial function [53]. In the present study, we discovered significant reductions in mitochondrial RCRs in old-age mice that are potentially indicative of general mitochondrial function [48]. One possible explanation for such inconsistences regarding age-related changes in mitochondrial function may be differences in the locations of studied mitochondria. While one previous study measured synaptic mitochondrial function, reporting that it was well maintained in old-age mice [53], others have measured function in mitochondria isolated from whole brain tissues [38,39]. Another explanation could be the differences in the age of both young and old mice. Although there is no clear standard for defining ‘old age’ in rodents, most studies used rodents between the age of 18–24 months. In our present study, experiments were performed using 18-20 months-old mice. Thus, we may have discovered more significant changes if we used older animals (24 months old) since mitochondrial function may progressively decline. However, recent studies also suggest that age-dependent changes in mitochondrial metabolism are biphasic. Studies show that mitochondrial activity may first increase up to middle-age and subsequently decline with further aging [67]. Thus, future studies of mitochondrial function should carefully consider the age of both young and old mice.

Previous studies have also reported age-related changes in mitochondrial dynamics [53,[68], [69], [70]]. One proteomic study using neuronal mitochondria isolated from the mouse forebrain reported a reduction in both fission (DRP1) and fusion (MFN1, OPA3) regulatory proteins in 24-month-old mice compared with 12-month-old mice [70]. However, another proteomic study by the same group using synaptic mitochondria reported upregulation of fusion regulatory proteins (OPA1, MFN1, MFN2) in old-age (24 months) versus young (12 months) mice [53]. Such differences may result from different effects of the aging process on synaptic and extra-synaptic mitochondria [71]. Interestingly, we also discovered increased expression of OPA1 in the hippocampus of old-age mice. It is difficult to directly compare our results with previous proteomic studies, given that we evaluated expression levels of dynamic regulators in hippocampal brain samples rather than isolated mitochondria. However, previous studies have suggested that mitochondrial fusion supports oxidative phosphorylation [69,72,73]; thus, the increase in OPA1 may act as a compensatory mechanism that serves to maintain mitochondrial respiration in old-age mice.

While our results are in line with previous studies targeting mitochondria to reduce neuroinflammation and subsequent cognitive impairments [50,74,75], we further discovered that mitochondrial dysfunction precedes the increase in IL-1β mRNA levels after clinically relevant systemic inflammation in the aged brain. Unfortunately, we were unable to show a direct link between mitochondrial dysfunction, neuroinflammation, and cognitive impairments after LPS injection. Although previous studies suggest a strong link [22,57,76,77], it is possible that mitochondrial dysfunction, neuroinflammation, and cognitive impairments are independent consequences. Mitochondrial dysfunction, a representative hallmark of aging, modulates neuroinflammation through multiple molecular mechanisms including the inflammasome signaling pathway [22,57,58]. Similar to a previous study reporting age-dependent increases in inflammasome proteins (NLRC4, ASC, caspase-1) [27], we also found increased expression of pro-IL-1β and cleaved-caspase1 in the aged brain. Such baseline activation of inflammasomes in aged mice may act as a stage-setting event for neuroinflammation development, as low-dose LPS deteriorated of mitochondria function can further activate inflammasome assembly and release of inflammatory cytokines. By injecting young mice to rotenone, we also provided indirect evidence as low-dose LPS increased mRNA expression of cytokines and induce cognitive impairments in young mice with preexisting mitochondrial dysfunction. Our results suggest that additional activation of the pre-enhanced inflammasome signaling pathway due to mitochondrial impairment may act as a significant mechanism for neuroinflammation after low-grade systemic inflammation.

It should also be noted is that we examined mitochondria isolated from the whole hippocampus, which limits our ability to identify the specific cell type that might be metabolically sensitive to low-dose LPS in the aged brain. Previous studies have shown that LPS can induce microglia to generate mitochondrial ROS (reactive oxygen species) and alter their mitochondrial dynamics. These changes are believed to be key regulators of neuroinflammation [78,79]. As studies also report altered energy metabolism (metabolic shift) after LPS-induced mitochondrial changes in microglia [80], it is likely that microglia are metabolically sensitive. However, a study using primary microglia culture showed that application of mitochondrial toxins did not enhance the expression of proinflammatory cytokines after LPS treatment [81]. Interestingly, another study showed that protecting mitochondrial function specifically in neurons could suppress IL-1β expression and microglia activation after LPS injection [50]. These results suggest that various cell types (microglia, neurons) are metabolically sensitive to LPS injection.

There are several other significant limitations in the present study, such as the lack of evidence regarding neuroinflammation at the protein level. Despite several studies reported a correlation between mRNA and protein expression levels of cytokines [82,83], others have shown showed early [84], delayed [85], and subtle [82] changes in protein expression compared to gene transcription levels. Despite the delayed and continuous increases in cytokine mRNA levels after low-dose LPS injection in aged mice, additional studies at the protein level may be necessary. The second limitation is the lack of female mice. Previous studies show superior mitochondrial function and antioxidant defenses in females, as sex hormones (17β-estradiol, progesterone) enhance brain mitochondria metabolism and reduce oxidative stress [86,87]. Therefore, female mice may be more vulnerable to age-dependent mitochondrial dysfunction caused by systemic inflammation due to significant reductions in sex hormones with aging [88]. However, previous studies also reported inconsistent results regarding sex-dependent mitochondrial differences in the aging brain [86]. Unfortunately, we were unable to perform experiments in both sexes due to the limited supply of aged mice. Another limitation is the lack of diverse brain regions. Although the hippocampus is sensitive to neuroinflammation [59,89,90], studies have shown different sensitivities to neuroinflammation between brain regions [91]. Thus, additional studies in diverse brain regions are needed to generalize our findings. Lastly, mitochondrial respiration measurements were performed only through complex II (in the presence of succinate and rotenone). As previous studies suggest that the activity of complex I may be more sensitive to aging [92], additional studies evaluating changes in complex 1-dependent mitochondrial respiration (NADH-linked mitochondrial respiration) are necessary to understand the effects of low-dose LPS in old-aged mice.

In conclusion, i.p. injection of low-dose LPS induced mitochondrial dysfunction followed by increased gene expression of cytokines in old age mice. Based on our results, it seems possible that reducing age-dependent mitochondrial dysfunction after low-grade peripheral inflammation may be effective in reducing secondary neuroinflammation and subsequent cognitive impairments. However, additional studies clarifying the association between mitochondrial dysfunction and neuroinflammation are needed to establish mitochondria as potential targets for preventing cognitive impairment after a low-level systemic inflammatory event.

## Data availability statement

Data will be made available on request.

## CRediT authorship contribution statement

**Yulim Lee:** Writing – review & editing, Writing – original draft, Methodology, Investigation, Data curation, Conceptualization. **Xianshu Ju:** Investigation, Formal analysis, Data curation. **Jianchen Cui:** Investigation, Data curation. **Tao Zhang:** Investigation, Data curation. **Boohwi Hong:** Software, Formal analysis, Data curation. **Yoon Hee Kim:** Supervision, Conceptualization. **Youngkwon Ko:** Supervision, Conceptualization. **Jiho Park:** Resources, Investigation, Data curation. **Chul Hee Choi:** Supervision, Resources, Conceptualization. **Jun Young Heo:** Writing – review & editing, Writing – original draft, Resources, Conceptualization. **Woosuk Chung:** Writing – review & editing, Writing – original draft, Supervision, Resources, Conceptualization.

## Declaration of competing interest

The authors declare that they have no known competing financial interests or personal relationships that could have appeared to influence the work reported in this paper.
