# Supplementary material for: Mitochondrial dysfunction precedes hippocampal IL-1β transcription and cognitive impairments after low-dose lipopolysaccharide injection in aged mice
Source: Heliyon. 2024 Mar 30;10(7):e28974. doi: 10.1016/j.heliyon.2024.e28974 (PMC11002287; doi:10.1016/j.heliyon.2024.e28974)
Supplement: Multimedia component 1 [file mmc1.pdf]

Supplementary Figures

S Fig 1

A

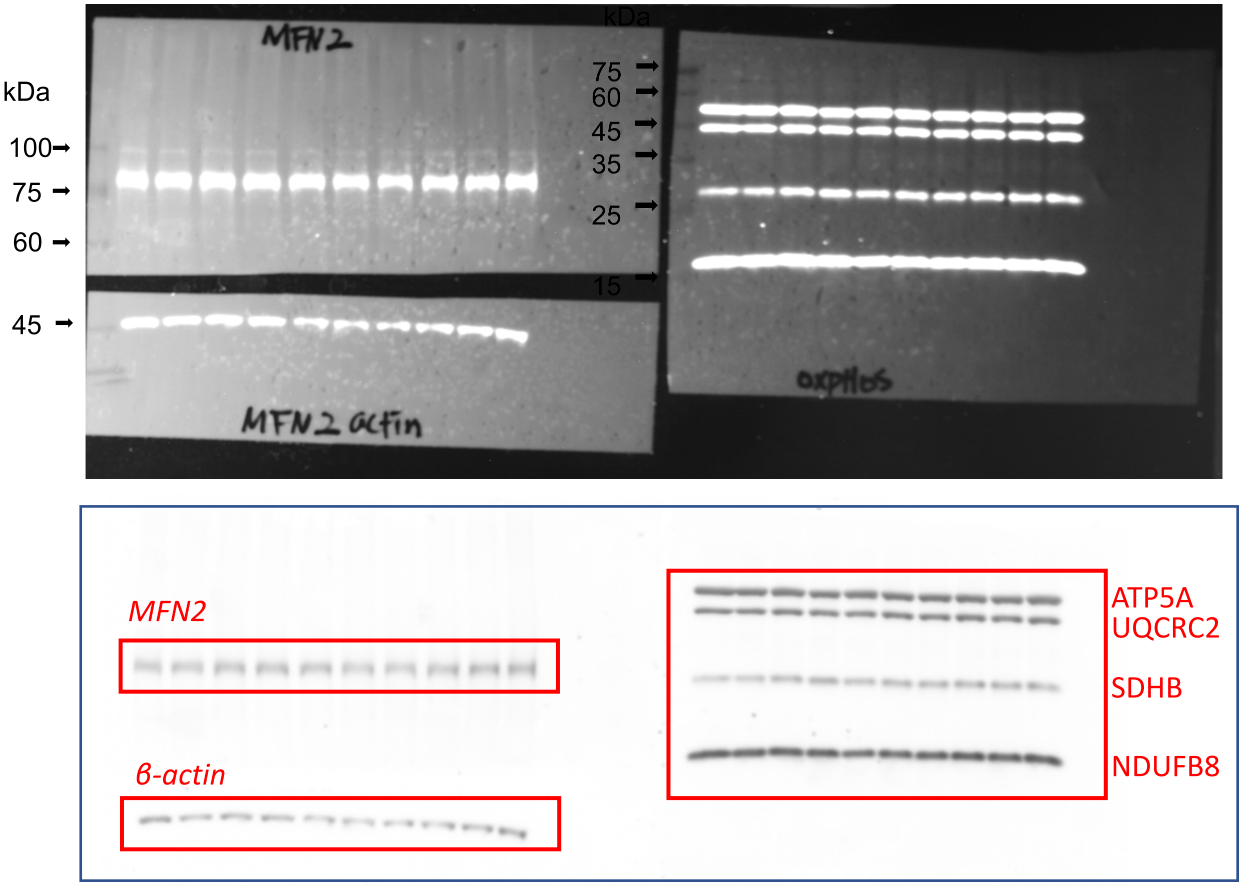

B

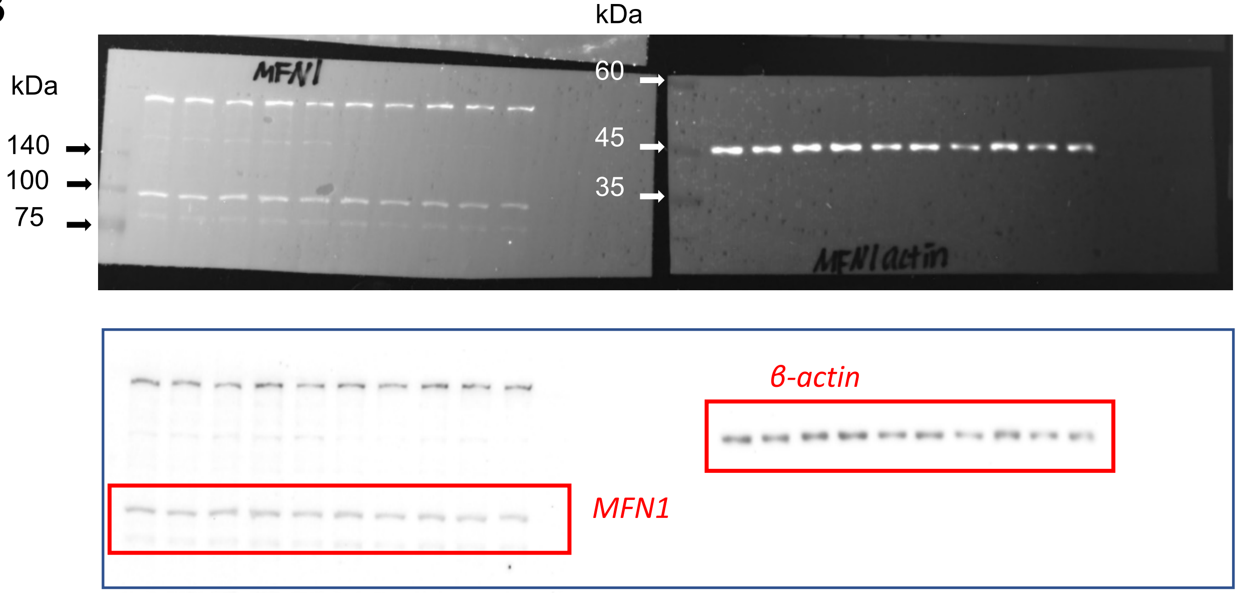

Continued on next page

S Fig 1 (continued)

C

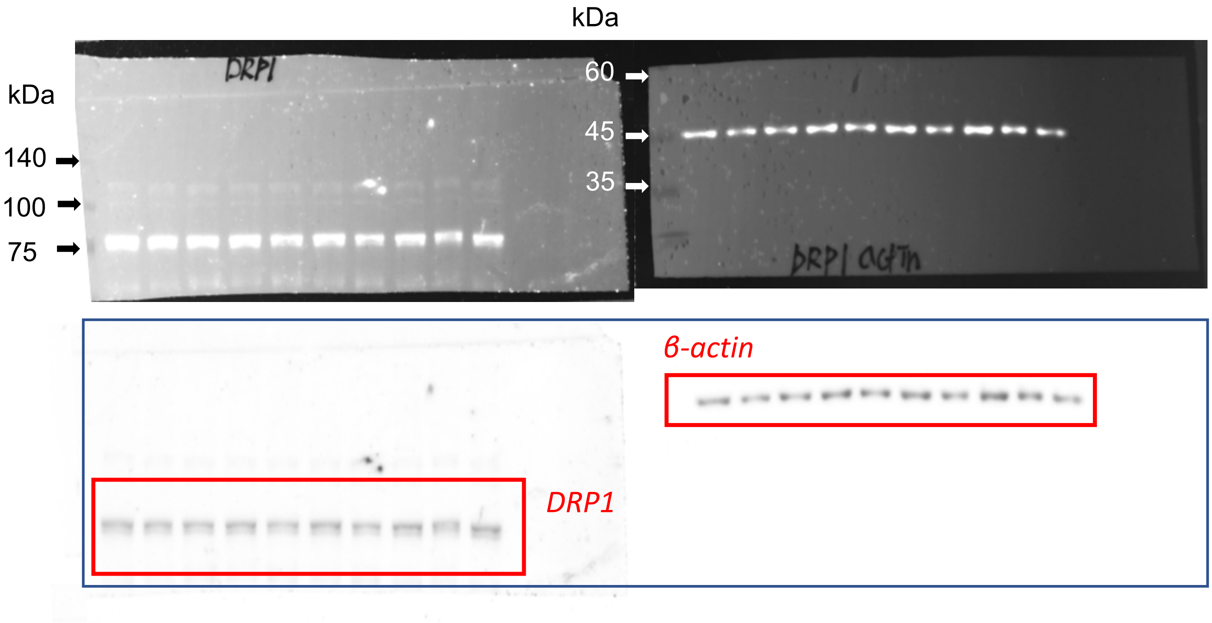

D

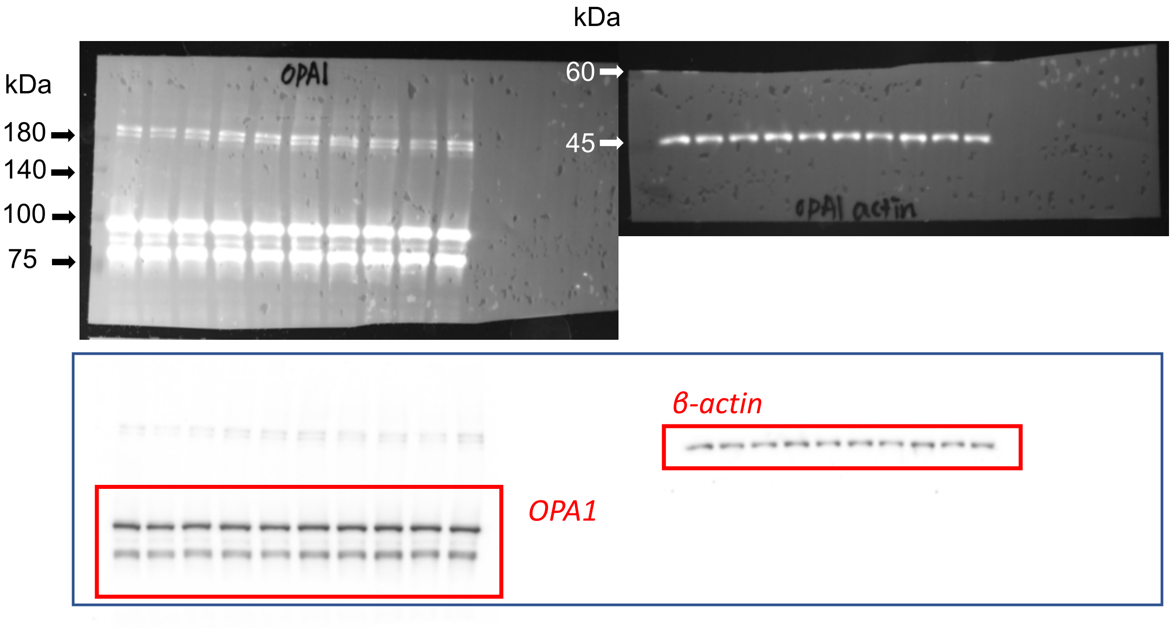

Suppl Figure 1. Full Western blot images used in Figure1.

Upper images show overexposed images used to visualize size markers at the left side of the images.

Lower images show actual images used to evaluate protein expression.

S Fig 2

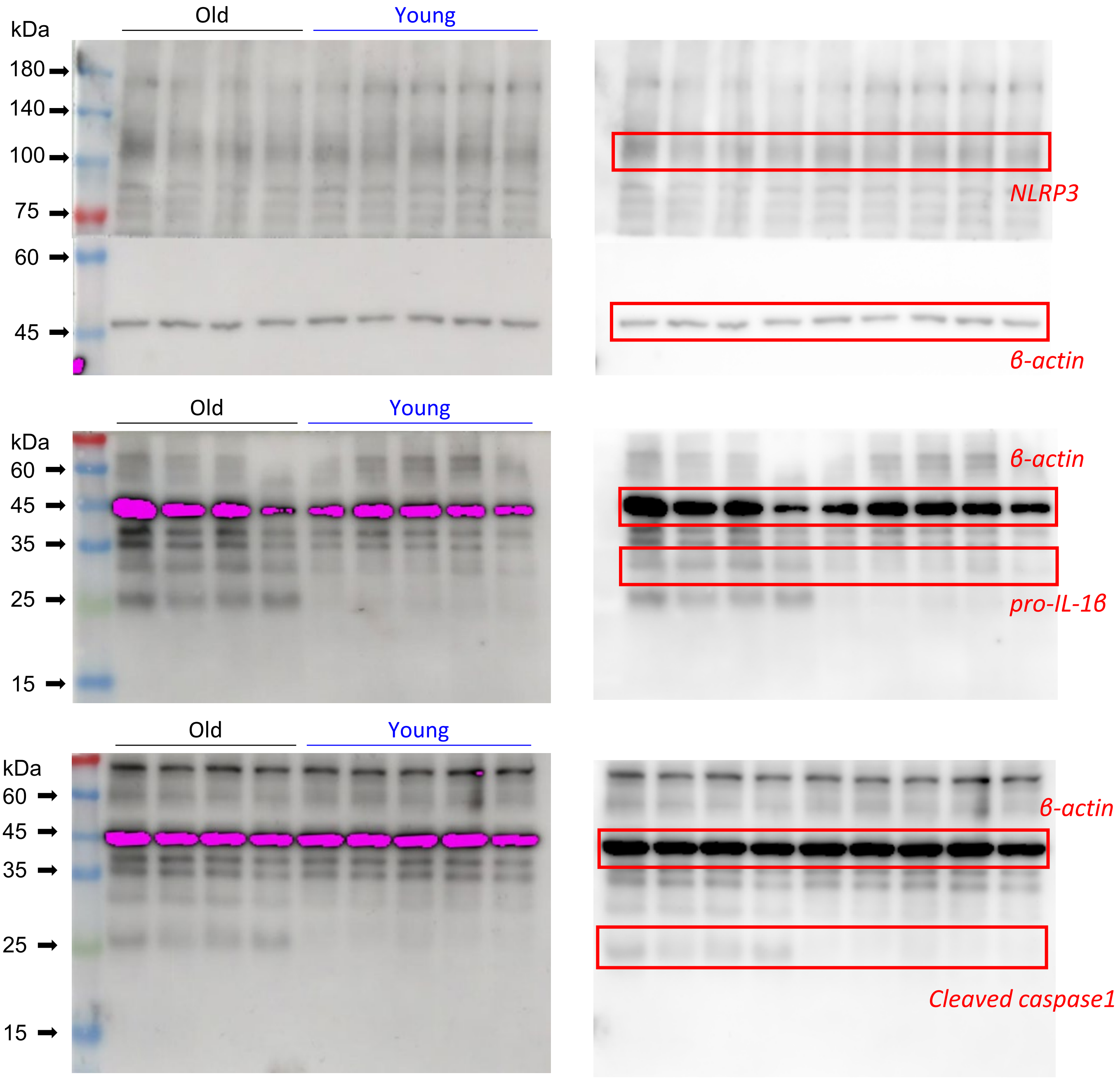

Suppl Figure 2. Full Western blot images used in Figure2.

S Fig 3

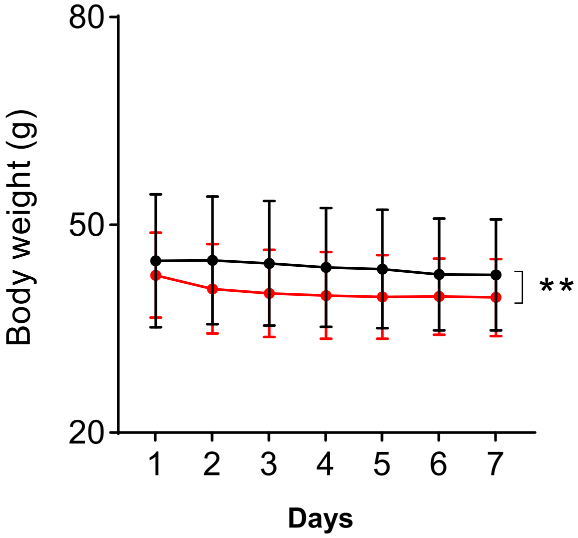

Suppl Figure 3. Low-dose LPS injection induced significant weight loss in old-age mice

Low-dose LPS caused significant changes in body weight (p = 0.001, nonparametric multiple comparisons test; Control, n = 12; LPS, n = 12). Values are presented as means ± SD, n.s., not significant, *p < 0.01.

S Fig 4

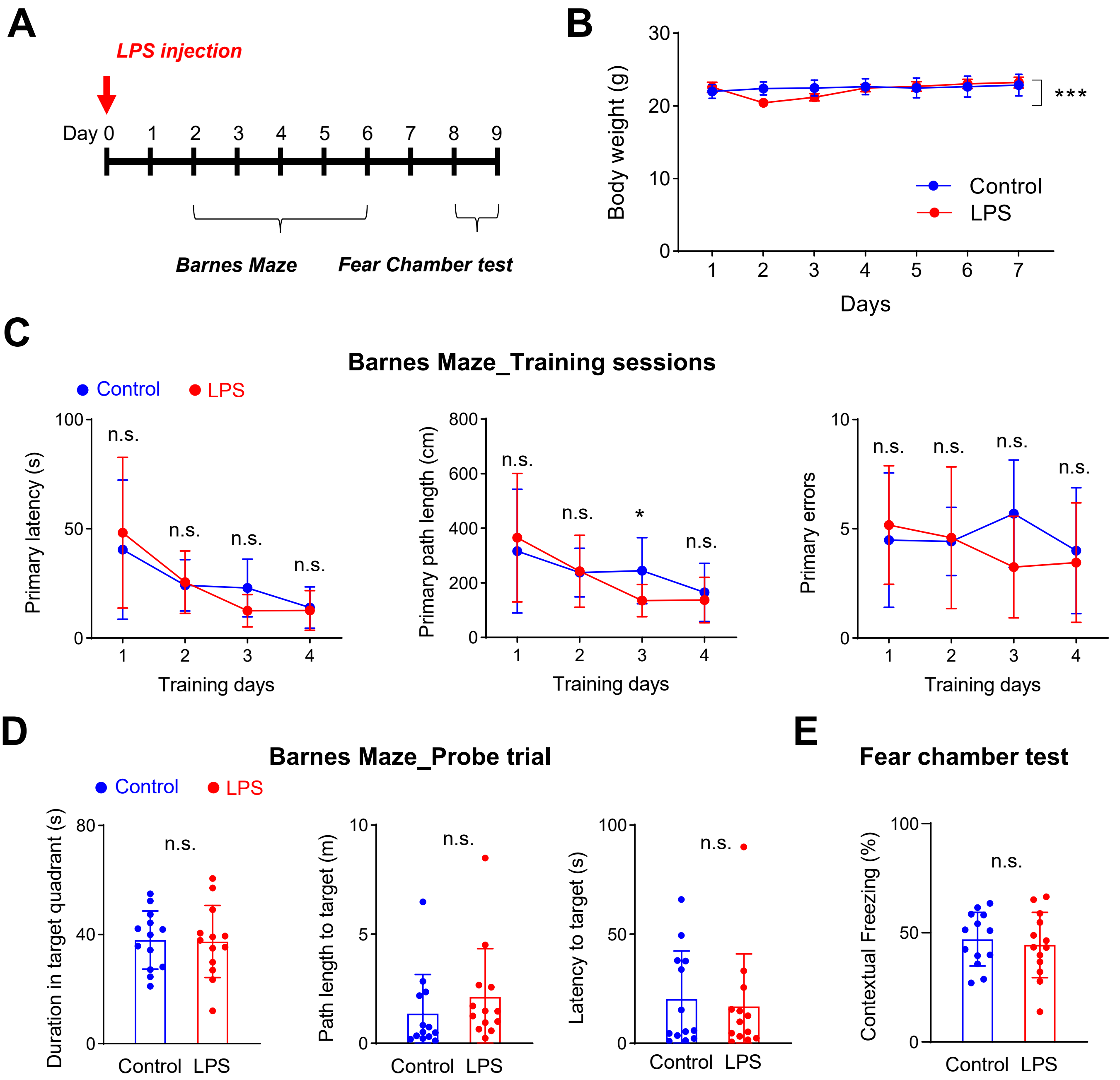

Suppl Figure 4. Low-dose LPS injection in young mice did not affect learning and memory in the Barnes maze and fear chamber test

(A) Timeline of behavioral assays performed after 0.33 mg/kg LPS injection in young mice. **(B)** Low-dose LPS caused significant changes in body weight ($p < 0.001$, RM-ANOVA; Control, n = 10; LPS, n = 11). **(C, D)** The Barnes maze test was performed to measure learning and memory after LPS injection (Control, n = 13; LPS, n = 13) **(C)** Although LPS injection did not affect primary and errors, there was a significant difference in primary path length. Unexpectedly, path length was lower in mice that received LPS injection ($p = 0.028$, RM-ANOVA). **(D)** Probe trial results were comparable between groups (Student's t-test, Kruskal-Wallis test). **(F)** LPS injection did not affect contextual memory in the fear chamber test (Student's t-test; Control, n = 13; LPS, n = 13). Values are presented as means ± SD, n.s., not significant, $*p < 0.05$.

S Fig 5

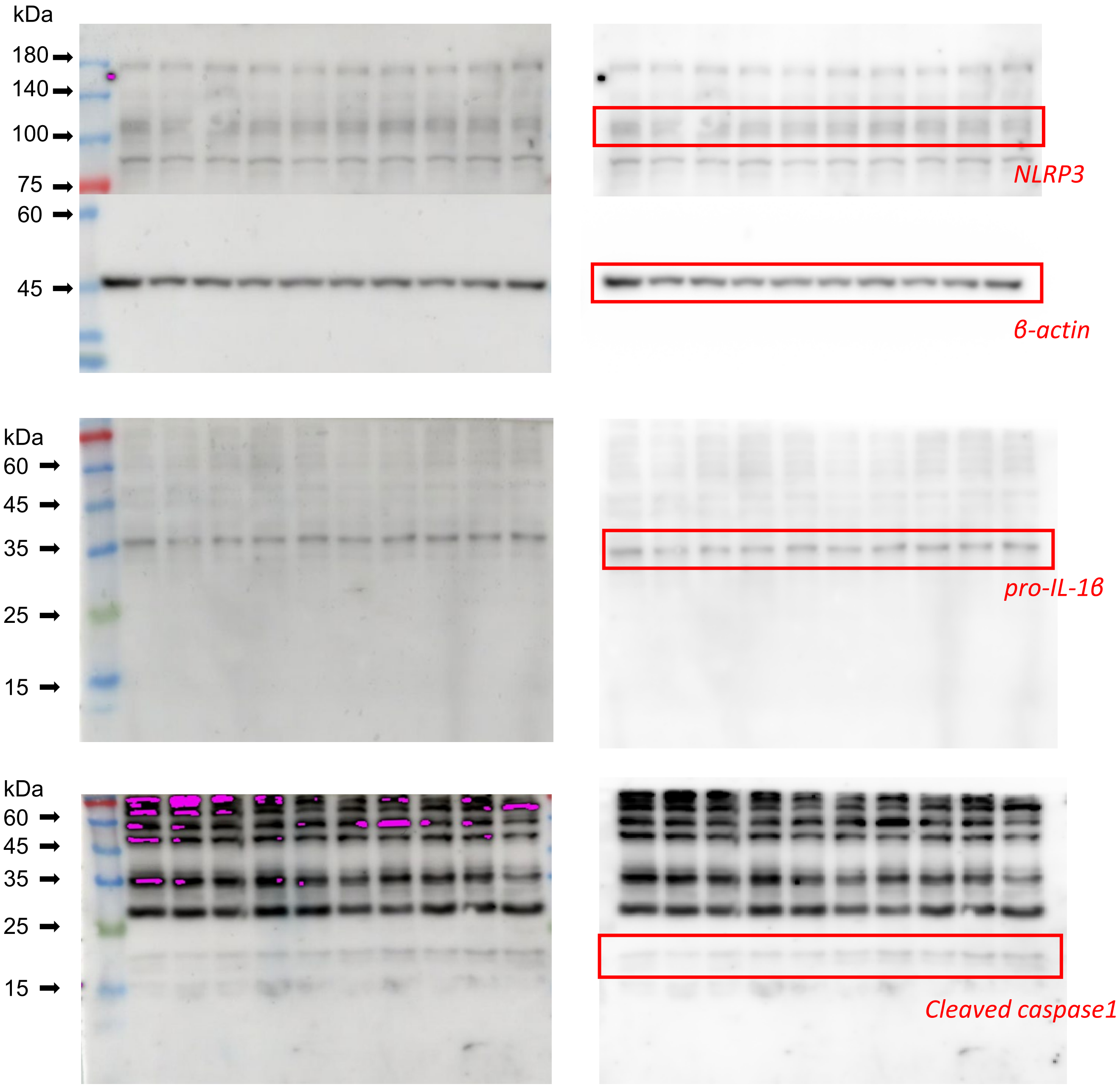

Suppl Figure 5. Full Western blot images used in Figure4.

S Fig 6

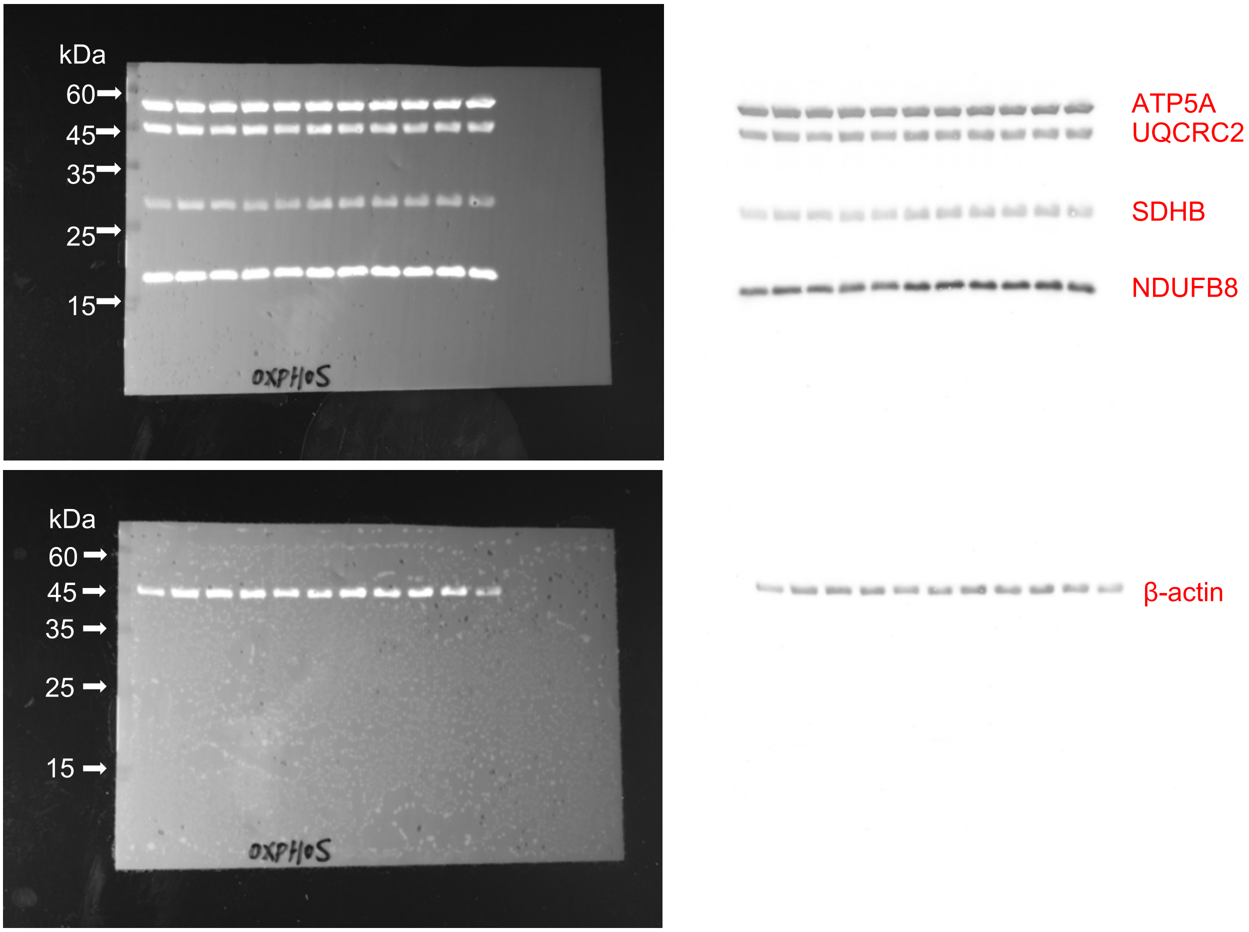

Suppl Figure 6. Full Western blot images used in Figure5.

Actin images were obtained after stripping the Western blot membrane.
