# Supplementary material for: Mitochondrial dysfunction precedes hippocampal IL-1β transcription and cognitive impairments after low-dose lipopolysaccharide injection in aged mice
Source: Heliyon. 2024 Mar 30;10(7):e28974. doi: 10.1016/j.heliyon.2024.e28974 (PMC11002287; doi:10.1016/j.heliyon.2024.e28974)
Supplement: Multimedia component 2 [file mmc2.pdf]

Supplementary data
Statistics

Figure1 Statistics

Fig1_B.OCR_Basal Data analysis

Data structure

```
str(d1)
```

```
## 'data.frame':    10 obs. of  3 variables:
##  $ subject: int  1 2 3 4 5 6 7 8 9 10
##  $ group  : chr  "young" "young" "young" "young" ...
##  $ OCR    : num  231 261 370 228 397 ...
```

Explorative data analysis with graphics

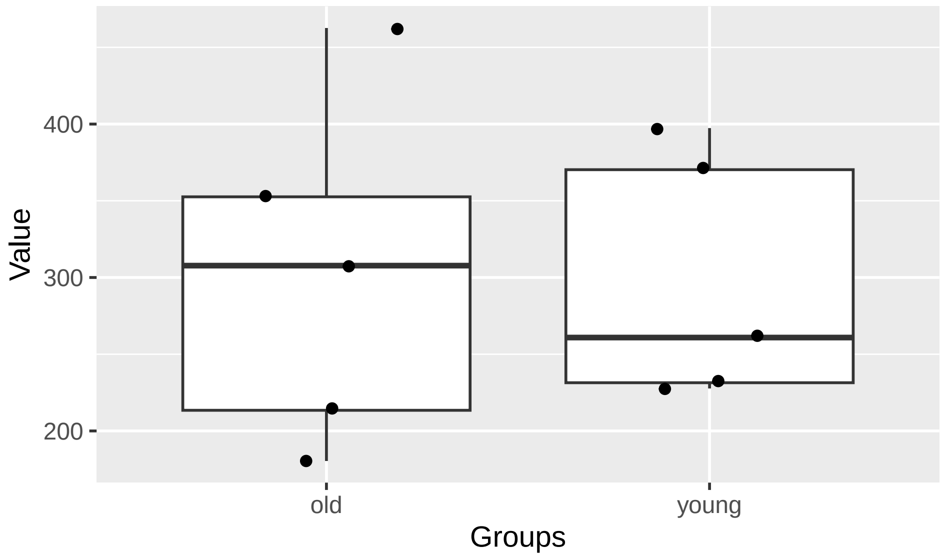


```
##
##  Welch Two Sample t-test
##
## data:  value by group
## t = 0.094143, df = 7.2265, p-value = 0.9276
## alternative hypothesis: true difference in means between group old and group young is not equal to 0
## 95 percent confidence interval:
##  -139.8266  151.4989
## sample estimates:
##   mean in group old mean in group young
##            303.3615            297.5253
```

```
## The result of t-test:
##  p = 0.928
##  A statistically significant difference do not exist between groups
```

Fig1_B.OCR_State3 Data analysis

Data structure

```
str(d1)
```

```
## 'data.frame':    10 obs. of  3 variables:
##  $ subject: int  1 2 3 4 5 6 7 8 9 10
##  $ group  : chr  "young" "young" "young" "young" ...
##  $ OCR    : int  711 795 1134 731 1174 521 1015 843 567 1158
```

Explorative data analysis with graphics

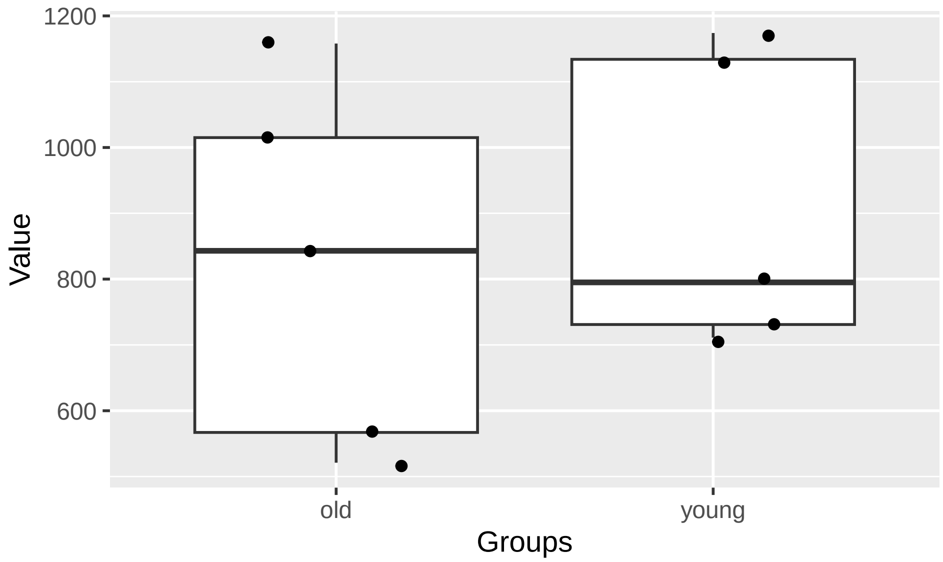


```
##
##  Welch Two Sample t-test
##
## data:  value by group
## t = -0.55182, df = 7.6964, p-value = 0.5967
## alternative hypothesis: true difference in means between group old and group young is not equal to 0
## 95 percent confidence interval:
##  -459.3239  282.9239
## sample estimates:
##   mean in group old mean in group young
##               820.8               909.0
```

```
## The result of t-test:
##  p = 0.597
##  A statistically significant difference do not exist between groups
```

Fig1_B.OCR_State4o Data analysis

Data structure

```
str(d1)
```

```
## 'data.frame':    10 obs. of  3 variables:
##  $ subject: int  1 2 3 4 5 6 7 8 9 10
##  $ group  : chr  "young" "young" "young" "young" ...
##  $ OCR    : num  136 163 210 133 215 ...
```

Explorative data analysis with graphics

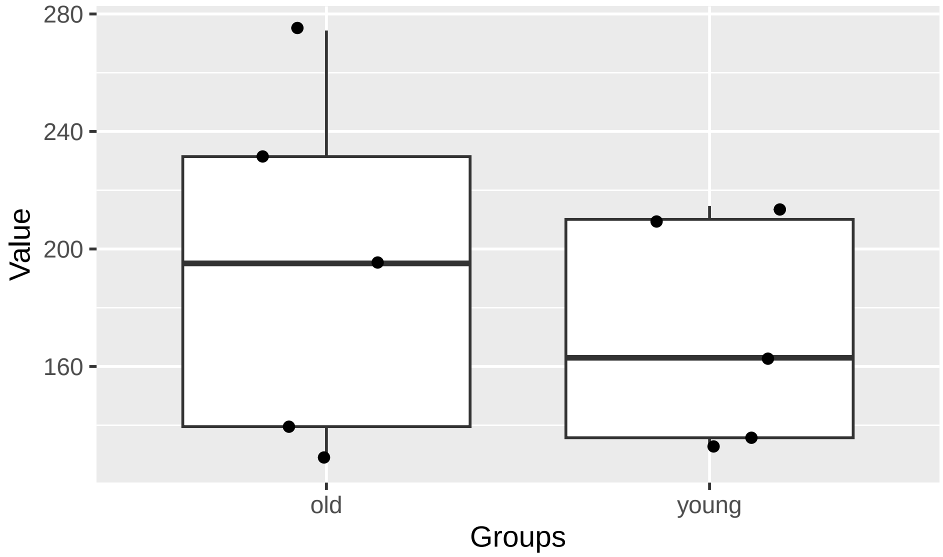


```
##
##  Welch Two Sample t-test
##
## data:  value by group
## t = 0.68558, df = 6.7975, p-value = 0.5157
## alternative hypothesis: true difference in means between group old and group young is not equal to 0
## 95 percent confidence interval:
##  -55.40384 100.26434
## sample estimates:
##   mean in group old mean in group young
##            193.6555            171.2252
```

```
## The result of t-test:
##  p = 0.516
##  A statistically significant difference do not exist between groups
```

Fig1_B.OCR_State3u Data analysis

Data structure

```
str(d1)
```

```
## 'data.frame':    10 obs. of  3 variables:
##  $ subject: int  1 2 3 4 5 6 7 8 9 10
##  $ group  : chr  "young" "young" "young" "young" ...
##  $ OCR    : num  612 699 1028 653 1053 ...
```

Explorative data analysis with graphics

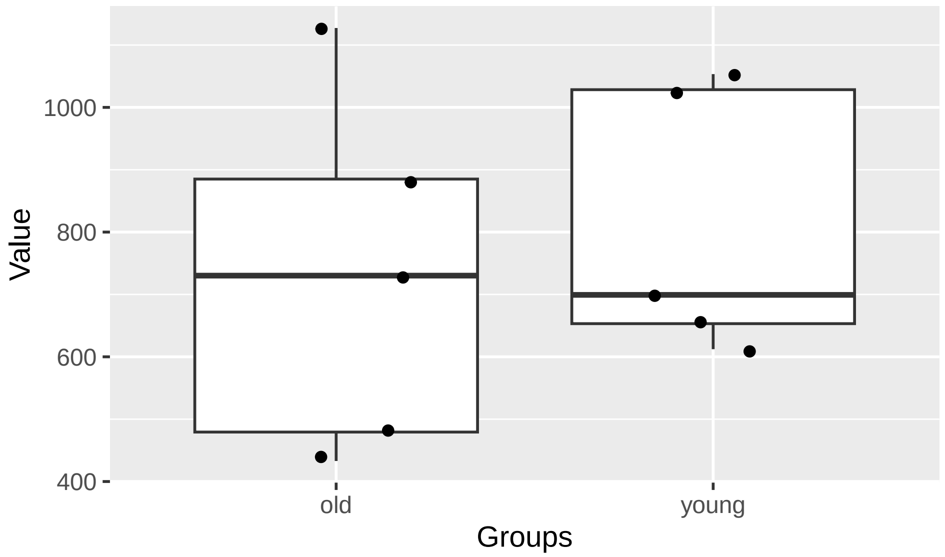


```
##
##  Welch Two Sample t-test
##
## data:  value by group
## t = -0.48781, df = 7.3754, p-value = 0.6399
## alternative hypothesis: true difference in means between group old and group young is not equal to 0
## 95 percent confidence interval:
##  -454.2312  297.5413
## sample estimates:
##   mean in group old mean in group young
##            730.9431            809.2881
```

```
## The result of t-test:
##  p = 0.64
##  A statistically significant difference do not exist between groups
```

Fig1_C.RCR Data analysis

Data structure

```
str(d1)
```

```
## 'data.frame':    10 obs. of  3 variables:
##  $ subject: int  1 2 3 4 5 6 7 8 9 10
##  $ group  : chr  "young" "young" "young" "young" ...
##  $ RCR    : num  3.58 3.38 3.71 3.71 3.68 ...
```

Explorative data analysis with graphics

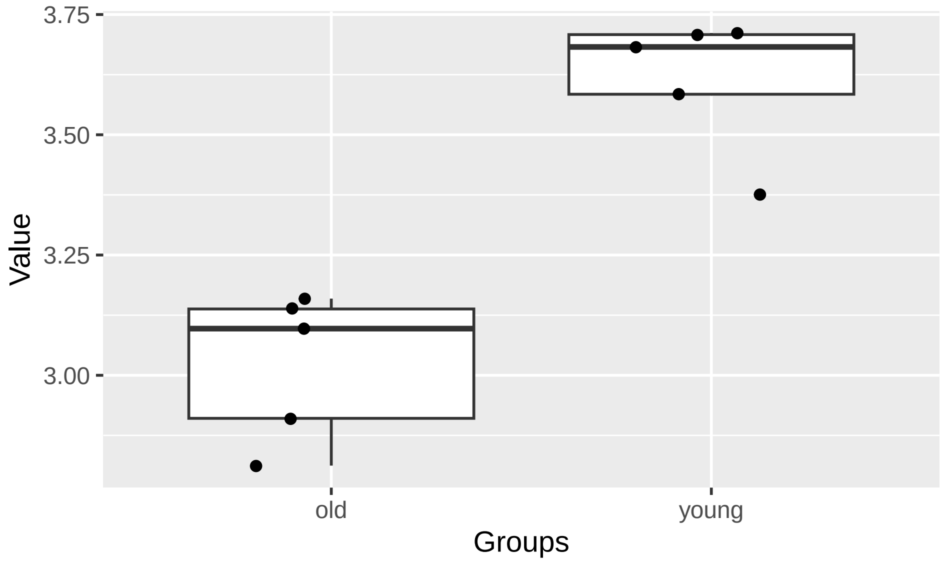


```
##
##  Welch Two Sample t-test
##
## data:  value by group
## t = -6.3079, df = 7.948, p-value = 0.0002372
## alternative hypothesis: true difference in means between group old and group young is not equal to 0
## 95 percent confidence interval:
##  -0.8049616 -0.3736136
## sample estimates:
##   mean in group old mean in group young
##            3.023421            3.612708
```

```
## The result of t-test:
##  p = 0
##  A statistically significant difference exist between groups
```

Fig1_C.RCRu Data analysis

Data structure

```
str(d1)
```

```
## 'data.frame':    10 obs. of  3 variables:
##  $ subject: int  1 2 3 4 5 6 7 8 9 10
##  $ group  : chr  "young" "young" "young" "young" ...
##  $ RCRu   : num  3.14 3.02 3.4 3.36 3.35 ...
```

Explorative data analysis with graphics

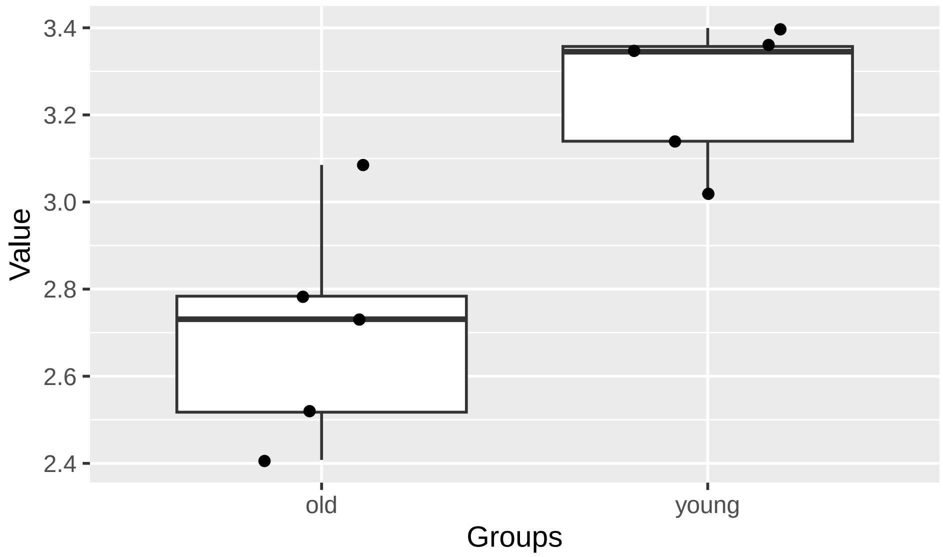


```
##
##  Welch Two Sample t-test
##
## data:  value by group
## t = -3.9502, df = 6.7345, p-value = 0.005976
## alternative hypothesis: true difference in means between group old and group young is not equal to 0
## 95 percent confidence interval:
##  -0.8769982 -0.2169100
## sample estimates:
##   mean in group old mean in group young
##            2.705039            3.251993
```

```
## The result of t-test:
##  p = 0.006
##  A statistically significant difference exist between groups
```

Fig1_D.Western Blot_ATP5A Data analysis

Data structure

```
str(d1)
```

```
## 'data.frame':    10 obs. of  3 variables:
##  $ subject: int  1 2 3 4 5 6 7 8 9 10
##  $ group  : chr  "young" "young" "young" "young" ...
##  $ ATP5A  : num  0.884 1.103 1.016 0.818 1.18 ...
```

Explorative data analysis with graphics

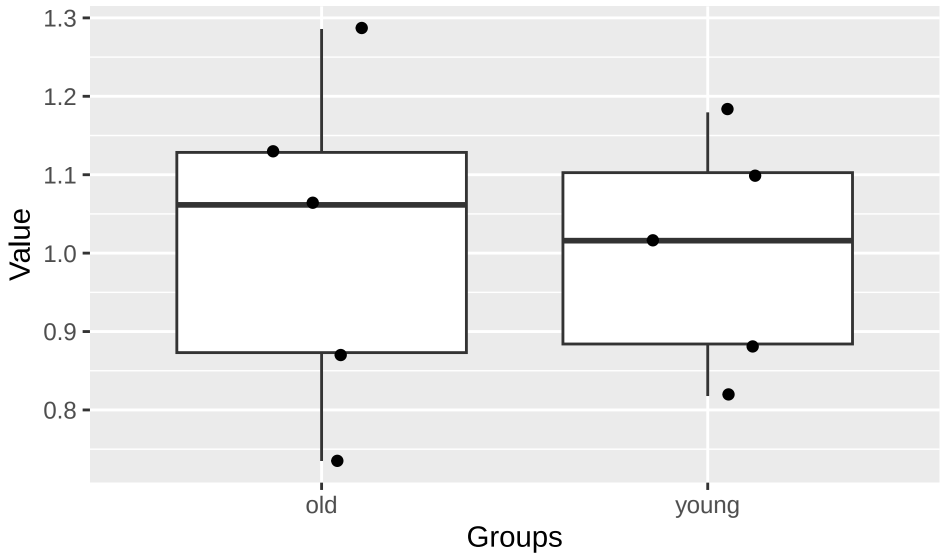


```
##
##  Welch Two Sample t-test
##
## data:  value by group
## t = 0.14269, df = 7.1192, p-value = 0.8905
## alternative hypothesis: true difference in means between group old and group young is not equal to 0
## 95 percent confidence interval:
##  -0.2603641  0.2939254
## sample estimates:
##   mean in group old mean in group young
##            1.016781            1.000000
```

```
## The result of t-test:
##  p = 0.89
##  A statistically significant difference do not exist between groups
```

Fig1_D.Western Blot_UQCRC2 Data analysis

Data structure

```
str(d1)
```

```
## 'data.frame':    10 obs. of  3 variables:
##  $ subject: int  1 2 3 4 5 6 7 8 9 10
##  $ group  : chr  "young" "young" "young" "young" ...
##  $ UQCRC2 : num  0.865 1.089 1.041 0.88 1.124 ...
```

Explorative data analysis with graphics

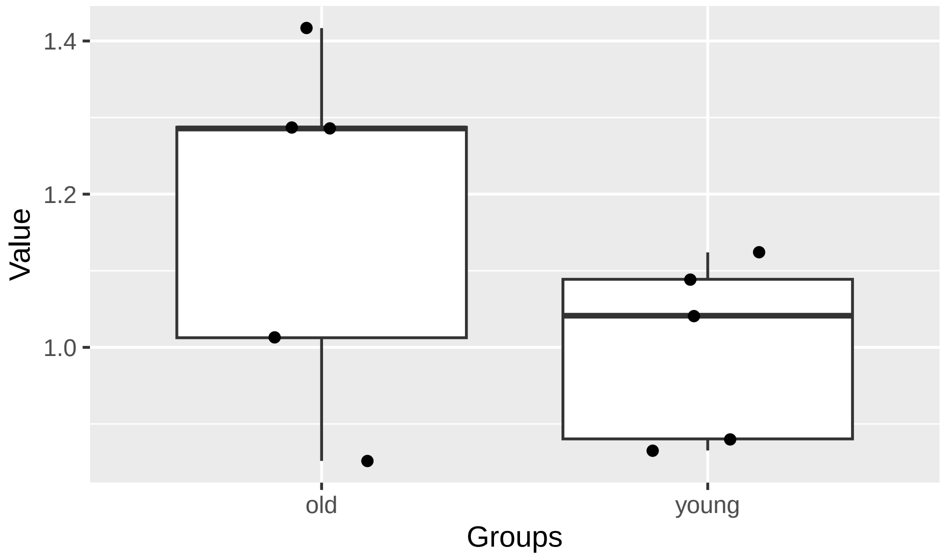


```
##
##  Welch Two Sample t-test
##
## data:  value by group
## t = 1.4662, df = 5.9999, p-value = 0.193
## alternative hypothesis: true difference in means between group old and group young is not equal to 0
## 95 percent confidence interval:
##  -0.1142738  0.4559622
## sample estimates:
##   mean in group old mean in group young
##            1.170844            1.000000
```

```
## The result of t-test:
##  p = 0.193
##  A statistically significant difference do not exist between groups
```

Fig1_D.Western Blot_SDHB Data analysis

Data structure

```
str(d1)
```

```
## 'data.frame':    10 obs. of  3 variables:
##  $ subject: int  1 2 3 4 5 6 7 8 9 10
##  $ group  : chr  "young" "young" "young" "young" ...
##  $ SDHB   : num  0.621 0.965 1.112 1.117 1.185 ...
```

Explorative data analysis with graphics

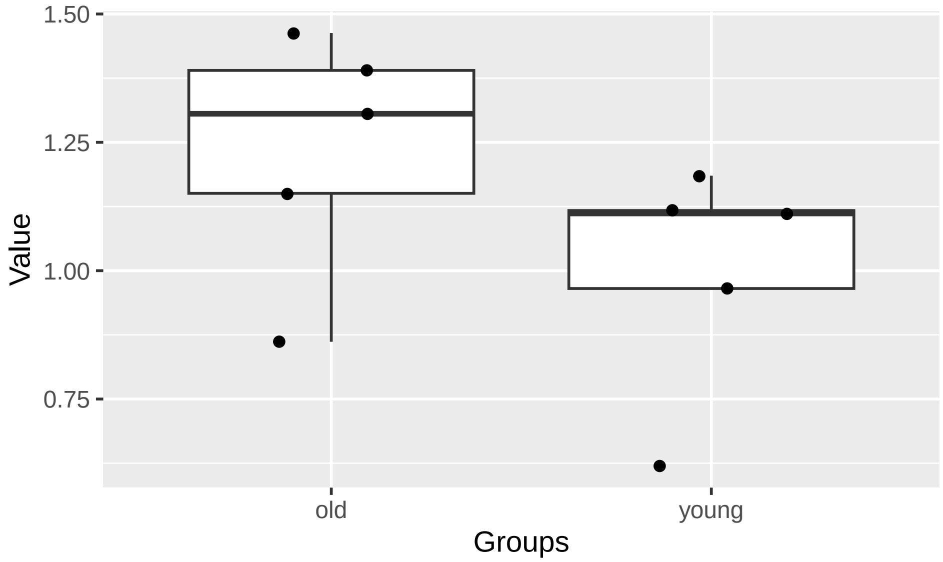


```
##
##  Welch Two Sample t-test
##
## data:  value by group
## t = 1.5919, df = 7.9784, p-value = 0.1502
## alternative hypothesis: true difference in means between group old and group young is not equal to 0
## 95 percent confidence interval:
##  -0.1052128  0.5735445
## sample estimates:
##   mean in group old mean in group young
##            1.234166            1.000000
```

```
## The result of t-test:
##  p = 0.15
##  A statistically significant difference do not exist between groups
```

Fig1_D.Western Blot_NDUFB8 Data analysis

Data structure

```
str(d1)
```

```
## 'data.frame':    10 obs. of  3 variables:
##  $ subject: int  1 2 3 4 5 6 7 8 9 10
##  $ group  : chr  "young" "young" "young" "young" ...
##  $ NDUFB8 : num  0.905 1.151 1.053 0.936 0.955 ...
```

Explorative data analysis with graphics

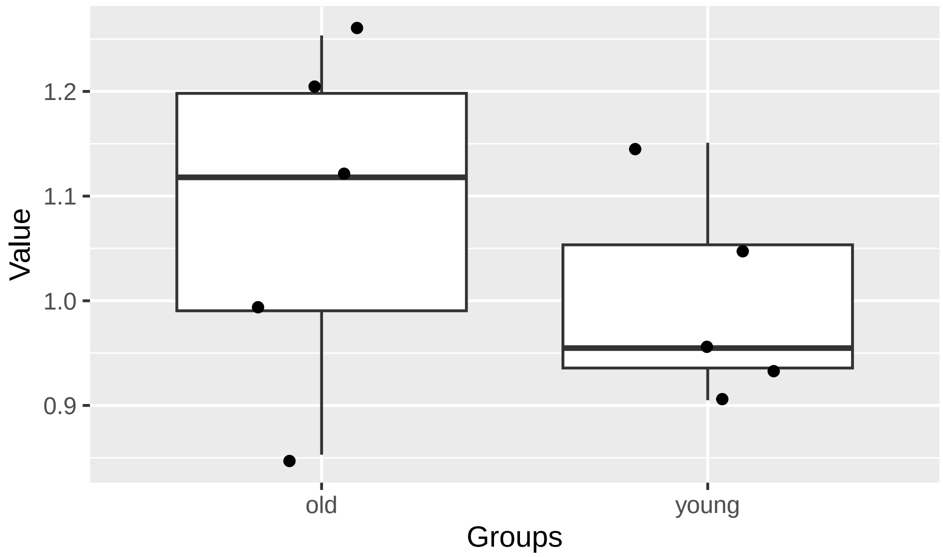


```
##
##  Welch Two Sample t-test
##
## data:  value by group
## t = 0.96696, df = 6.7023, p-value = 0.3672
## alternative hypothesis: true difference in means between group old and group young is not equal to 0
## 95 percent confidence interval:
##  -0.1212025  0.2863712
## sample estimates:
##   mean in group old mean in group young
##            1.082584            1.000000
```

```
## The result of t-test:
##  p = 0.367
##  A statistically significant difference do not exist between groups
```

Fig1_E.Western Blot_OPA1 Data analysis

Data structure

```
str(d1)
```

```
## 'data.frame':    10 obs. of  3 variables:
##  $ subject: int  1 2 3 4 5 6 7 8 9 10
##  $ group  : chr  "young" "young" "young" "young" ...
##  $ OPA1   : num  0.941 0.935 1.066 0.923 1.135 ...
```

Explorative data analysis with graphics

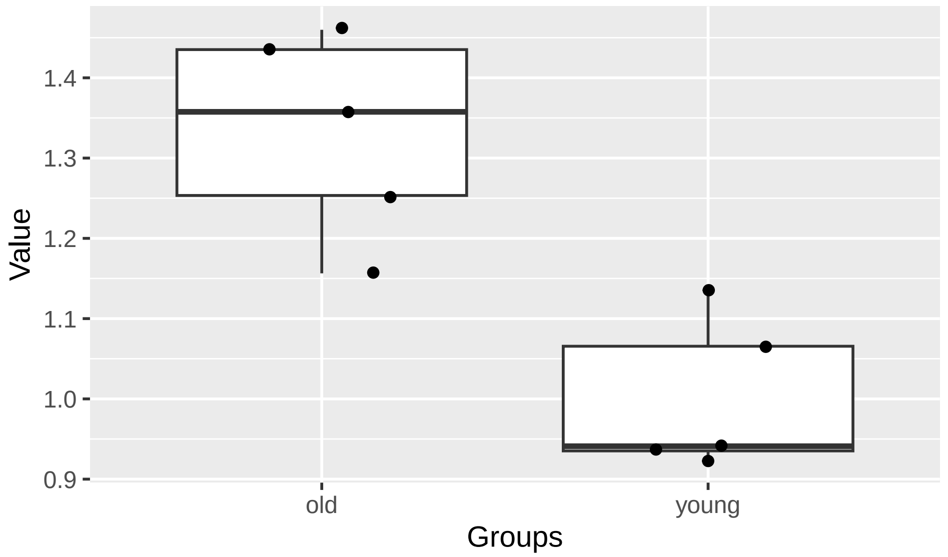


```
##
##  Welch Two Sample t-test
##
## data:  value by group
## t = 4.6848, df = 7.4054, p-value = 0.001934
## alternative hypothesis: true difference in means between group old and group young is not equal to 0
## 95 percent confidence interval:
##  0.1665029 0.4984414
## sample estimates:
##   mean in group old mean in group young
##            1.332472            1.000000
```

```
## The result of t-test:
##  p = 0.002
##  A statistically significant difference exist between groups
```

Fig1_E.Western Blot_DRP1 Data analysis

Data structure

```
str(d1)
```

```
## 'data.frame':    10 obs. of  3 variables:
##  $ subject: int  1 2 3 4 5 6 7 8 9 10
##  $ group  : chr  "young" "young" "young" "young" ...
##  $ DRP1   : num  1.152 1.197 0.961 0.847 0.843 ...
```

Explorative data analysis with graphics

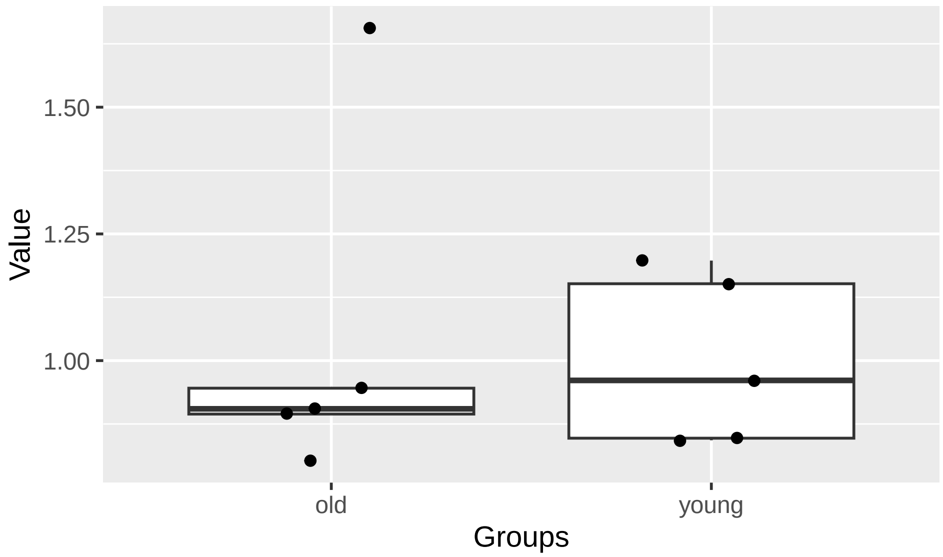


```
##
##  Welch Two Sample t-test
##
## data:  value by group
## t = 0.23546, df = 5.7486, p-value = 0.822
## alternative hypothesis: true difference in means between group old and group young is not equal to 0
## 95 percent confidence interval:
##  -0.3863733  0.4676892
## sample estimates:
##   mean in group old mean in group young
##            1.040658            1.000000
```

```
## The result of t-test:
##  p = 0.822
##  A statistically significant difference do not exist between groups
```

Fig1_E.Western Blot_MFN2 Data analysis

Data structure

```
str(d1)
```

```
## 'data.frame':    10 obs. of  3 variables:
##  $ subject: int  1 2 3 4 5 6 7 8 9 10
##  $ group  : chr  "young" "young" "young" "young" ...
##  $ MFN2   : num  0.626 1.078 1.062 1.031 1.203 ...
```

Explorative data analysis with graphics

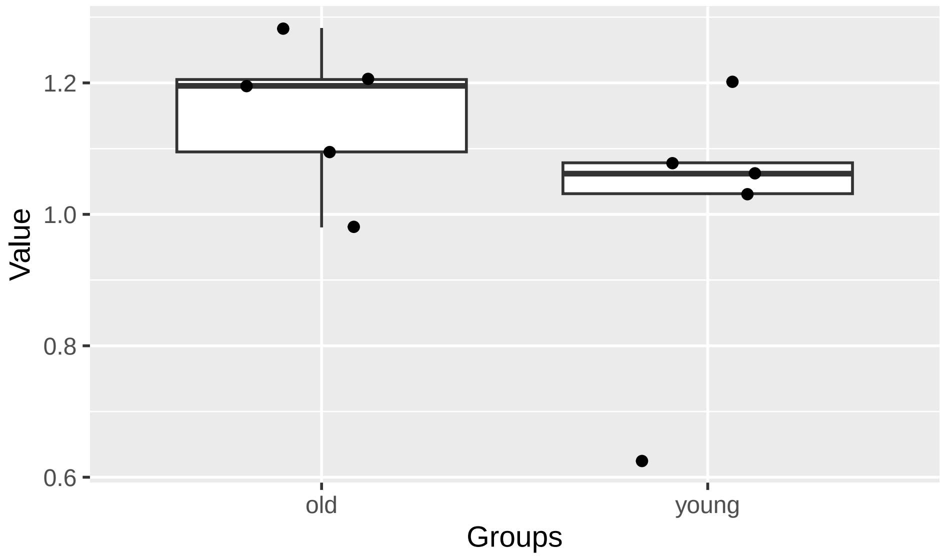


```
##
##  Welch Two Sample t-test
##
## data:  value by group
## t = 1.3666, df = 6.1101, p-value = 0.2199
## alternative hypothesis: true difference in means between group old and group young is not equal to 0
## 95 percent confidence interval:
##  -0.1188395  0.4225167
## sample estimates:
##   mean in group old mean in group young
##            1.151839            1.000000
```

```
## The result of t-test:
##  p = 0.22
##  A statistically significant difference do not exist between groups
```

Fig1_E.Western Blot_MFN1 Data analysis

Data structure

```
str(d1)
```

```
## 'data.frame':    10 obs. of  3 variables:
##  $ subject: int  1 2 3 4 5 6 7 8 9 10
##  $ group  : chr  "young" "young" "young" "young" ...
##  $ MFN1   : num  1.035 0.99 0.911 0.965 1.1 ...
```

Explorative data analysis with graphics

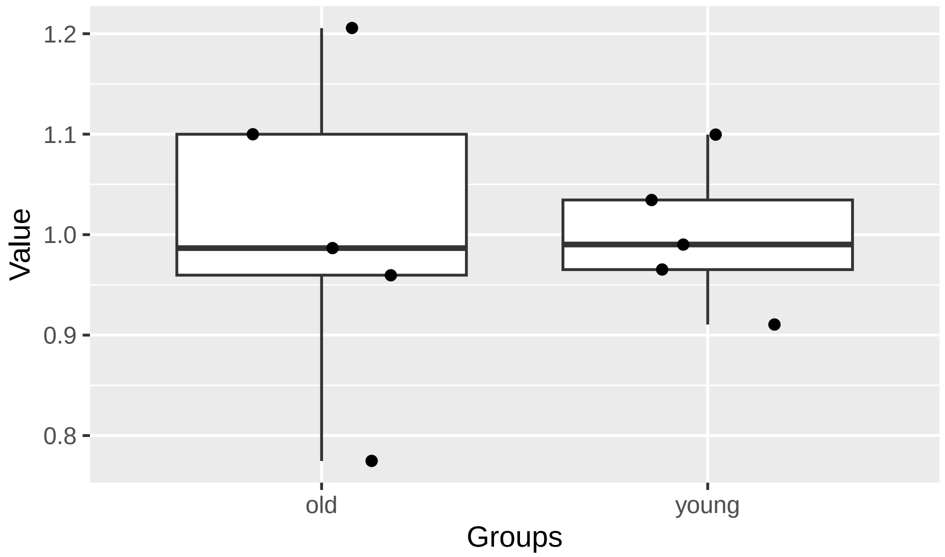


```
##
##  Welch Two Sample t-test
##
## data:  value by group
## t = 0.066959, df = 5.5024, p-value = 0.949
## alternative hypothesis: true difference in means between group old and group young is not equal to 0
## 95 percent confidence interval:
##  -0.1925198  0.2031096
## sample estimates:
##   mean in group old mean in group young
##            1.005295            1.000000
```

```
## The result of t-test:
##  p = 0.949
##  A statistically significant difference do not exist between groups
```

Figure2 Statistics

Fig2_B.LPS post24hr_OCR_old_Basal Data analysis

Data structure

```
str(d1)
```

```
## 'data.frame':    9 obs. of  4 variables:
##  $ subject: int  1 2 3 4 5 6 7 8 9
##  $ group  : chr  "con" "con" "con" "con" ...
##  $ OCR    : num  167 160 207 201 210 ...
##  $ X      : logi  NA NA NA NA NA NA ...
```

Explorative data analysis with graphics

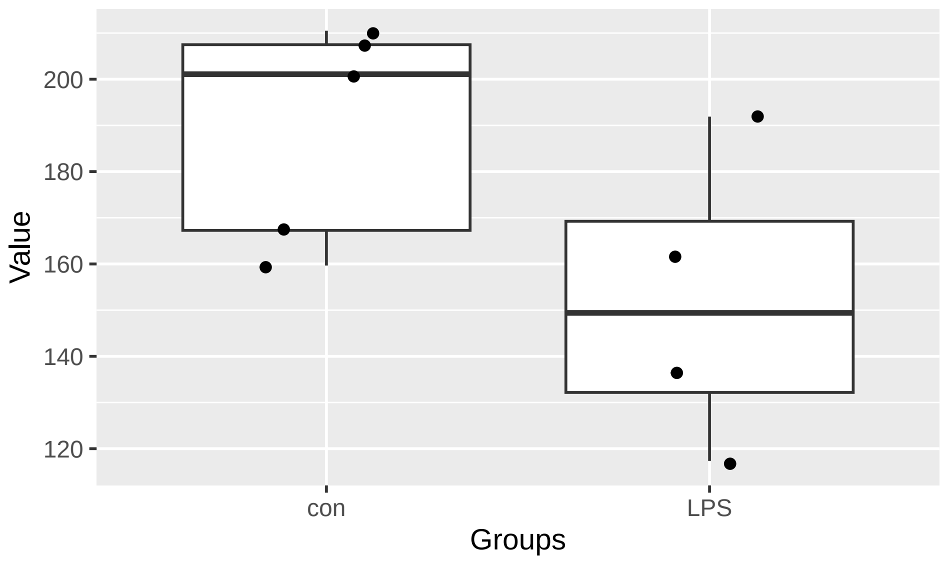


```
##
##  Welch Two Sample t-test
##
## data:  value by group
## t = 1.9254, df = 5.4358, p-value = 0.1075
## alternative hypothesis: true difference in means between group con and group LPS is not equal to 0
## 95 percent confidence interval:
##  -11.28982  85.67202
## sample estimates:
## mean in group con mean in group LPS
##          189.2001          152.0090
```

```
## The result of t-test:
##  p = 0.108
##  A statistically significant difference do not exist between groups
```

Fig2_B.LPS post24hr_OCR_old_State3 Data analysis

Data structure

```
str(d1)
```

```
## 'data.frame':    9 obs. of  4 variables:
##  $ subject: int  1 2 3 4 5 6 7 8 9
##  $ group  : chr  "con" "con" "con" "con" ...
##  $ OCR    : num  608 523 576 784 708 ...
##  $ X      : logi  NA NA NA NA NA NA ...
```

Explorative data analysis with graphics

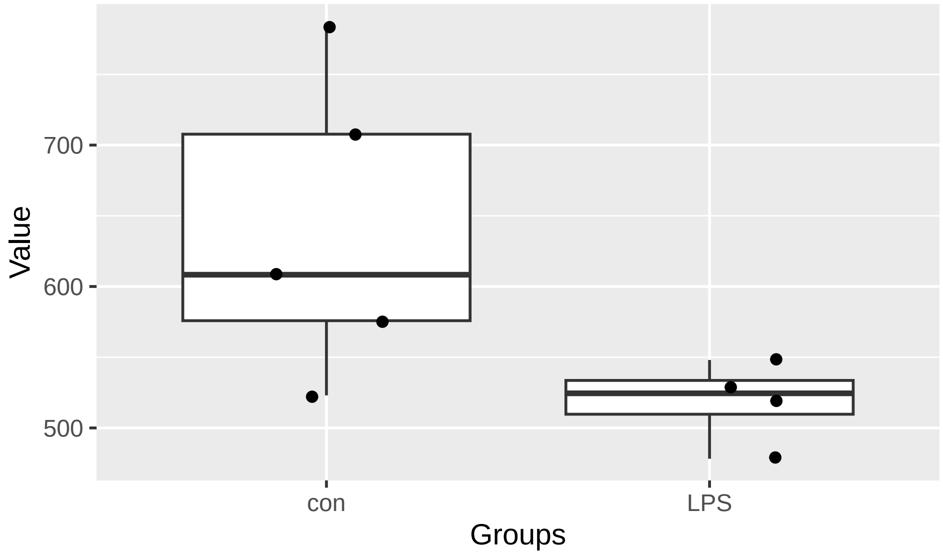


```
##
##  Welch Two Sample t-test
##
## data:  value by group
## t = 2.4565, df = 4.7626, p-value = 0.05994
## alternative hypothesis: true difference in means between group con and group LPS is not equal to 0
## 95 percent confidence interval:
##   -7.54393 249.64847
## sample estimates:
## mean in group con mean in group LPS
##          639.8603          518.8080
```

```
## The result of t-test:
##  p = 0.06
##  A statistically significant difference do not exist between groups
```

Fig2_B.LPS post24hr_OCR_old_State4o Data analysis

Data structure

```
str(d1)
```

```
## 'data.frame':    9 obs. of  4 variables:
##  $ subject: int  1 2 3 4 5 6 7 8 9
##  $ group  : chr  "con" "con" "con" "con" ...
##  $ OCR    : num  133 120 143 180 147 ...
##  $ X      : logi  NA NA NA NA NA NA ...
```

Explorative data analysis with graphics

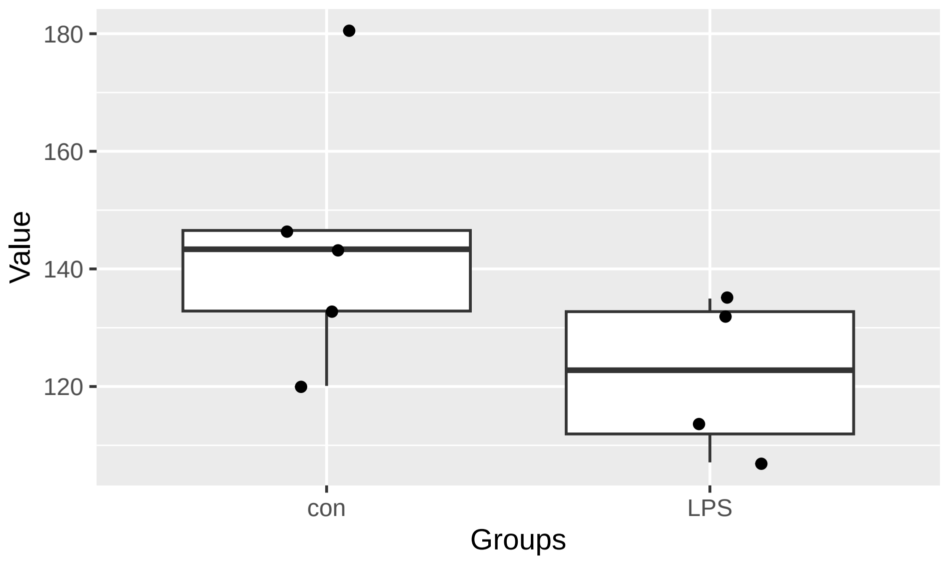


```
##
##  Welch Two Sample t-test
##
## data:  value by group
## t = 1.8694, df = 6.6525, p-value = 0.106
## alternative hypothesis: true difference in means between group con and group LPS is not equal to 0
## 95 percent confidence interval:
##  -6.336678 51.851024
## sample estimates:
## mean in group con mean in group LPS
##          144.6554          121.8983
```

```
## The result of t-test:
##  p = 0.106
##  A statistically significant difference do not exist between groups
```

Fig2_B.LPS post24_OCR_old_State3u Data analysis

Data structure

```
str(d1)
```

```
## 'data.frame':    9 obs. of  4 variables:
##  $ subject: int  1 2 3 4 5 6 7 8 9
##  $ group  : chr  "con" "con" "con" "con" ...
##  $ OCR    : num  563 508 561 683 595 ...
##  $ X      : logi  NA NA NA NA NA NA ...
```

Explorative data analysis with graphics

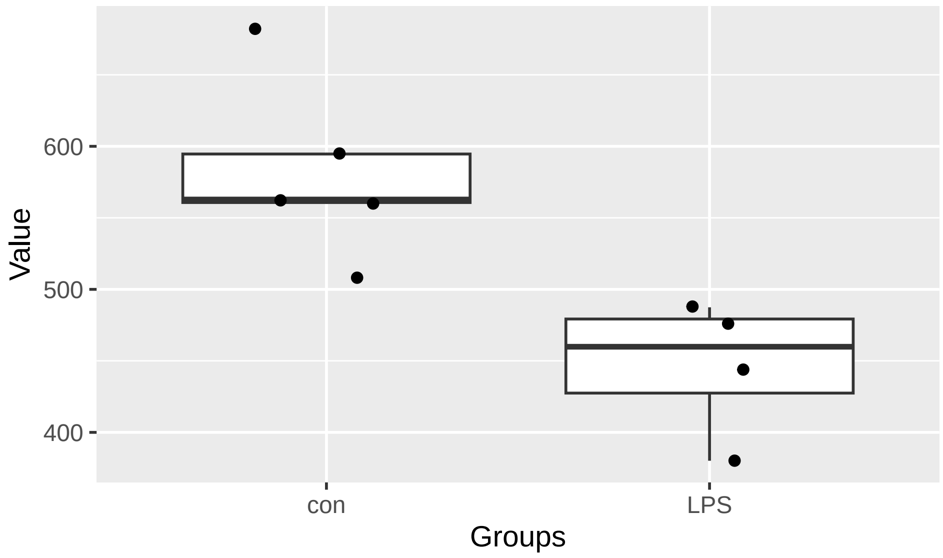


```
##
##  Welch Two Sample t-test
##
## data:  value by group
## t = 3.5871, df = 6.9918, p-value = 0.008909
## alternative hypothesis: true difference in means between group con and group LPS is not equal to 0
## 95 percent confidence interval:
##   45.96901 223.92632
## sample estimates:
## mean in group con mean in group LPS
##          581.7870          446.8393
```

```
## The result of t-test:
##  p = 0.009
##  A statistically significant difference exist between groups
```

Fig2_C.LPS post24hr_old_RCR Data analysis

Data structure

```
str(d1)
```

```
## 'data.frame':    9 obs. of  4 variables:
##  $ subject: int  1 2 3 4 5 6 7 8 9
##  $ group  : chr  "con" "con" "con" "con" ...
##  $ RCR    : num  4.58 4.35 4.02 4.35 4.83 ...
##  $ X      : logi  NA NA NA NA NA NA ...
```

Explorative data analysis with graphics

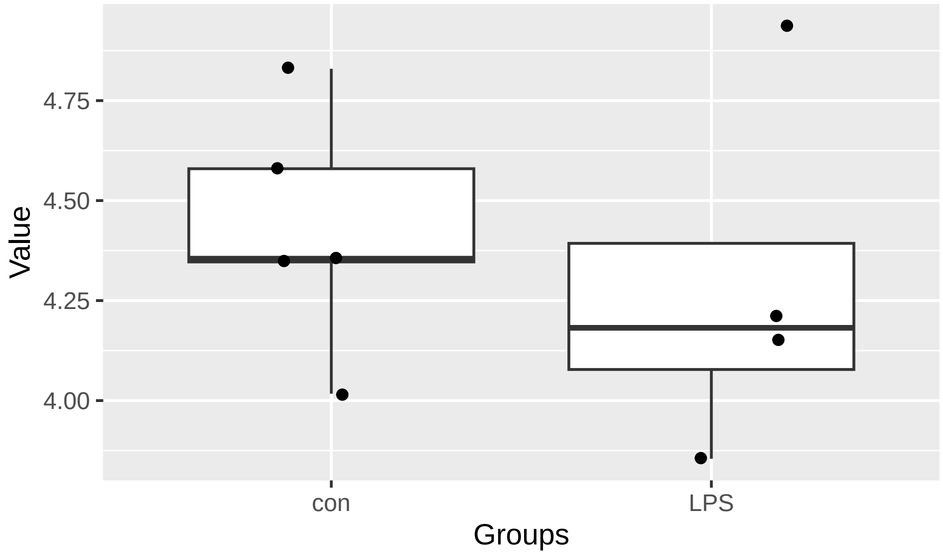


```
##
##  Welch Two Sample t-test
##
## data:  value by group
## t = 0.51289, df = 4.9858, p-value = 0.6299
## alternative hypothesis: true difference in means between group con and group LPS is not equal to 0
## 95 percent confidence interval:
##  -0.5489819  0.8223602
## sample estimates:
## mean in group con mean in group LPS
##          4.425566          4.288877
```

```
## The result of t-test:
##  p = 0.63
##  A statistically significant difference do not exist between groups
```

Fig2_C.LPS post24hr_old_RCRu Data analysis

Data structure

```
str(d1)
```

```
## 'data.frame':    9 obs. of  4 variables:
##  $ subject: int  1 2 3 4 5 6 7 8 9
##  $ group  : chr  "con" "con" "con" "con" ...
##  $ RCRu   : num  3.03 2.54 2.73 2.89 2.92 ...
##  $ X      : logi  NA NA NA NA NA NA ...
```

Explorative data analysis with graphics

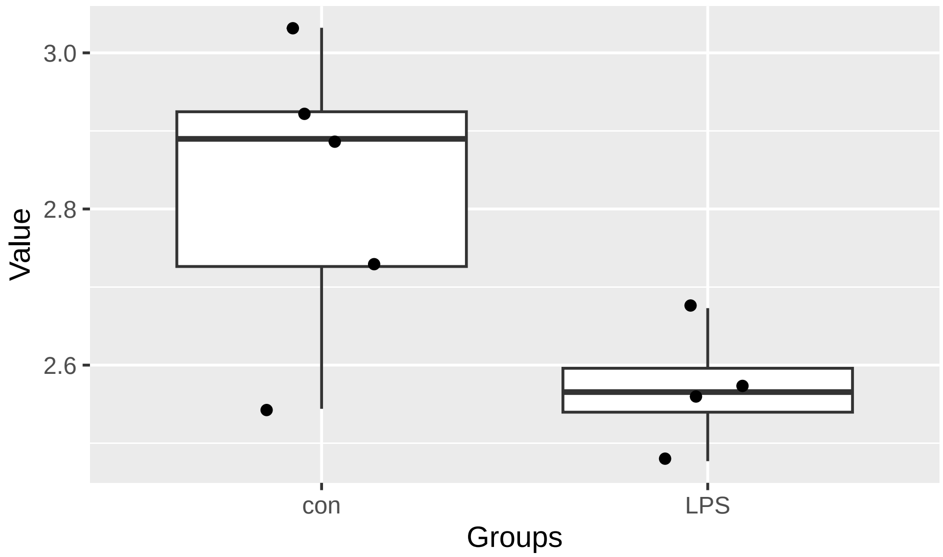


```
##
##  Welch Two Sample t-test
##
## data:  value by group
## t = 2.6842, df = 5.601, p-value = 0.03889
## alternative hypothesis: true difference in means between group con and group LPS is not equal to 0
## 95 percent confidence interval:
##  0.01833579 0.48798212
## sample estimates:
## mean in group con mean in group LPS
##          2.823385          2.570226
```

```
## The result of t-test:
##  p = 0.039
##  A statistically significant difference does not exist between groups
```

Fig2_E.LPS post24hr_OCR_young_Basal Data analysis

Data structure

```
str(d1)
```

```
## 'data.frame':    10 obs. of  4 variables:
##  $ subject: int  1 2 3 4 5 6 7 8 9 10
##  $ group  : chr  "con" "con" "con" "con" ...
##  $ OCR    : num  277 301 374 342 438 ...
##  $ X      : logi  NA NA NA NA NA NA ...
```

Explorative data analysis with graphics

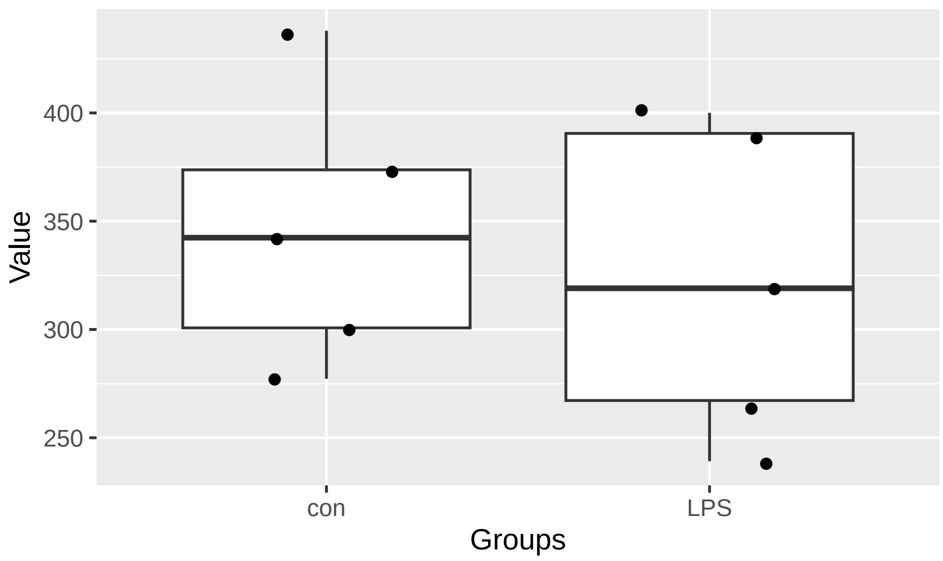


```
##
##  Welch Two Sample t-test
##
## data:  value by group
## t = 0.54216, df = 7.8739, p-value = 0.6027
## alternative hypothesis: true difference in means between group con and group LPS is not equal to 0
## 95 percent confidence interval:
##  -75.79161 122.21432
## sample estimates:
## mean in group con mean in group LPS
##          346.4033          323.1919
```

```
## The result of t-test:
##  p = 0.603
##  A statistically significant difference do not exist between groups
```

Fig2_E.LPS post24hr_OCR_young_State3 Data analysis

Data structure

```
str(d1)
```

```
## 'data.frame':    10 obs. of  3 variables:
##  $ subject: int  1 2 3 4 5 6 7 8 9 10
##  $ group  : chr  "con" "con" "con" "con" ...
##  $ OCR    : num  801 1010 1201 1106 1080 ...
```

Explorative data analysis with graphics

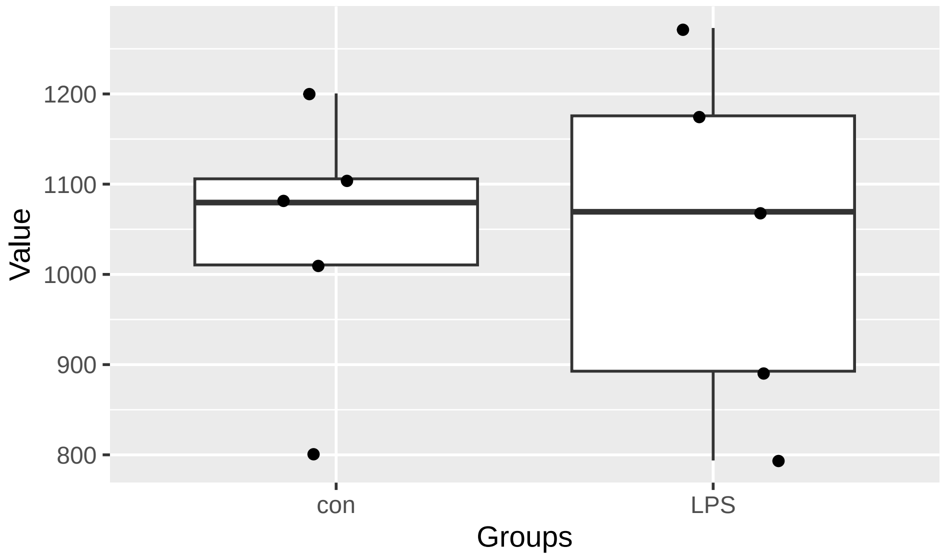


```
##
##  Welch Two Sample t-test
##
## data:  value by group
## t = -0.012396, df = 7.4552, p-value = 0.9904
## alternative hypothesis: true difference in means between group con and group LPS is not equal to 0
## 95 percent confidence interval:
##  -260.1542  257.4074
## sample estimates:
## mean in group con mean in group LPS
##          1039.559          1040.933
```

```
## The result of t-test:
##  p = 0.99
##  A statistically significant difference do not exist between groups
```

Fig2_E.LPS post24hr_OCR_young_State4o Data analysis

Data structure

```
str(d1)
```

```
## 'data.frame':    10 obs. of  3 variables:
##  $ subject: int  1 2 3 4 5 6 7 8 9 10
##  $ group  : chr  "con" "con" "con" "con" ...
##  $ OCR    : num  199 208 255 228 262 ...
```

Explorative data analysis with graphics

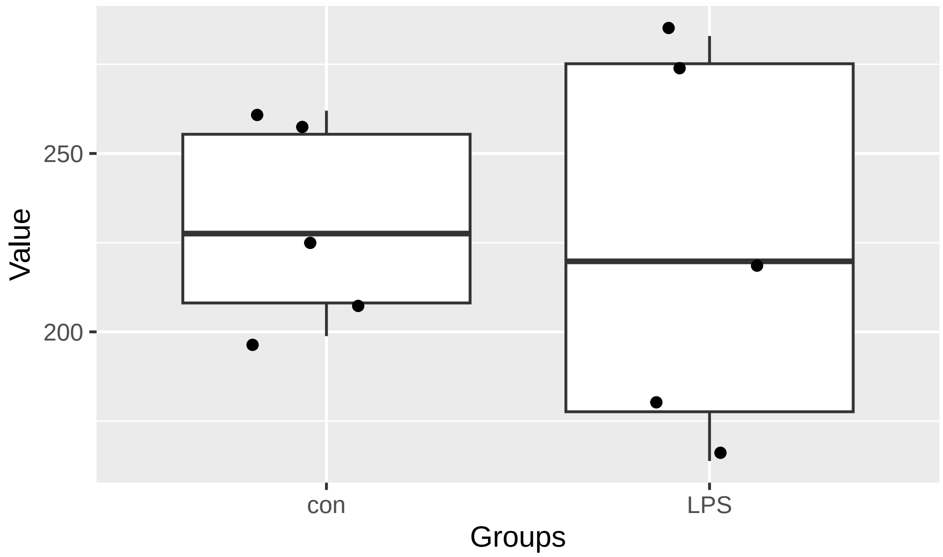


```
##
##  Welch Two Sample t-test
##
## data:  value by group
## t = 0.23773, df = 5.9684, p-value = 0.82
## alternative hypothesis: true difference in means between group con and group LPS is not equal to 0
## 95 percent confidence interval:
##  -60.61325  73.64014
## sample estimates:
## mean in group con mean in group LPS
##          230.3629          223.8495
```

```
## The result of t-test:
##  p = 0.82
##  A statistically significant difference do not exist between groups
```

Fig2_E.LPS post24hr_OCR_young_State3u Data analysis

Data structure

```
str(d1)
```

```
## 'data.frame':    10 obs. of  3 variables:
##  $ subject: int  1 2 3 4 5 6 7 8 9 10
##  $ group  : chr  "con" "con" "con" "con" ...
##  $ OCR    : num  669 972 1069 943 1058 ...
```

Explorative data analysis with graphics

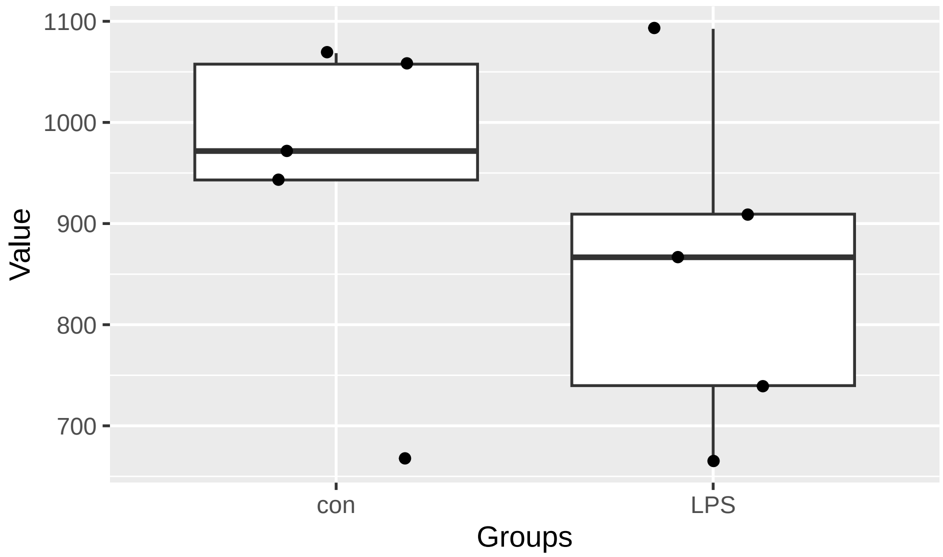


```
##
##  Welch Two Sample t-test
##
## data:  value by group
## t = 0.8429, df = 7.9978, p-value = 0.4238
## alternative hypothesis: true difference in means between group con and group LPS is not equal to 0
## 95 percent confidence interval:
##  -151.1625  325.3185
## sample estimates:
## mean in group con mean in group LPS
##          941.9361          854.8581
```

```
## The result of t-test:
##  p = 0.424
##  A statistically significant difference do not exist between groups
```

Fig2_F.LPS post24hr_young_RCR Data analysis

Data structure

```
str(d1)
```

```
## 'data.frame':    10 obs. of  3 variables:
##  $ subject: int  1 2 3 4 5 6 7 8 9 10
##  $ group  : chr  "con" "con" "con" "con" ...
##  $ RCR    : num  2.97 3.49 3.34 3.44 3.1 ...
```

Explorative data analysis with graphics

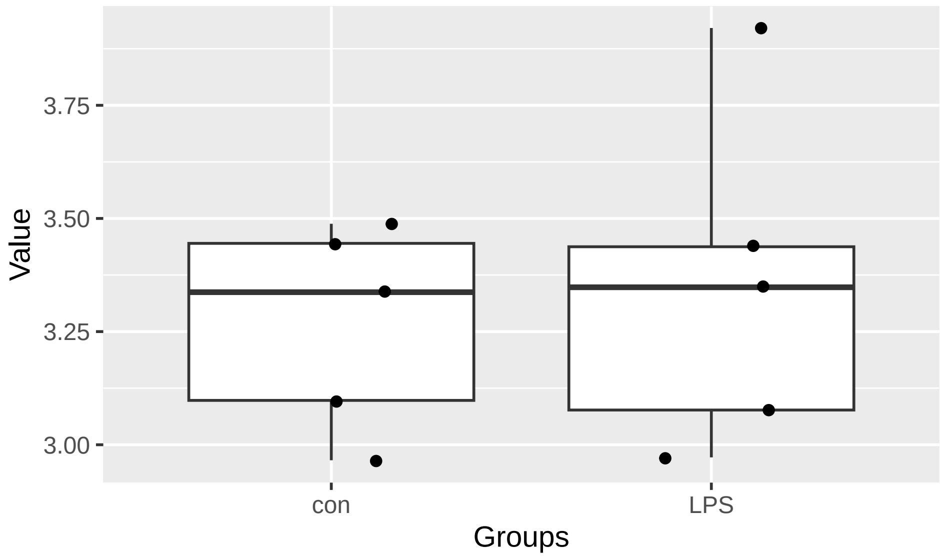


```
##
##  Welch Two Sample t-test
##
## data:  value by group
## t = -0.43315, df = 6.6133, p-value = 0.6787
## alternative hypothesis: true difference in means between group con and group LPS is not equal to 0
## 95 percent confidence interval:
##  -0.5493834  0.3809771
## sample estimates:
## mean in group con mean in group LPS
##          3.266804          3.351007
```

```
## The result of t-test:
##  p = 0.679
##  A statistically significant difference do not exist between groups
```

Fig2_F.LPS post24hr_young_RCRu Data analysis

Data structure

```
str(d1)
```

```
## 'data.frame':    10 obs. of  3 variables:
##  $ subject: int  1 2 3 4 5 6 7 8 9 10
##  $ group  : chr  "con" "con" "con" "con" ...
##  $ RCRu   : num  2.53 3.37 3.01 2.99 3.04 ...
```

Explorative data analysis with graphics

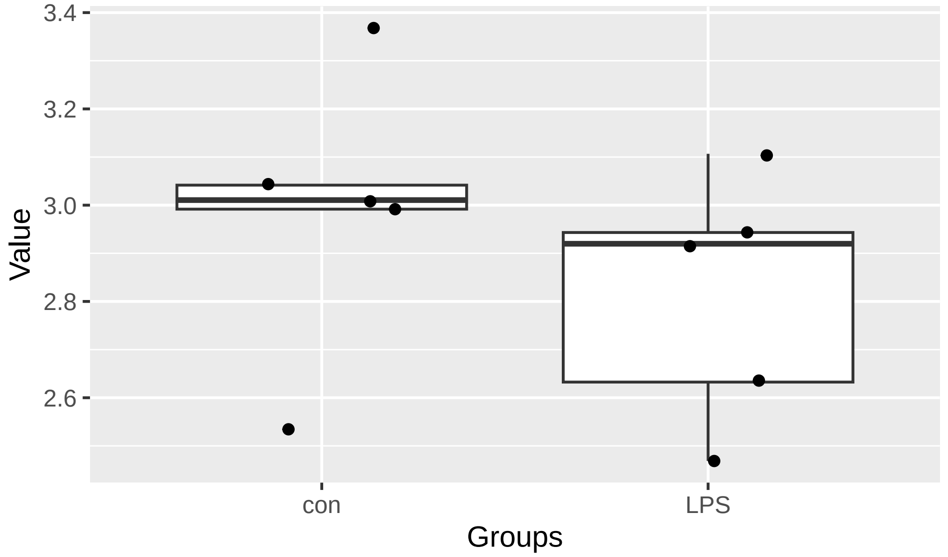


```
##
##  Welch Two Sample t-test
##
## data:  value by group
## t = 0.99281, df = 7.8393, p-value = 0.3505
## alternative hypothesis: true difference in means between group con and group LPS is not equal to 0
## 95 percent confidence interval:
##  -0.2327196  0.5824039
## sample estimates:
## mean in group con mean in group LPS
##          2.989065          2.814222
```

```
## The result of t-test:
##  p = 0.35
##  A statistically significant difference do not exist between groups
```

Fig2_G.LPS post6hr_qPCR_old_TNFalpah_Data analysis

Boohwi Hong

```
## Present data is ** LPS post6hr_qPCR_old_TNFalpah.csv **
##
## **  Data structure  **
## 'data.frame':    10 obs. of  3 variables:
##  $ subject : int  1 2 3 4 5 6 7 8 9 10
##  $ group   : chr  "con" "con" "con" "con" ...
##  $ TNFalpha: num  0.34 0.396 0.639 NA NA ...
##
## ** Explorative data analysis with graphics**

## Warning: Removed 2 rows containing non-finite values (`stat_boxplot()`).

## Warning: Removed 2 rows containing missing values (`geom_point()`).
```

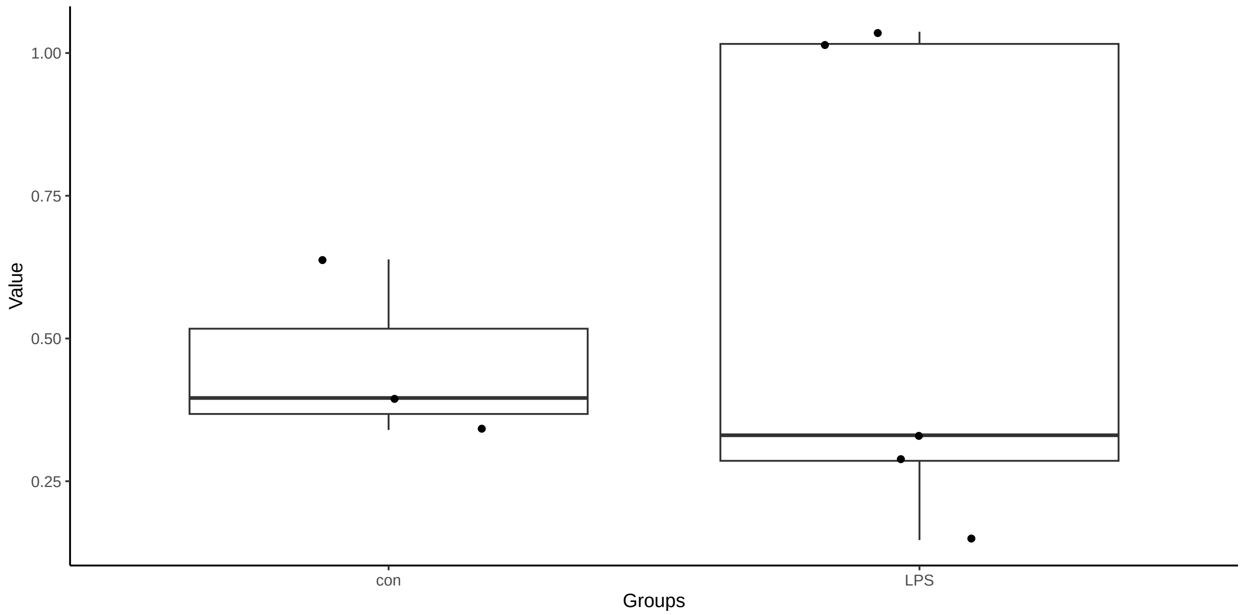


```
## 1. Normality assumption test by Shapiro_Wilk test is
##  p = 0.358
##  Normality assumption was not rejected
## 2. Equal variance test by Bartlett test is
##  p = 0.203
##  Equal variance assumption was not rejected
## 3. The result of anova is
##  p = 0.704
##  A statistically significant difference do not exist between groups
##
```

Fig2_G.LPS post6hr_qPCR_old_IL-1beta_Data analysis

Boohwi Hong

```
## Present data is ** LPS post6hr_qPCR_old_IL-1beta.csv **
##
## **  Data structure  **
## 'data.frame':    8 obs. of  3 variables:
##  $ subject : int  1 2 3 4 5 6 7 8
##  $ group   : chr  "con" "con" "con" "LPS" ...
##  $ IL.1beta: num  0.72 0.799 1.481 2.406 2.138 ...
##
## ** Explorative data analysis with graphics**
```

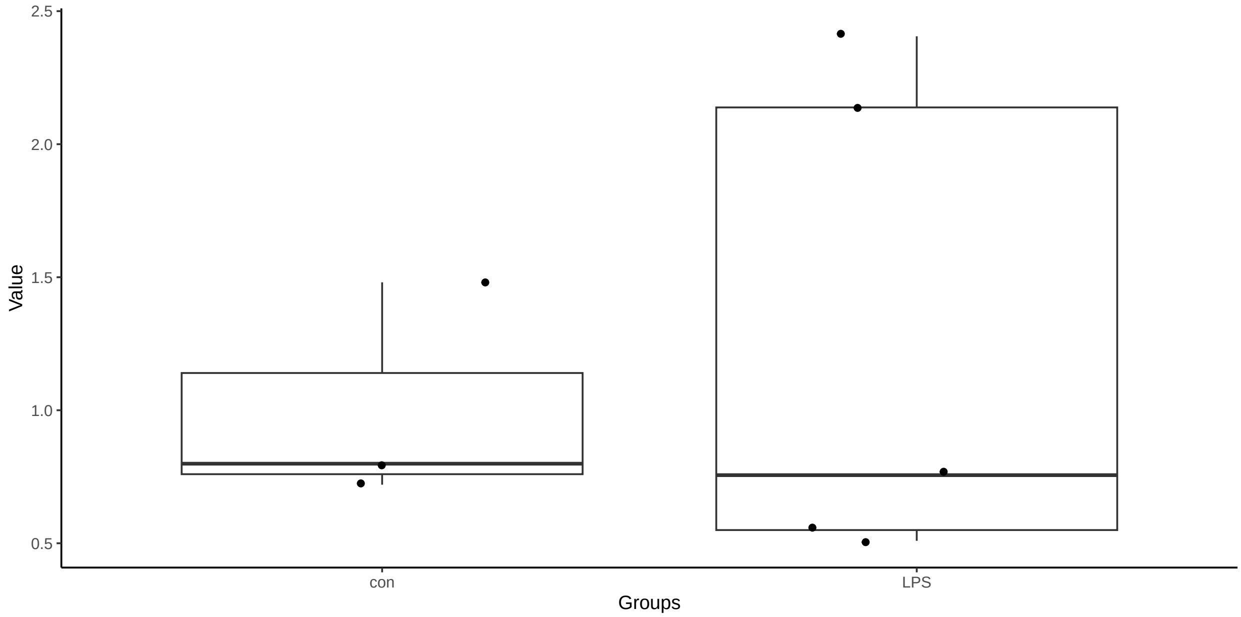


```
## 1. Normality assumption test by Shapiro_Wilk test is
##  p = 0.238
##  Normality assumption was not rejected
## 2. Equal variance test by Bartlett test is
##  p = 0.293
##  Equal variance assumption was not rejected
## 3. The result of anova is
##  p = 0.655
##  A statistically significant difference do not exist between groups
##
```

Fig2_G.LPS post6hr_qPCR_old_IL-10_Data analysis

BooHwi Hong

```
## Present data is ** LPS post6hr_qPCR_old_IL-10.csv **
##
## **  Data structure  **
## 'data.frame':    8 obs. of  3 variables:
##  $ subject: int  1 2 3 4 5 6 7 8
##  $ group  : chr  "con" "con" "con" "LPS" ...
##  $ IL.10  : num  0.661 0.903 1.436 2.873 3.661 ...
##
## ** Explorative data analysis with graphics**
```

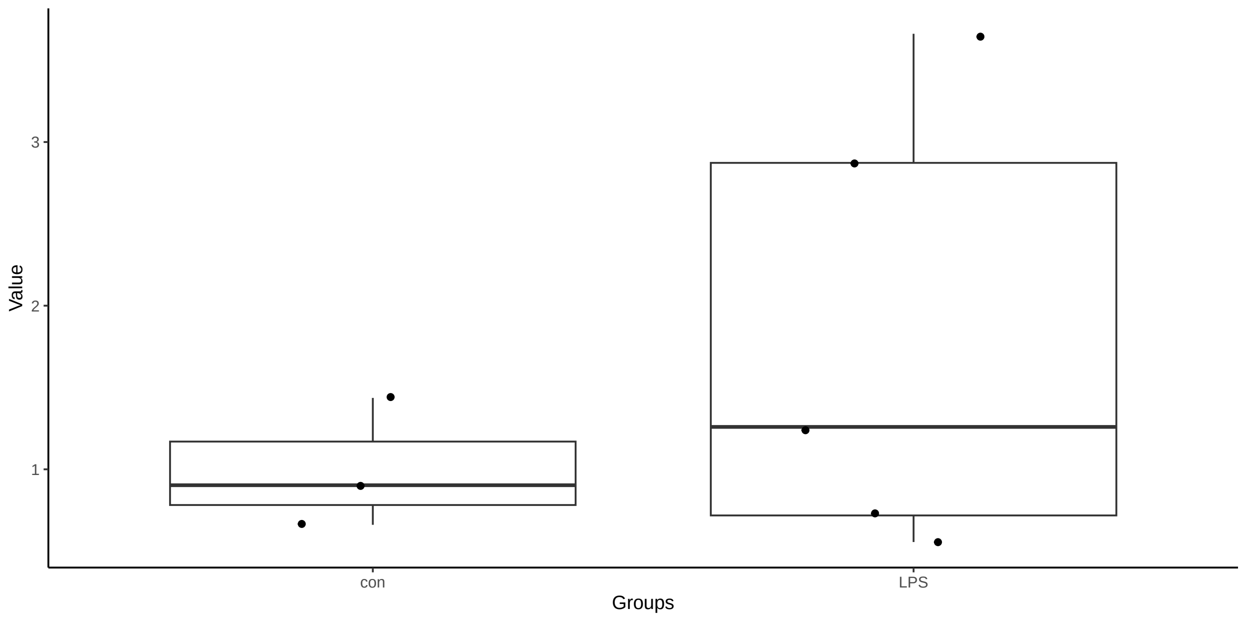


```
## 1. Normality assumption test by Shapiro_Wilk test is
##  p = 0.72
##  Normality assumption was not rejected
## 2. Equal variance test by Bartlett test is
##  p = 0.126
##  Equal variance assumption was not rejected
## 3. The result of anova is
##  p = 0.370
##  A statistically significant difference do not exist between groups
##
```

Fig2_G.LPS post6hr_qPCR_old_IL-6_Data analysis

Boohwi Hong

```
## Present data is ** LPS post6hr_qPCR_old_IL-6.csv **
##
## **  Data structure  **
## 'data.frame':    8 obs. of  3 variables:
##  $ subject: int  1 2 3 4 5 6 7 8
##  $ group  : chr  "con" "con" "con" "LPS" ...
##  $ IL.6   : num  0.531 0.756 1.713 2.474 0.565 ...
##
## ** Explorative data analysis with graphics**
```

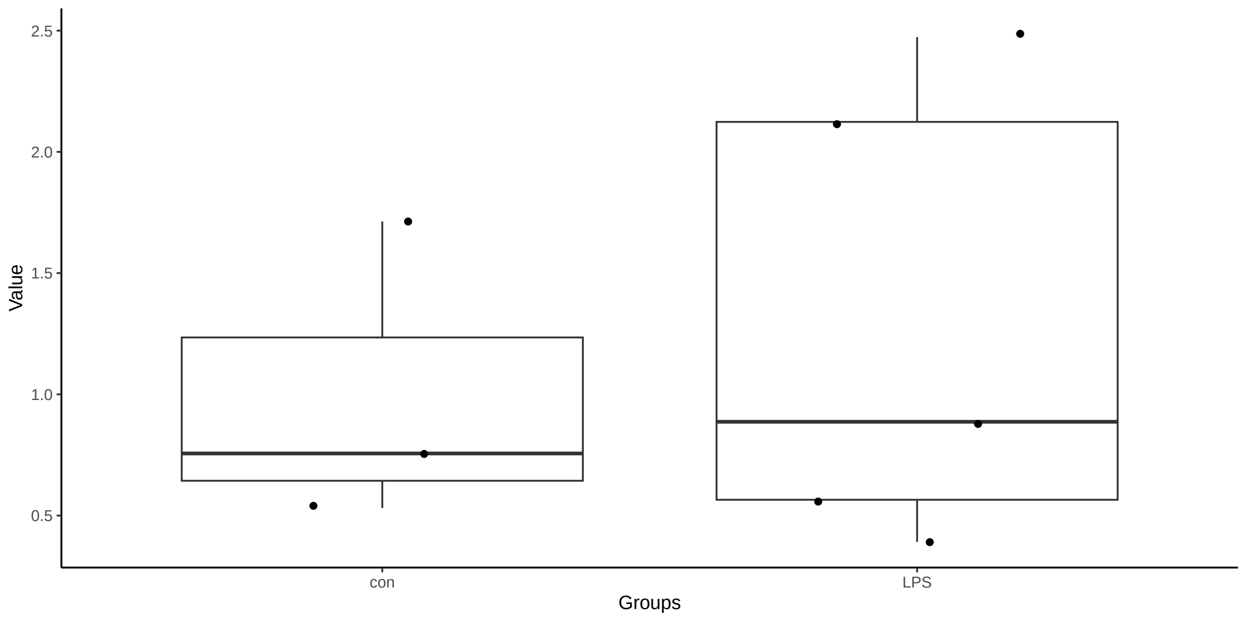


```
## 1. Normality assumption test by Shapiro_Wilk test is
##  p = 0.21
##  Normality assumption was not rejected
## 2. Equal variance test by Bartlett test is
##  p = 0.560
##  Equal variance assumption was not rejected
## 3. The result of anova is
##  p = 0.661
##  A statistically significant difference do not exist between groups
##
```

Fig2_H.LPS post24hr_qPCR_old_TNFalpha Data analysis

Data structure

```
str(d1)
```

```
## 'data.frame':    9 obs. of  3 variables:
##  $ subject : int  1 2 3 4 5 6 7 8 9
##  $ group   : chr  "con" "con" "con" "con" ...
##  $ TNFalpha: num  0.985 0.631 1.074 1.31 1.123 ...
```

Explorative data analysis with graphics

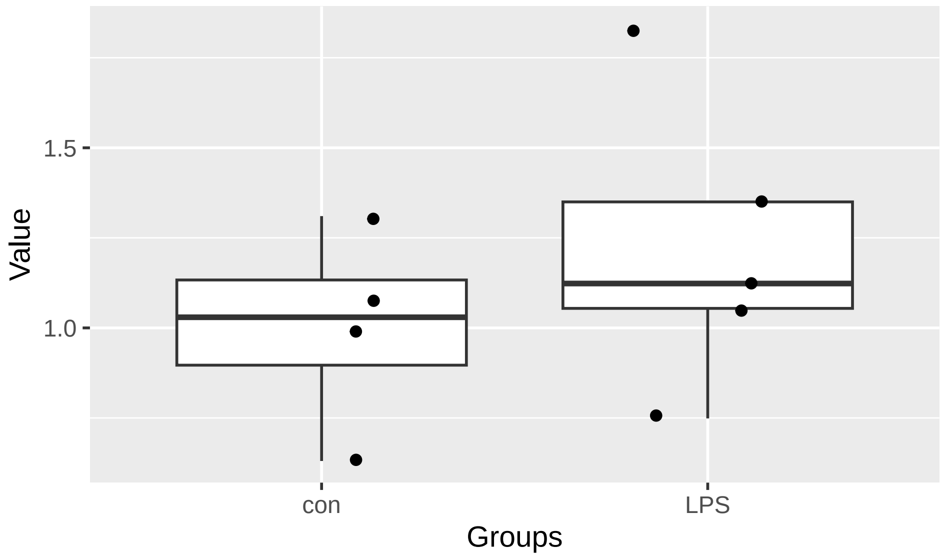


```
##
##  Welch Two Sample t-test
##
## data:  value by group
## t = -0.9682, df = 6.9308, p-value = 0.3655
## alternative hypothesis: true difference in means between group con and group LPS is not equal to 0
## 95 percent confidence interval:
##  -0.7641655  0.3208164
## sample estimates:
## mean in group con mean in group LPS
##          1.000000          1.221675
```

```
## The result of t-test:
##  p = 0.366
##  A statistically significant difference do not exist between groups
```

Fig2_H.LPS post24hr_qPCR_old_IL-1beta Data analysis

Data structure

```
str(d1)
```

```
## 'data.frame':    9 obs. of  3 variables:
##  $ subject : int  1 2 3 4 5 6 7 8 9
##  $ group   : chr  "con" "con" "con" "con" ...
##  $ IL.1beta: num  0.87 0.609 1.183 1.339 1.13 ...
```

Explorative data analysis with graphics

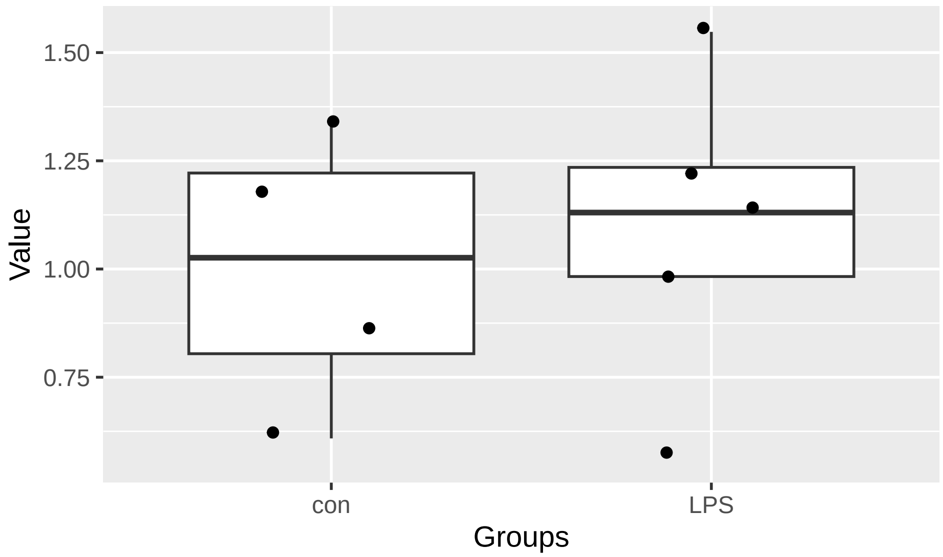


```
##
##  Welch Two Sample t-test
##
## data:  value by group
## t = -0.39298, df = 6.8525, p-value = 0.7063
## alternative hypothesis: true difference in means between group con and group LPS is not equal to 0
## 95 percent confidence interval:
##  -0.6369758  0.4561058
## sample estimates:
## mean in group con mean in group LPS
##          1.000000          1.090435
```

```
## The result of t-test:
##  p = 0.706
##  A statistically significant difference do not exist between groups
```

Fig2_H.LPS post24hr_qPCR_old_IL-10 Data analysis

Data structure

```
str(d1)
```

```
## 'data.frame':    9 obs. of  3 variables:
##  $ subject: int  1 2 3 4 5 6 7 8 9
##  $ group  : chr  "con" "con" "con" "con" ...
##  $ IL.10  : num  1.044 0.679 1.17 1.107 0.867 ...
```

Explorative data analysis with graphics

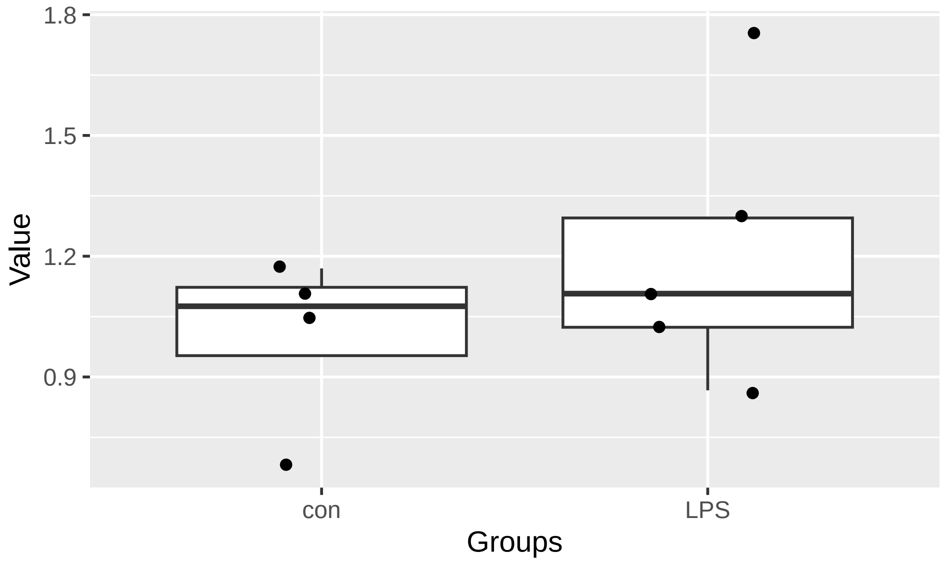


```
##
##  Welch Two Sample t-test
##
## data:  value by group
## t = -1.112, df = 6.7904, p-value = 0.304
## alternative hypothesis: true difference in means between group con and group LPS is not equal to 0
## 95 percent confidence interval:
##  -0.6574816  0.2386824
## sample estimates:
## mean in group con mean in group LPS
##            1.0000            1.2094
```

```
## The result of t-test:
##  p = 0.304
##  A statistically significant difference do not exist between groups
```

Fig2_**H**.LPS post24hr_qPCR_old_IL-6 Data analysis

Data structure

```
str(d1)
```

```
## 'data.frame':    9 obs. of  3 variables:
##  $ subject: int  1 2 3 4 5 6 7 8 9
##  $ group  : chr  "con" "con" "con" "con" ...
##  $ IL.6   : num  0.922 0.599 1.263 1.217 1.041 ...
```

Explorative data analysis with graphics

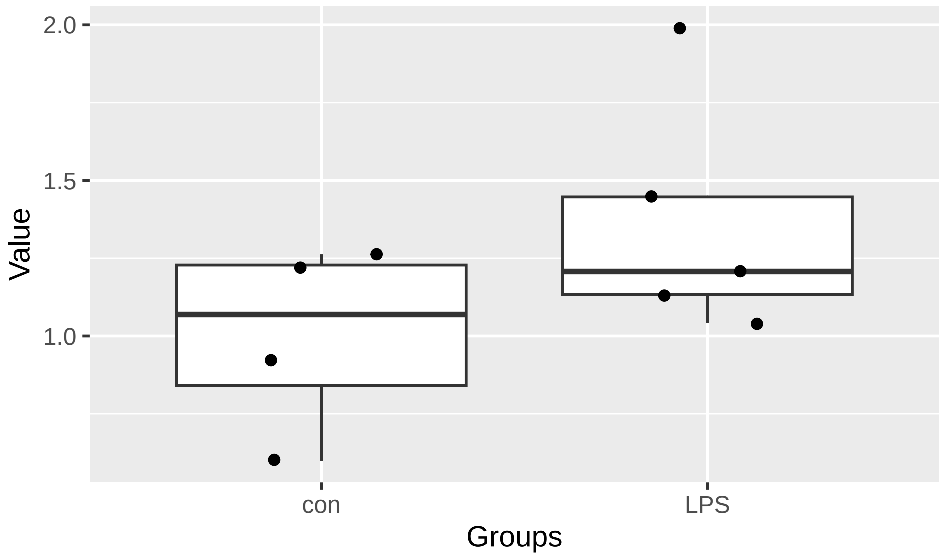


```
##
##  Welch Two Sample t-test
##
## data:  value by group
## t = -1.5868, df = 6.9895, p-value = 0.1566
## alternative hypothesis: true difference in means between group con and group LPS is not equal to 0
## 95 percent confidence interval:
##  -0.9067129  0.1786021
## sample estimates:
## mean in group con mean in group LPS
##          1.000000          1.364055
```

```
## The result of t-test:
##  p = 0.157
##  A statistically significant difference do not exist between groups
```

Fig2_I.LPS post48hr_qPCR_old_TNFalpha Data analysis

Data structure

```
str(d1)
```

```
## 'data.frame':    10 obs. of  3 variables:
##  $ subject : int  1 2 3 4 5 6 7 8 9 10
##  $ group   : chr  "con" "con" "con" "con" ...
##  $ TNFalpha: num  1.553 0.45 1.025 0.497 1.475 ...
```

Explorative data analysis with graphics

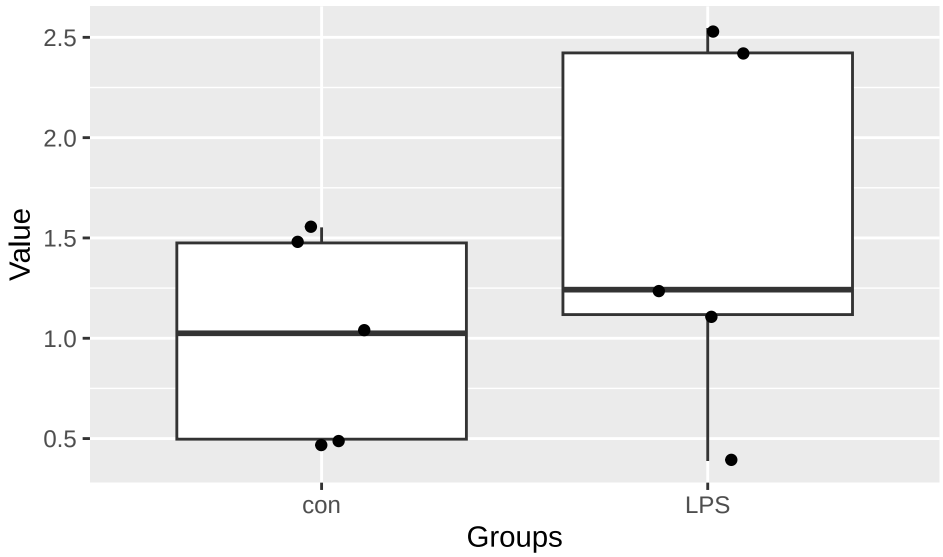


```
##
##  Welch Two Sample t-test
##
## data:  value by group
## t = -1.1493, df = 6.3292, p-value = 0.292
## alternative hypothesis: true difference in means between group con and group LPS is not equal to 0
## 95 percent confidence interval:
##  -1.6861438  0.5991874
## sample estimates:
## mean in group con mean in group LPS
##          1.000000          1.543478
```

```
## The result of t-test:
##  p = 0.292
##  A statistically significant difference do not exist between groups
```

Fig2_I.LPS post48hr_qPCR_old_IL-1beta Data analysis

Data structure

```
str(d1)
```

```
## 'data.frame':    10 obs. of  3 variables:
##  $ subject : int  1 2 3 4 5 6 7 8 9 10
##  $ group   : chr  "con" "con" "con" "con" ...
##  $ IL.1beta: num  0.882 0.855 1.526 0.776 0.961 ...
```

Explorative data analysis with graphics

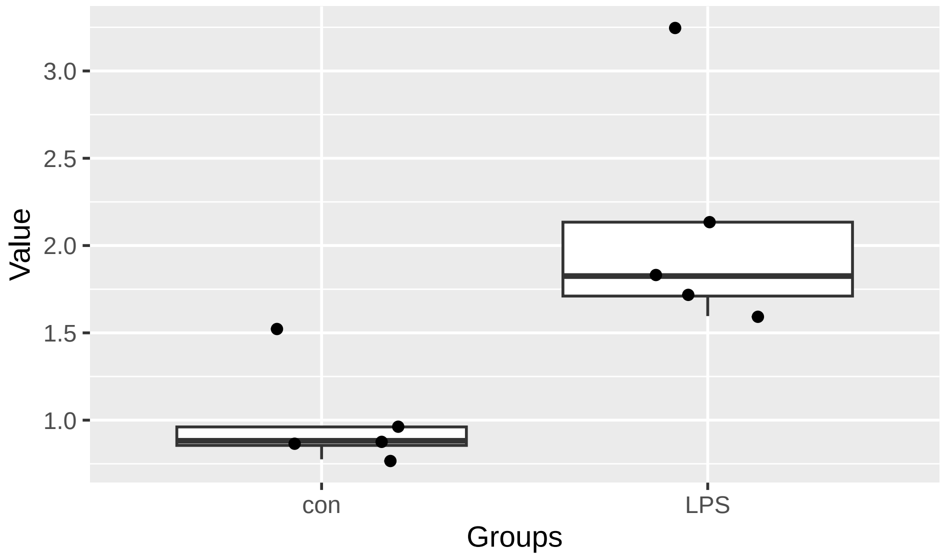


```
##
##  Welch Two Sample t-test
##
## data:  value by group
## t = -3.3676, df = 5.571, p-value = 0.0169
## alternative hypothesis: true difference in means between group con and group LPS is not equal to 0
## 95 percent confidence interval:
##  -1.9153551 -0.2857037
## sample estimates:
## mean in group con mean in group LPS
##         0.9999998         2.1005292
```

```
## The result of t-test:
##  p = 0.017
##  A statistically significant difference exist between groups
```

Fig2_I.LPS post48hr_qPCR_old_IL-10 Data analysis

Data structure

```
str(d1)
```

```
## 'data.frame':    10 obs. of  3 variables:
##  $ subject: int  1 2 3 4 5 6 7 8 9 10
##  $ group  : chr  "con" "con" "con" "con" ...
##  $ IL.10  : num  1.157 0.868 1.134 0.926 0.914 ...
```

Explorative data analysis with graphics

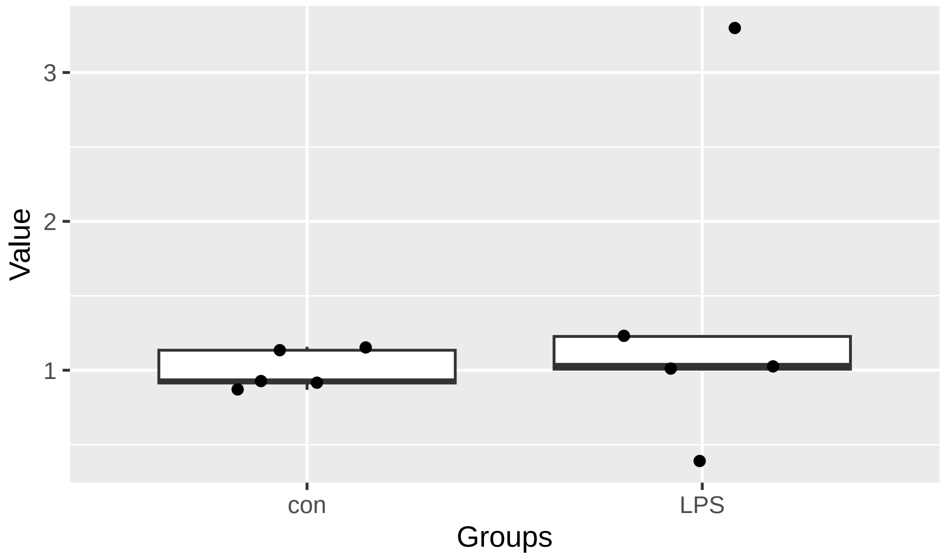


```
##
##  Welch Two Sample t-test
##
## data:  value by group
## t = -0.7815, df = 4.1183, p-value = 0.477
## alternative hypothesis: true difference in means between group con and group LPS is not equal to 0
## 95 percent confidence interval:
##  -1.7654448  0.9830372
## sample estimates:
## mean in group con mean in group LPS
##          1.000000          1.391204
```

```
## The result of t-test:
##  p = 0.477
##  A statistically significant difference do not exist between groups
```

Fig2_I.LPS post48hr_qPCR_old_IL-6 Data analysis

Data structure

```
str(d1)
```

```
## 'data.frame':    9 obs. of  3 variables:
##  $ subject: int  1 2 3 4 5 6 7 8 9
##  $ group  : chr  "con" "con" "con" "con" ...
##  $ IL.6   : num  1.333 0.64 0.947 0.733 1.347 ...
```

Explorative data analysis with graphics

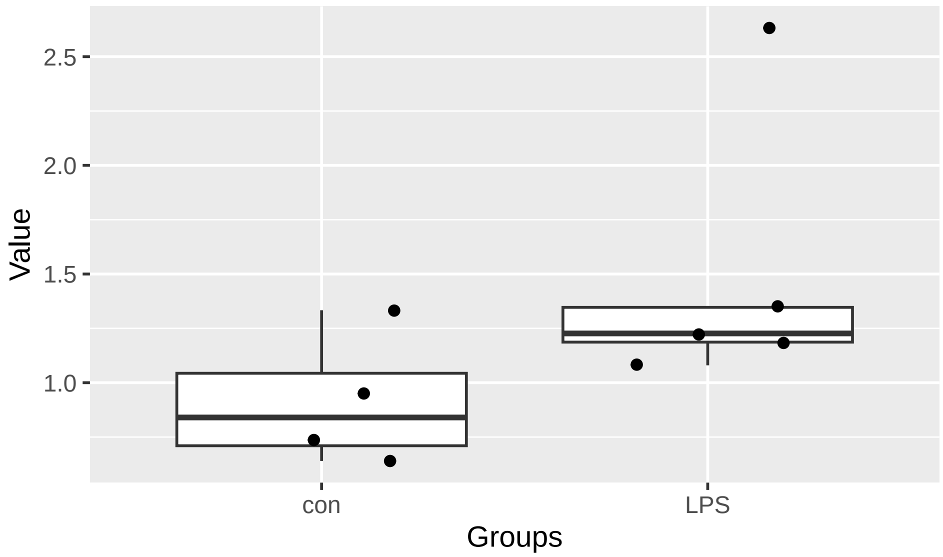


```
##
##  Welch Two Sample t-test
##
## data:  value by group
## t = -1.783, df = 5.9796, p-value = 0.125
## alternative hypothesis: true difference in means between group con and group LPS is not equal to 0
## 95 percent confidence interval:
##  -1.3766238  0.2166231
## sample estimates:
## mean in group con mean in group LPS
##         0.9133332         1.4933336
```

```
## The result of t-test:
##  p = 0.125
##  A statistically significant difference do not exist between groups
```

Fig2_J.LPS-post48hr_qPCR_young_TNFalpha Data analysis

Data structure

```
str(d1)
```

```
## 'data.frame':    11 obs. of  3 variables:
##  $ subject : int  1 2 3 4 5 6 7 8 9 10 ...
##  $ group   : chr  "con" "con" "con" "con" ...
##  $ TNFalpha: num  0.935 1.093 1.449 1.019 0.505 ...
```

Explorative data analysis with graphics

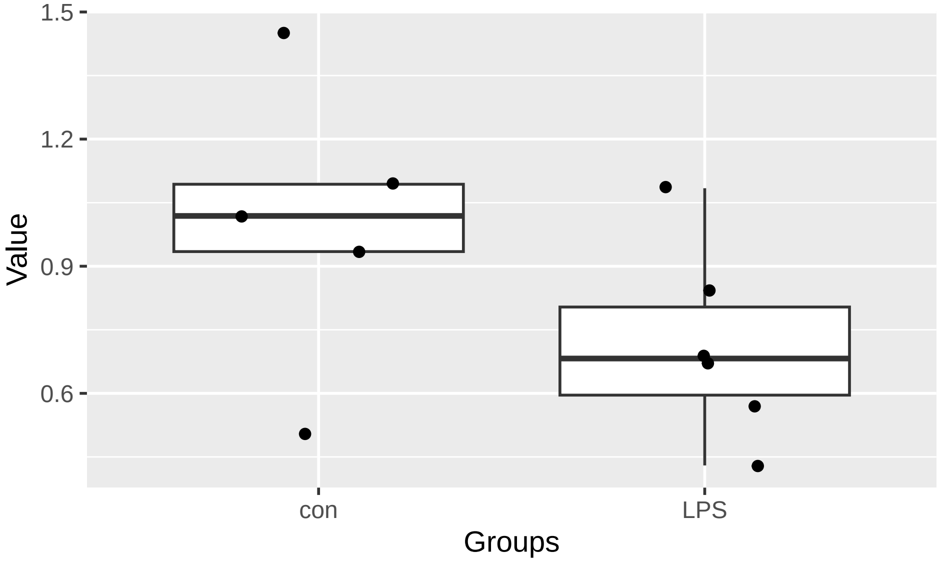


```
##
##  Welch Two Sample t-test
##
## data:  value by group
## t = 1.6046, df = 6.7799, p-value = 0.154
## alternative hypothesis: true difference in means between group con and group LPS is not equal to 0
## 95 percent confidence interval:
##  -0.1377846  0.7078783
## sample estimates:
## mean in group con mean in group LPS
##         1.0000000         0.7149532
```

```
## The result of t-test:
##  p = 0.154
##  A statistically significant difference do not exist between groups
```

Fig2_J.LPS-post48hr_qPCR_young_IL-1beta Data analysis

Data structure

```
str(d1)
```

```
## 'data.frame':    11 obs. of  3 variables:
##  $ subject : int  1 2 3 4 5 6 7 8 9 10 ...
##  $ group   : chr  "con" "con" "con" "con" ...
##  $ IL.1beta: num  0.809 1.044 1.392 0.833 0.922 ...
```

Explorative data analysis with graphics

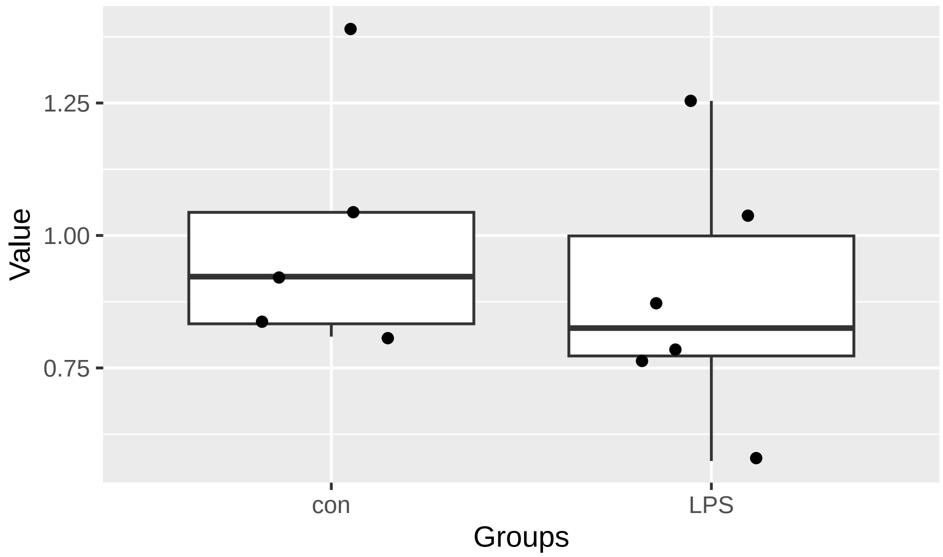


```
##
##  Welch Two Sample t-test
##
## data:  value by group
## t = 0.82167, df = 8.6419, p-value = 0.4333
## alternative hypothesis: true difference in means between group con and group LPS is not equal to 0
## 95 percent confidence interval:
##  -0.2091503  0.4453959
## sample estimates:
## mean in group con mean in group LPS
##         0.9999998         0.8818770
```

```
## The result of t-test:
##  p = 0.433
##  A statistically significant difference do not exist between groups
```

Fig2_J.LPS-post48hr_qPCR_young_IL-10 Data analysis

Data structure

```
str(d1)
```

```
## 'data.frame':    11 obs. of  3 variables:
##  $ subject: int  1 2 3 4 5 6 7 8 9 10 ...
##  $ group  : chr  "con" "con" "con" "con" ...
##  $ IL.10  : num  0.754 0.626 1.44 0.762 1.418 ...
```

Explorative data analysis with graphics

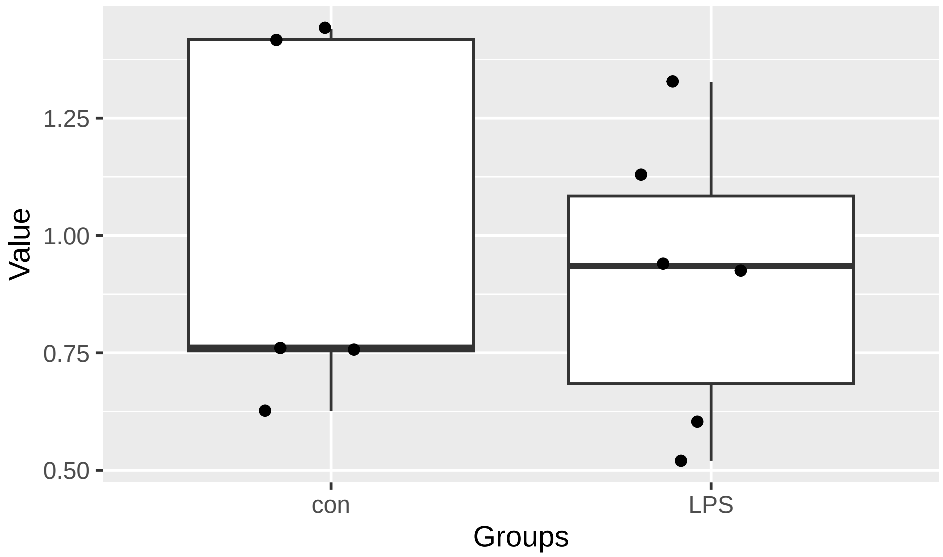


```
##
##  Welch Two Sample t-test
##
## data:  value by group
## t = 0.42109, df = 7.5056, p-value = 0.6855
## alternative hypothesis: true difference in means between group con and group LPS is not equal to 0
## 95 percent confidence interval:
##  -0.4142599  0.5967636
## sample estimates:
## mean in group con mean in group LPS
##         1.0000000         0.9087482
```

```
## The result of t-test:
##  p = 0.685
##  A statistically significant difference do not exist between groups
```

Fig2_J.LPS-post48hr_qPCR_young_IL-6 Data analysis

Data structure

```
str(d1)
```

```
## 'data.frame':    11 obs. of  3 variables:
##  $ subject: int  1 2 3 4 5 6 7 8 9 10 ...
##  $ group  : chr  "con" "con" "con" "con" ...
##  $ IL.6   : num  0.899 0.656 1.646 0.8 0.998 ...
```

Explorative data analysis with graphics

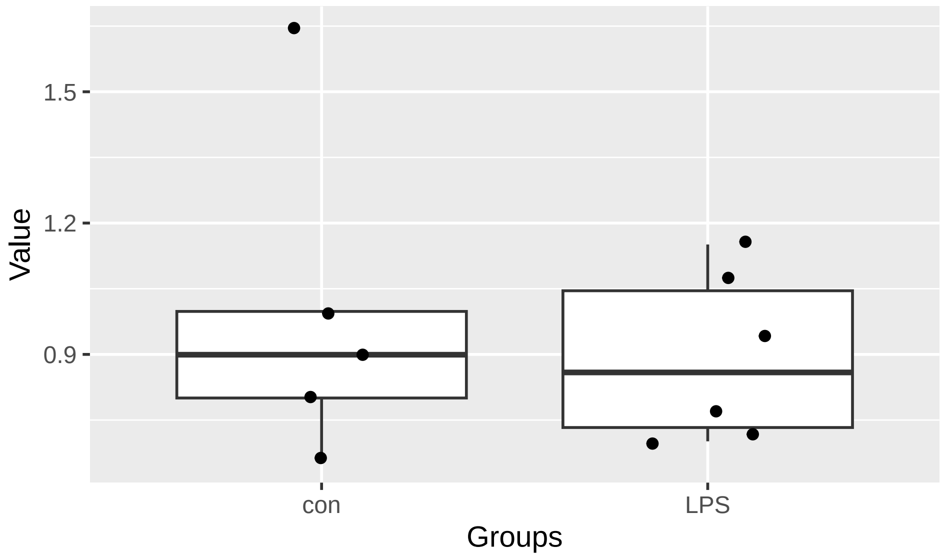


```
##
##  Welch Two Sample t-test
##
## data:  value by group
## t = 0.55912, df = 5.6632, p-value = 0.5975
## alternative hypothesis: true difference in means between group con and group LPS is not equal to 0
## 95 percent confidence interval:
##  -0.3619690  0.5724007
## sample estimates:
## mean in group con mean in group LPS
##         1.0000000         0.8947842
```

```
## The result of t-test:
##  p = 0.597
##  A statistically significant difference do not exist between groups
```

Fig2_K.Western blot_young_old_NLRP3 Data analysis

Boohwi Hong

```
## Present data is ** Western blot_young_old_NLRP3.csv **
##
## **  Data structure  **
## 'data.frame':    9 obs. of  3 variables:
##  $ subject: int  1 2 3 4 5 6 7 8 9
##  $ group  : chr  "young" "young" "young" "young" ...
##  $ NLRP3  : num  0.869 1.247 1.036 0.881 0.966 ...
##
## ** Explorative data analysis with graphics**
```

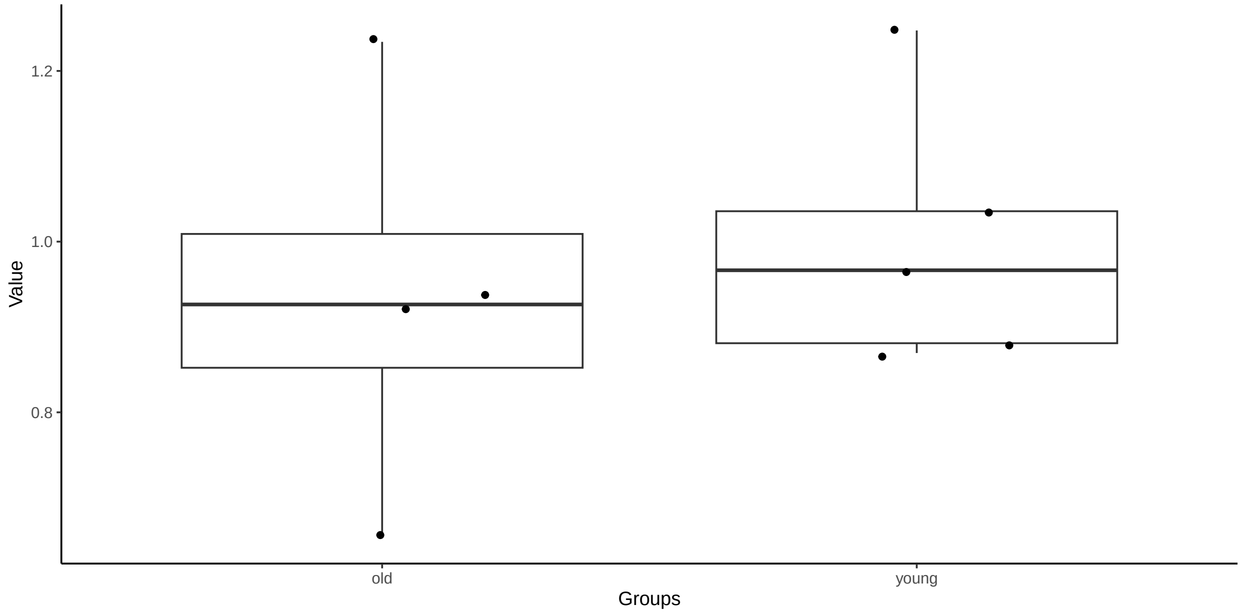


```
## 1. Normality assumption test by Shapiro_Wilk test is
##  p = 0.59
##  Normality assumption was not rejected
## 2. Equal variance test by Bartlett test is
##  p = 0.450
##  Equal variance assumption was not rejected
## 3. The result of anova is
##  p = 0.632
##  A statistically significant difference do not exist between groups
##
```

Fig2_K.Western blot_young_old_pro-IL-1beta_Data analysis

Boohwi Hong

```
## Present data is ** Western blot_young_old_pro-IL-1beta.csv **
##
## **  Data structure  **
## 'data.frame':    9 obs. of  3 variables:
##  $ subject     : int  1 2 3 4 5 6 7 8 9
##  $ group       : chr  "young" "young" "young" "young" ...
##  $ pro.IL.1beta: num  1.037 1.103 1.085 1.182 0.593 ...
##
## ** Explorative data analysis with graphics**
```

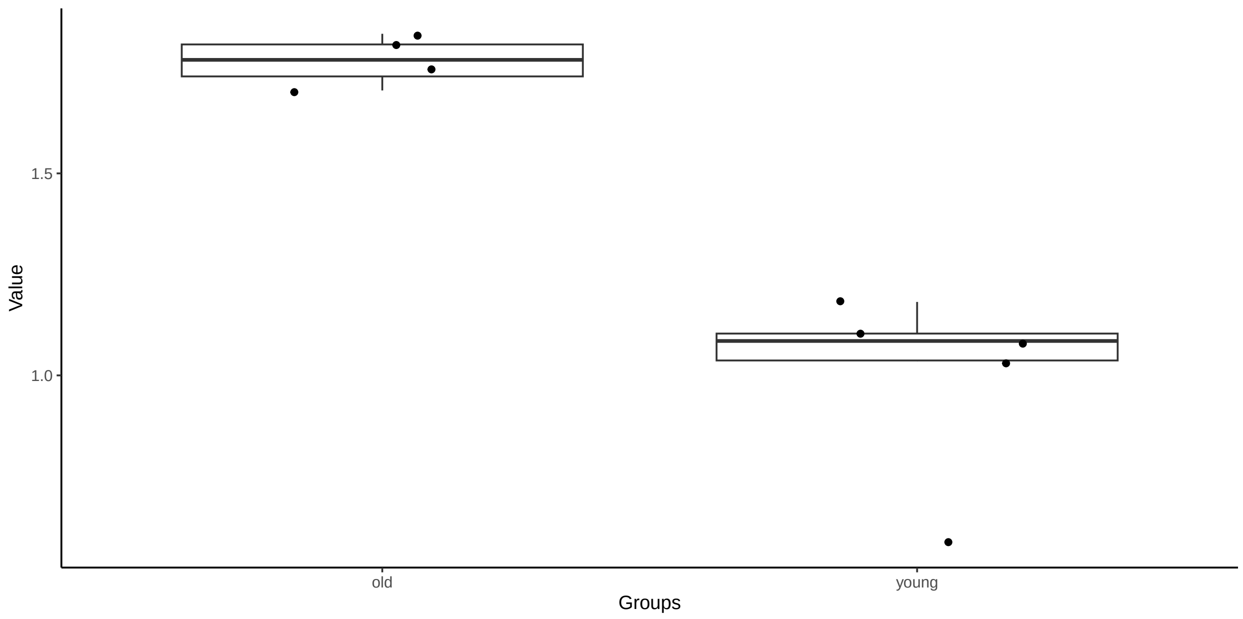


```
## 1. Normality assumption test by Shapiro_Wilk test is
##  p = 0.022
##  Normality assumption was rejected
## 2. The result of Kruskall_Wallis test:
##  p = 0.014
##  A statistically significant difference exist between groups
##
```

Fig2_K.Western blot_young_old_cleaved-caspase1_Data analysis

Boohwi Hong

```
## Present data is ** Western blot_young_old_cleaved-caspase1.csv **
##
## **  Data structure  **
## 'data.frame':    9 obs. of  3 variables:
##  $ subject         : int  1 2 3 4 5 6 7 8 9
##  $ group           : chr  "young" "young" "young" "young" ...
##  $ cleaved.caspase1: num  0.689 0.969 1.664 0.864 0.813 ...
##
## ** Explorative data analysis with graphics**
```

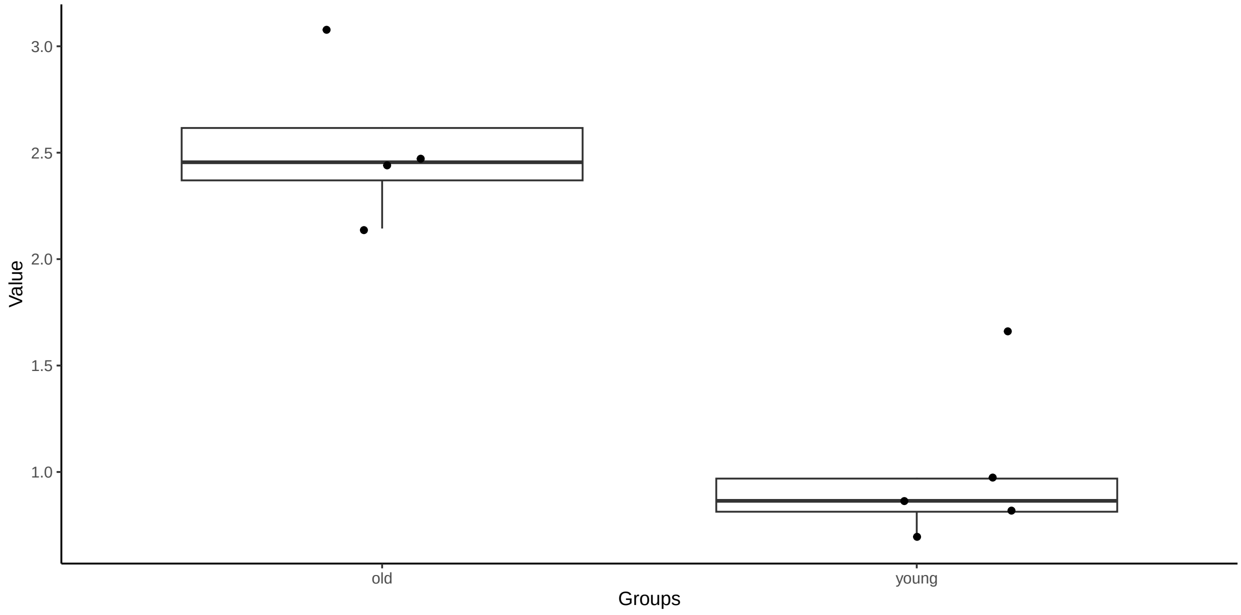


```
## 1. Normality assumption test by Shapiro_Wilk test is
##  p = 0.047
##  Normality assumption was rejected
## 2. The result of Kruskall_Wallis test:
##  p = 0.014
##  A statistically significant difference exist between groups
##
```

Figure3 Statistics

Fig3_A.Barnes maze_Primary Latency

Data analysis using R

Boohwi Hong

Package install

Data import

Data structure

```
str(d1)
```

```
## 'data.frame':    112 obs. of  4 variables:
##  $ subject: int  1 2 3 4 5 6 7 8 9 10 ...
##  $ group  : chr  "con" "con" "con" "con" ...
##  $ trial  : chr  "day1" "day1" "day1" "day1" ...
##  $ value  : num  56.6 60.6 40.3 76.9 60.8 ...
```

```
shapiro.test(d1$value)
```

```
##
##  Shapiro-Wilk normality test
##
## data:  d1$value
## W = 0.92802, p-value = 1.394e-05
```

Explorative data analysis with graphics

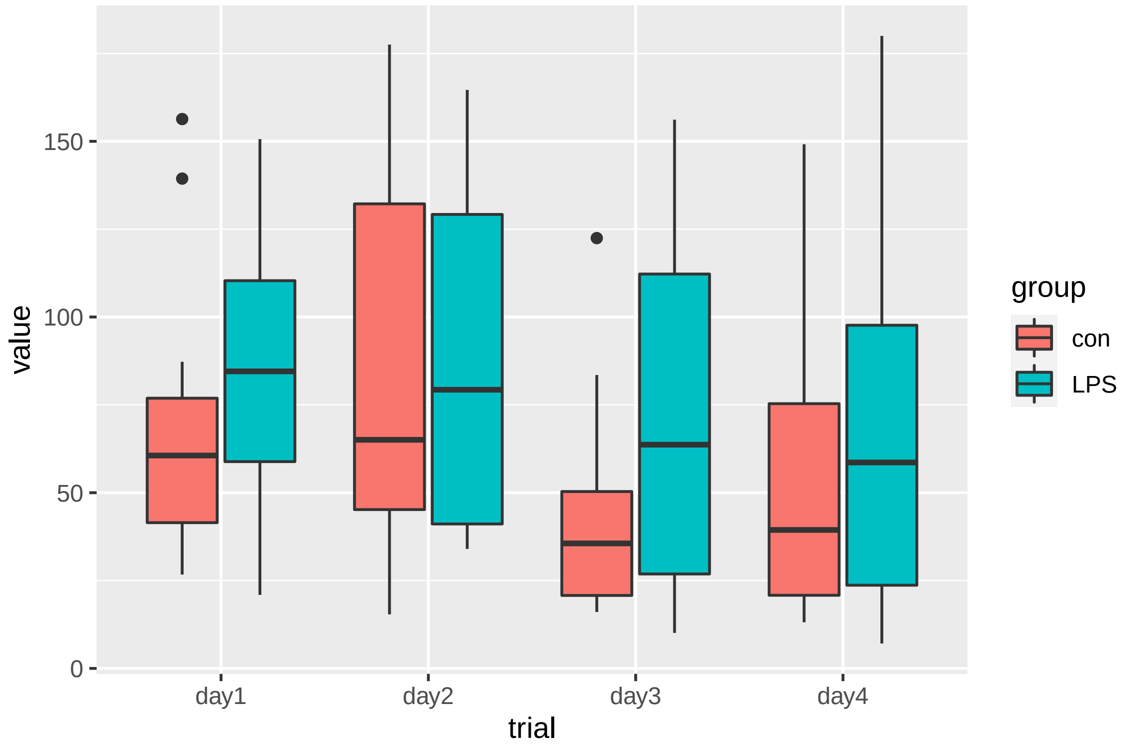

Model fit

```
##  F1 LD F1 Model
##  -----------------------
##  Check that the order of the time and group levels are correct.
##  Time level:   day1 day2 day3 day4
##  Group level:   con LPS
##  If the order is not correct, specify the correct order in time.order or group.order.

##              Statistic df     p-value
## group         1.575915  1 0.209350065
## trial        11.625510  3 0.008782558
## group:trial   1.411162  3 0.702920459

##              Statistic       df     p-value
## group        1.5759152 1.000000 0.209350065
## trial        4.5316366 2.933837 0.003779748
## group:trial 0.4698419 2.933837 0.699021819

##        Statistic df1      df2   p-value
## group  1.575915   1 25.96745 0.2205297
```

Interpretation of result

```
## 1. Difference do not exist between groups
##  p = 0.209

## 2. Difference exist between measurement points
##  p = 0.004

## 3. Significant interaction do not exist between groups and measurement points
##  p = 0.699
```

Fig3_A.Barnes maze_Primary length

Data analysis using R

Boohwi Hong

Package install

Data import

Data structure

```
str(d1)
```

```
## 'data.frame':    112 obs. of  4 variables:
##  $ subject: int  1 2 3 4 5 6 7 8 9 10 ...
##  $ group  : chr  "con" "con" "con" "con" ...
##  $ trial  : chr  "day1" "day1" "day1" "day1" ...
##  $ value  : num  397 477 356 631 406 ...
```

```
shapiro.test(d1$value)
```

```
##
##  Shapiro-Wilk normality test
##
## data:  d1$value
## W = 0.96007, p-value = 0.002021
```

Explorative data analysis with graphics

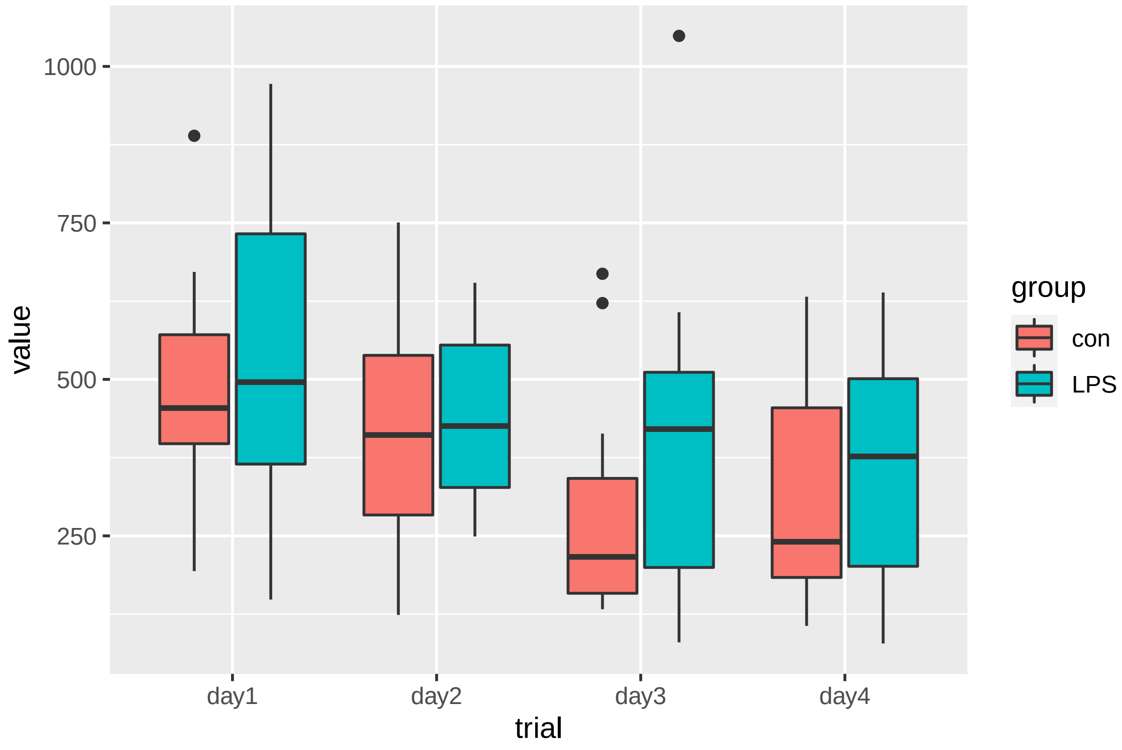

Model fit

```
##  F1 LD F1 Model
##  -----------------------
##  Check that the order of the time and group levels are correct.
##  Time level:   day1 day2 day3 day4
##  Group level:   con LPS
##  If the order is not correct, specify the correct order in time.order or group.order.

##               Statistic df    p-value
## group         1.8014282  1 0.17953993
## trial        12.8554641  3 0.00495983
## group:trial   0.5542484  3 0.90682148

##              Statistic       df     p-value
## group       1.8014282 1.000000 0.179539925
## trial       5.2206949 2.799798 0.001757758
## group:trial 0.2451294 2.799798 0.851920723

##        Statistic df1      df2  p-value
## group   1.801428   1 25.78564 0.191239
```

Interpretation of result

```
## 1. Difference do not exist between groups
##  p = 0.180

## 2. Difference exist between measurement points
##  p = 0.002

## 3. Significant interaction do not exist between groups and measurement points
##  p = 0.852
```

Fig3_A.Barnes maze_Primary errors

Data analysis using R

Boohwi Hong

Package install

Data import

Data structure

```
str(d1)
```

```
## 'data.frame':    112 obs. of  4 variables:
##  $ subject: int  1 2 3 4 5 6 7 8 9 10 ...
##  $ group  : chr  "con" "con" "con" "con" ...
##  $ trial  : int  1 1 1 1 1 1 1 1 1 1 ...
##  $ value  : num  7.33 11.33 7 6.67 6.67 ...
```

```
shapiro.test(d1$value)
```

```
##
##  Shapiro-Wilk normality test
##
## data:  d1$value
## W = 0.95906, p-value = 0.001692
```

Explorative data analysis with graphics

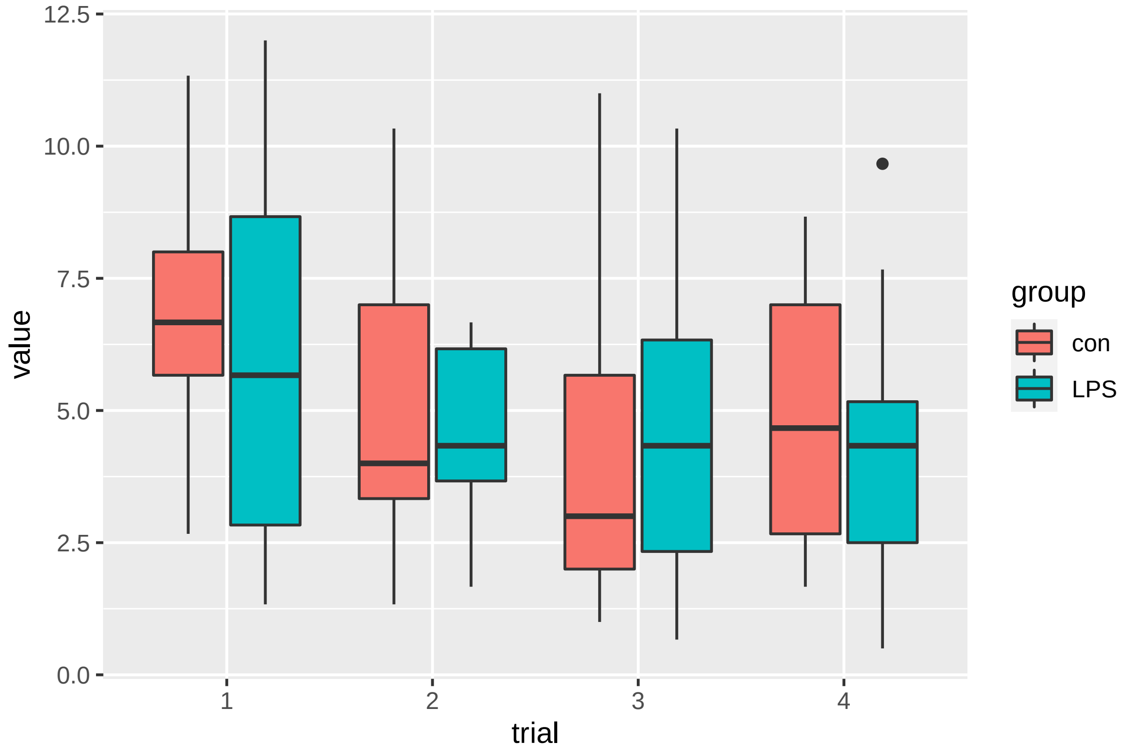

Model fit

```
##  F1 LD F1 Model
##  -----------------------
##  Check that the order of the time and group levels are correct.
##  Time level:   1 2 3 4
##  Group level:   con LPS
##  If the order is not correct, specify the correct order in time.order or group.order.
```

```
##             Statistic df    p-value
## group        0.631484  1 0.42681151
## trial        8.033390  3 0.04532666
## group:trial  1.477013  3 0.68758576
```

```
##             Statistic       df    p-value
## group       0.6314840 1.000000 0.42681151
## trial       2.9622190 2.710279 0.03580191
## group:trial 0.5488226 2.710279 0.63095804
```

```
##       Statistic df1      df2   p-value
## group  0.631484   1 23.99755 0.4346006
```

Interpretation of result

```
## 1. Difference do not exist between groups
##  p = 0.427

## 2. Difference exist between measurement points
##  p = 0.036

## 3. Significant interaction do not exist between groups and measurement points
##  p = 0.631
```

Fig3_C.Barnes maze_Probe test_Duration Data analysis

Data structure

```
str(d1)
```

```
## 'data.frame':    28 obs. of  3 variables:
##  $ subject : int  1 2 3 4 5 6 7 8 9 10 ...
##  $ group   : chr  "con" "con" "con" "con" ...
##  $ Duration: num  19.8 25 29.5 43.2 28.4 ...
```

Explorative data analysis with graphics

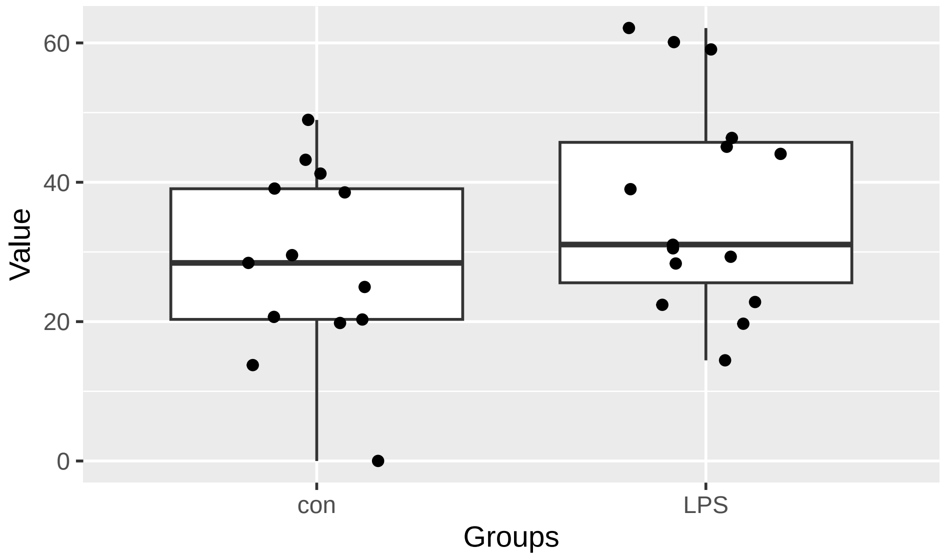


```
##
##  Welch Two Sample t-test
##
## data:  value by group
## t = -1.5687, df = 25.964, p-value = 0.1288
## alternative hypothesis: true difference in means between group con and group LPS is not equal to 0
## 95 percent confidence interval:
##  -19.896881   2.673506
## sample estimates:
## mean in group con mean in group LPS
##          28.35339          36.96508
```

```
## The result of t-test:
##  p = 0.129
##  A statistically significant difference do not exist between groups
```

Fig3_C.Barnes maze_Probe test_Distance Data analysis

Data structure

```
str(d1)
```

```
## 'data.frame':    28 obs. of  3 variables:
##  $ subject : int  1 2 3 4 5 6 7 8 9 10 ...
##  $ group   : chr  "con" "con" "con" "con" ...
##  $ Distance: num  0.0783 0.2561 0.0864 1.679 1.6421 ...
```

Explorative data analysis with graphics

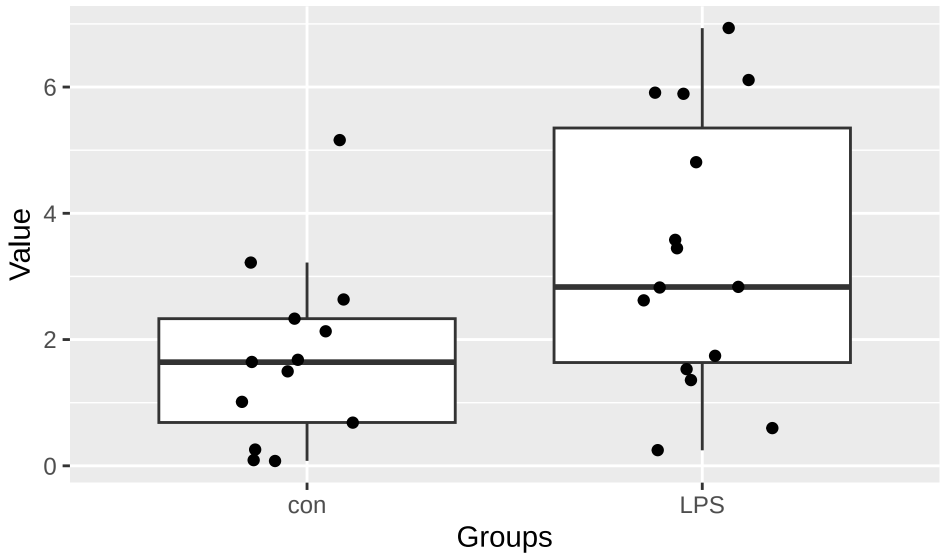


```
##
##  Welch Two Sample t-test
##
## data:  value by group
## t = -2.4106, df = 24.581, p-value = 0.02375
## alternative hypothesis: true difference in means between group con and group LPS is not equal to 0
## 95 percent confidence interval:
##  -3.0384817 -0.2373261
## sample estimates:
## mean in group con mean in group LPS
##          1.724300          3.362204
```

```
## The result of t-test:
##  p = 0.024
##  A statistically significant difference exist between groups
```

Fig3_C.Barnes maze_Probe test_Latency Data analysis

Data structure

```
str(d1)
```

```
## 'data.frame':    28 obs. of  3 variables:
##  $ subject: int  1 2 3 4 5 6 7 8 9 10 ...
##  $ group  : chr  "con" "con" "con" "con" ...
##  $ Latency: num  0.534 2.903 0.5 14.581 15.582 ...
```

Explorative data analysis with graphics

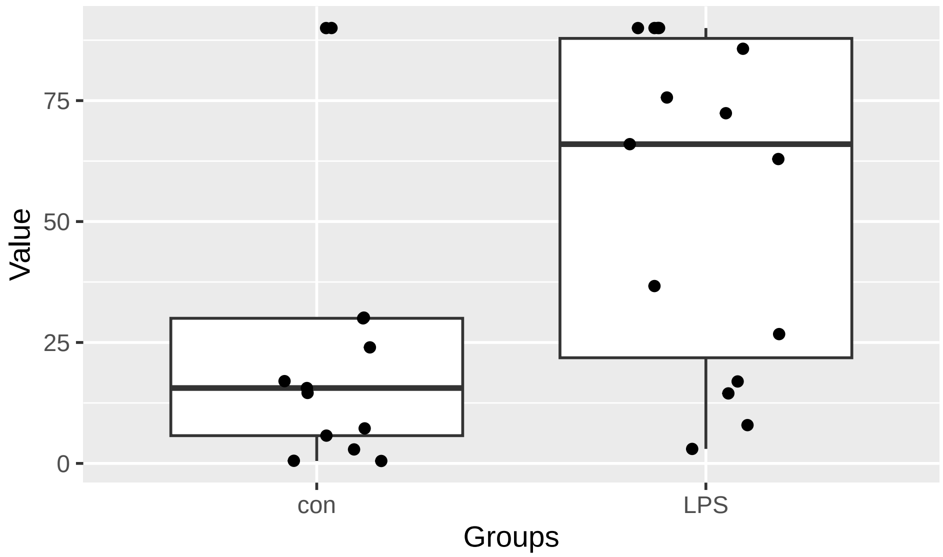


```
##
##  Welch Two Sample t-test
##
## data:  value by group
## t = -2.4719, df = 25.941, p-value = 0.02032
## alternative hypothesis: true difference in means between group con and group LPS is not equal to 0
## 95 percent confidence interval:
##  -54.915542  -5.047479
## sample estimates:
## mean in group con mean in group LPS
##          25.24684          55.22835
```

```
## The result of t-test:
##  p = 0.02
##  A statistically significant difference exist between groups
```

Fig3_D.Fear chamber test Data analysis

Data structure

```
str(d1)
```

```
## 'data.frame':    28 obs. of  3 variables:
##  $ subject : int  1 2 3 4 5 6 7 8 9 10 ...
##  $ group   : chr  "con" "con" "con" "con" ...
##  $ Freazing: num  51.2 40.3 30.9 15.7 45.3 ...
```

Explorative data analysis with graphics

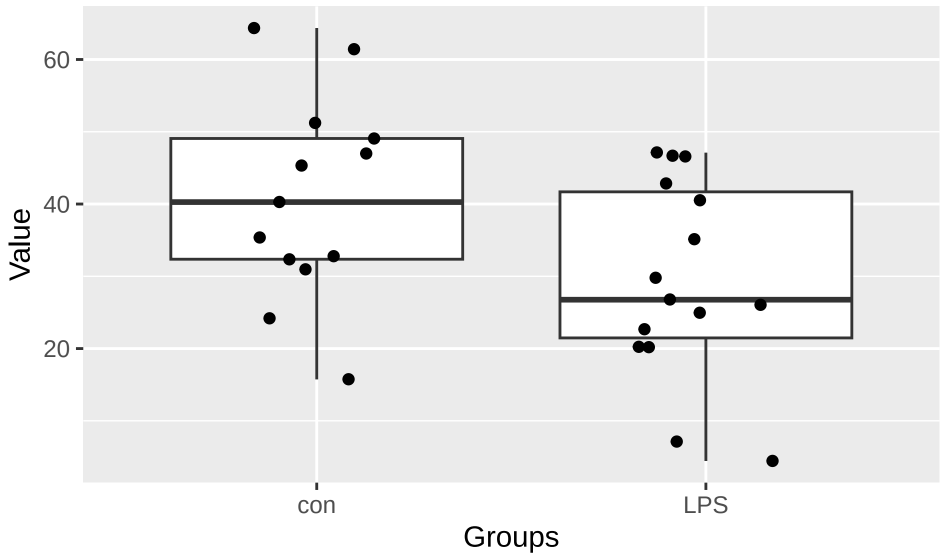


```
##
##  Welch Two Sample t-test
##
## data:  value by group
## t = 2.1588, df = 25.191, p-value = 0.04058
## alternative hypothesis: true difference in means between group con and group LPS is not equal to 0
## 95 percent confidence interval:
##   0.5269642 22.2098563
## sample estimates:
## mean in group con mean in group LPS
##          40.77308          29.40467
```

```
## The result of t-test:
##  p = 0.041
##  A statistically significant difference exist between groups
```

Figure4 Statistics

Fig4_B.young mice_rotenone_OCR_Basal Data analysis

Data structure

```
str(d1)
```

```
## 'data.frame':    19 obs. of  3 variables:
##  $ subject: int  1 2 3 4 5 6 7 8 9 10 ...
##  $ group  : chr  "con" "con" "con" "con" ...
##  $ OCR    : num  294 211 290 300 325 ...
```

Explorative data analysis with graphics

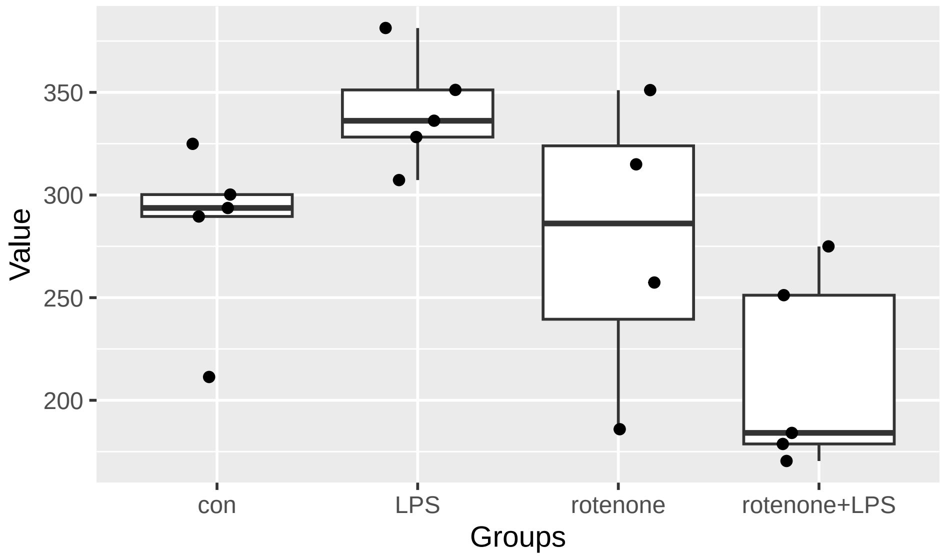


```
##              Df Sum Sq Mean Sq F value Pr(>F)
## d1[, 2]       3  41770   13923   5.949  0.007 **
## Residuals    15  35104    2340
## ---
## Signif. codes:  0 '***' 0.001 '**' 0.01 '*' 0.05 '.' 0.1 ' ' 1
```

```
## The result of anova is
##  p = 0.007
##  A statistically significant difference exist between groups
```

```
##   Tukey multiple comparisons of means
##     95% family-wise confidence level
##
## Fit: aov(formula = d1[, 3] ~ d1[, 2], data = d1)
##
## $`d1[, 2]`
##                              diff        lwr       upr     p adj
## LPS-con                 56.892983  -31.28921 145.07518 0.2857524
## rotenone-con            -6.638199 -100.16954  86.89314 0.9968350
## rotenone+LPS-con       -72.057223 -160.23942  16.12497 0.1294538
## rotenone-LPS           -63.531182 -157.06253  30.00016 0.2468473
## rotenone+LPS-LPS      -128.950206 -217.13240 -40.76801 0.0037309
## rotenone+LPS-rotenone  -65.419024 -158.95037  28.11232 0.2257379
```

Fig4_B.young mice_rotenone_OCR_State3 Data analysis

Data structure

```
str(d1)
```

```
## 'data.frame':    19 obs. of  3 variables:
##  $ subject: int  1 2 3 4 5 6 7 8 9 10 ...
##  $ group  : chr  "con" "con" "con" "con" ...
##  $ OCR    : num  1238 902 1228 1211 1048 ...
```

Explorative data analysis with graphics

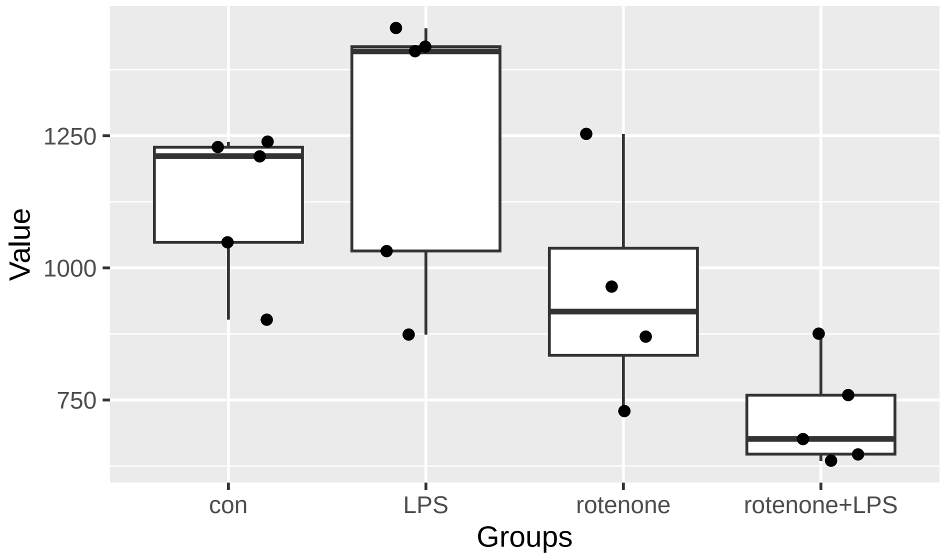


```
##             Df Sum Sq Mean Sq F value  Pr(>F)
## d1[, 2]      3 762191  254064   6.834 0.00401 **
## Residuals   15 557642   37176
## ---
## Signif. codes:  0 '***' 0.001 '**' 0.01 '*' 0.05 '.' 0.1 ' ' 1
```

```
## The result of anova is
##  p = 0.004
##  A statistically significant difference exist between groups
```

```
##   Tukey multiple comparisons of means
##     95% family-wise confidence level
##
## Fit: aov(formula = d1[, 3] ~ d1[, 2], data = d1)
##
## $`d1[, 2]`
##                             diff       lwr        upr     p adj
## LPS-con                 111.7427 -239.7194  463.20489 0.7965979
## rotenone-con           -171.3073 -544.0892  201.47461 0.5623868
## rotenone+LPS-con       -407.0108 -758.4730  -55.54867 0.0208876
## rotenone-LPS           -283.0500 -655.8320   89.73189 0.1713149
## rotenone+LPS-LPS       -518.7536 -870.2157 -167.29140 0.0034534
## rotenone+LPS-rotenone -235.7035 -608.4854  137.07839 0.3015474
```

Fig4_B.young mice_rotenone_OCR_State4o Data analysis

Data structure

```
str(d1)
```

```
## 'data.frame':    19 obs. of  3 variables:
##  $ subject: int  1 2 3 4 5 6 7 8 9 10 ...
##  $ group  : chr  "con" "con" "con" "con" ...
##  $ OCR    : num  233 145 208 202 208 ...
```

Explorative data analysis with graphics

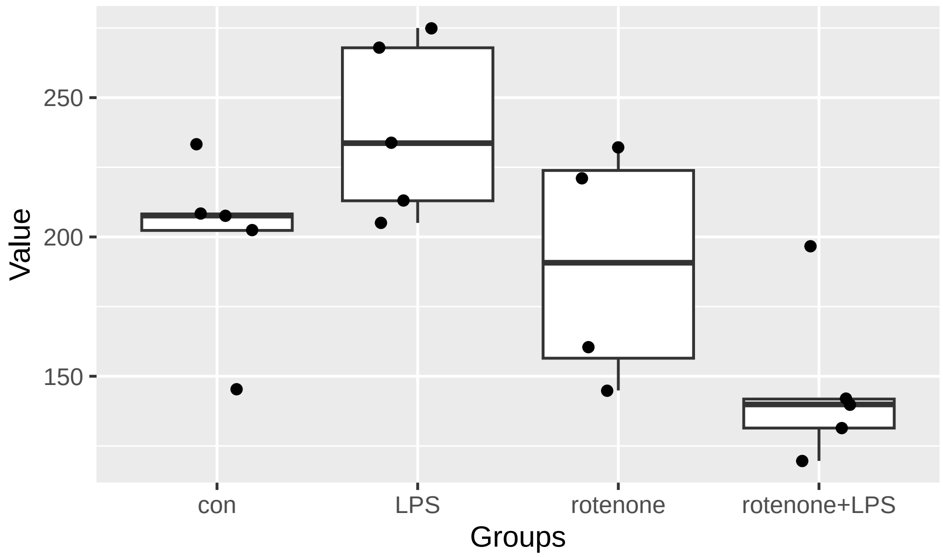


```
##             Df Sum Sq Mean Sq F value  Pr(>F)
## d1[, 2]      3  21878    7293    6.29 0.00562 **
## Residuals   15  17390    1159
## ---
## Signif. codes:  0 '***' 0.001 '**' 0.01 '*' 0.05 '.' 0.1 ' ' 1
```

```
## The result of anova is
##  p = 0.006
##  A statistically significant difference exist between groups
```

```
##   Tukey multiple comparisons of means
##     95% family-wise confidence level
##
## Fit: aov(formula = d1[, 3] ~ d1[, 2], data = d1)
##
## $`d1[, 2]`
##                              diff        lwr        upr     p adj
## LPS-con                 39.557092  -22.50877 101.622952 0.2952808
## rotenone-con            -9.758186  -75.58897  56.072601 0.9729040
## rotenone+LPS-con       -53.467196 -115.53306   8.598665 0.1036416
## rotenone-LPS           -49.315278 -115.14606  16.515509 0.1797274
## rotenone+LPS-LPS       -93.024288 -155.09015 -30.958427 0.0030361
## rotenone+LPS-rotenone -43.709010 -109.53980  22.121777 0.2637949
```

Fig4_B.young mice_rotenone_OCR_State3u Data analysis

Data structure

```
str(d1)
```

```
## 'data.frame':    19 obs. of  3 variables:
##  $ subject: int  1 2 3 4 5 6 7 8 9 10 ...
##  $ group  : chr  "con" "con" "con" "con" ...
##  $ OCR    : int  1210 737 1010 1054 952 1121 1307 1228 1212 952 ...
```

Explorative data analysis with graphics

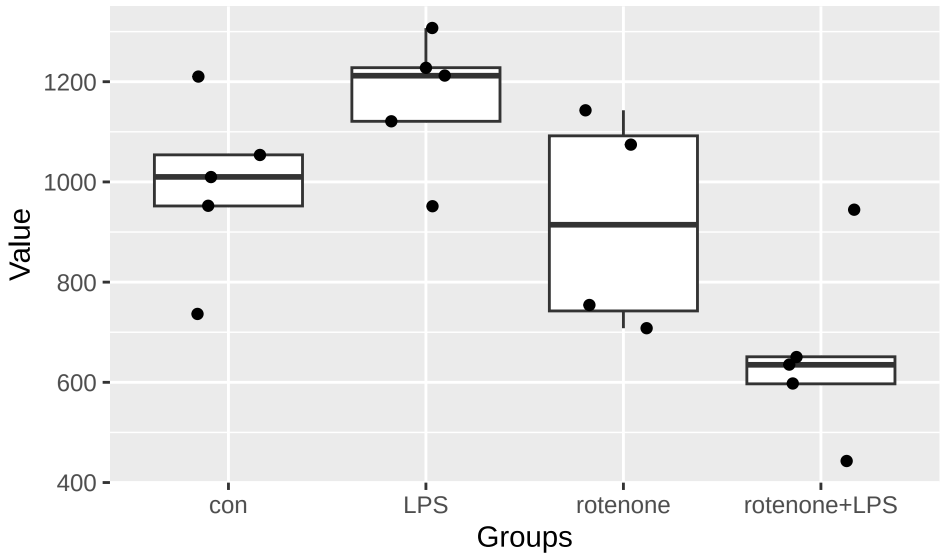


```
##             Df Sum Sq Mean Sq F value  Pr(>F)
## d1[, 2]      3 673886  224629   7.153 0.00331 **
## Residuals   15 471032   31402
## ---
## Signif. codes:  0 '***' 0.001 '**' 0.01 '*' 0.05 '.' 0.1 ' ' 1
```

```
## The result of anova is
##  p = 0.003
##  A statistically significant difference exist between groups
```

```
##   Tukey multiple comparisons of means
##     95% family-wise confidence level
##
## Fit: aov(formula = d1[, 3] ~ d1[, 2], data = d1)
##
## $`d1[, 2]`
##                          diff       lwr        upr     p adj
## LPS-con                 171.4 -151.6174  494.41742 0.4455817
## rotenone-con            -72.6 -415.2117  270.01172 0.9270814
## rotenone+LPS-con       -338.4 -661.4174  -15.38258 0.0385709
## rotenone-LPS           -244.0 -586.6117   98.61172 0.2131421
## rotenone+LPS-LPS       -509.8 -832.8174 -186.78258 0.0019426
## rotenone+LPS-rotenone -265.8 -608.4117   76.81172 0.1583467
```

Fig4_C.young mice_rotenone_RCR Data analysis

Data structure

```
str(d1)
```

```
## 'data.frame':    19 obs. of  3 variables:
##  $ subject: int  1 2 3 4 5 6 7 8 9 10 ...
##  $ group  : chr  "con" "con" "con" "con" ...
##  $ RCR    : num  4.22 3.51 3.69 3.95 3.06 ...
```

Explorative data analysis with graphics

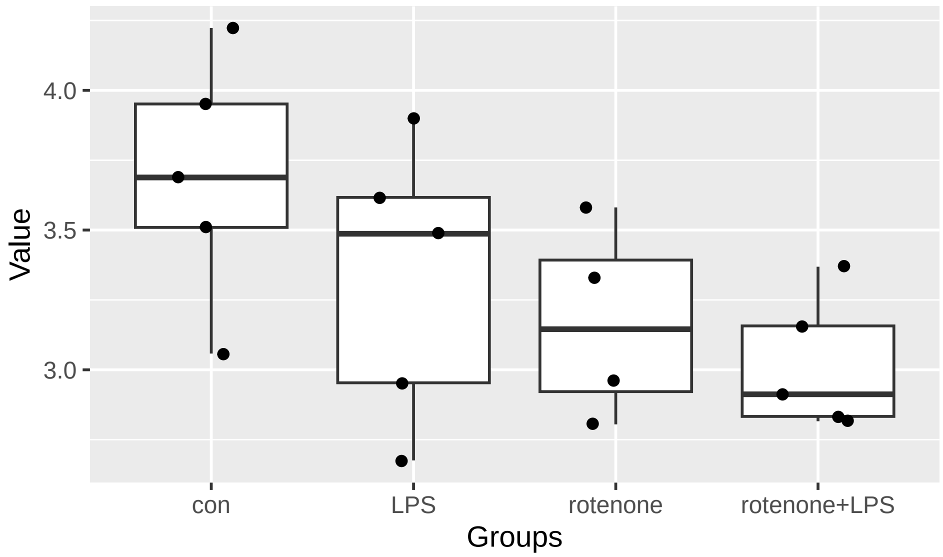


```
##              Df Sum Sq Mean Sq F value Pr(>F)
## d1[, 2]       3  1.216  0.4052   2.547  0.095 .
## Residuals    15  2.386  0.1591
## ---
## Signif. codes:  0 '***' 0.001 '**' 0.01 '*' 0.05 '.' 0.1 ' ' 1
```

```
## The result of anova is
##  p = 0.0950
##  A statistically significant difference do not exist between groups
```

Fig4_C.young mice_rotenone_RCRu Data analysis

Data structure

```
str(d1)
```

```
## 'data.frame':    19 obs. of  3 variables:
##  $ subject: int  1 2 3 4 5 6 7 8 9 10 ...
##  $ group  : chr  "con" "con" "con" "con" ...
##  $ RCRu   : num  3.88 2.45 2.66 3.08 2.33 ...
```

Explorative data analysis with graphics

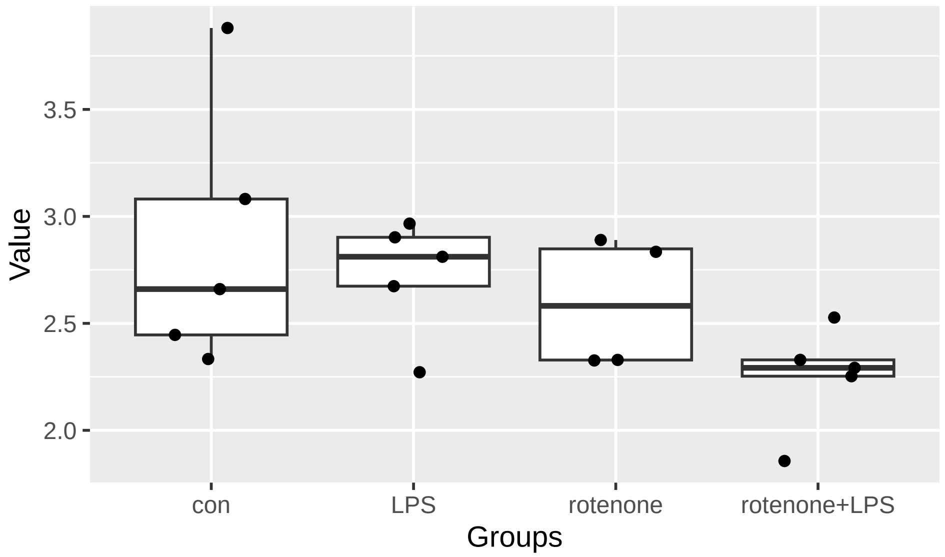


```
##             Df Sum Sq Mean Sq F value Pr(>F)
## d1[, 2]      3  1.074  0.3581   2.229  0.127
## Residuals   15  2.409  0.1606
```

```
## The result of anova is
##  p = 0.1269
##  A statistically significant difference do not exist between groups
```

Fig4_D.young mice_rotenone_qPCR_TNFalpha Data analysis

Data structure

```
str(d1)
```

```
## 'data.frame':    19 obs. of  3 variables:
##  $ subject : int  1 2 3 4 5 6 7 8 9 10 ...
##  $ group   : chr  "con" "con" "con" "con" ...
##  $ TNFalpha: num  0.89 1.22 0.8 1.01 1.07 ...
```

Explorative data analysis with graphics

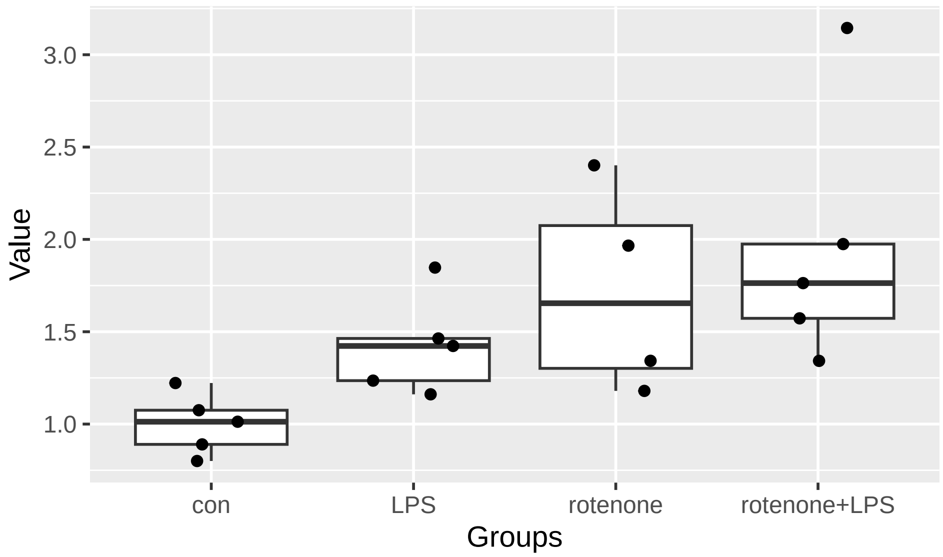


```
##             Df Sum Sq Mean Sq F value Pr(>F)
## d1[, 2]      3  2.526  0.8419   3.797  0.033 *
## Residuals   15  3.326  0.2217
## ---
## Signif. codes:  0 '***' 0.001 '**' 0.01 '*' 0.05 '.' 0.1 ' ' 1
```

```
## The result of anova is
##  p = 0.033
##  A statistically significant difference exist between groups
```

```
##   Tukey multiple comparisons of means
##     95% family-wise confidence level
##
## Fit: aov(formula = d1[, 3] ~ d1[, 2], data = d1)
##
## $`d1[, 2]`
##                             diff        lwr      upr     p adj
## LPS-con               0.4260386 -0.4322830 1.284360 0.5007225
## rotenone-con          0.7223600 -0.1880275 1.632748 0.1453972
## rotenone+LPS-con      0.9595036  0.1011820 1.817825 0.0261478
## rotenone-LPS          0.2963214 -0.6140661 1.206709 0.7852047
## rotenone+LPS-LPS      0.5334650 -0.3248566 1.391787 0.3151799
## rotenone+LPS-rotenone 0.2371436 -0.6732439 1.147531 0.8749356
```

Fig4_D.young mice_rotenone_qPCR_IL-1beta Data analysis

Data structure

```
str(d1)
```

```
## 'data.frame':    19 obs. of  3 variables:
##  $ subject : int  1 2 3 4 5 6 7 8 9 10 ...
##  $ group   : chr  "con" "con" "con" "con" ...
##  $ IL.1beta: num  0.778 1.042 1.104 1.33 0.747 ...
```

Explorative data analysis with graphics

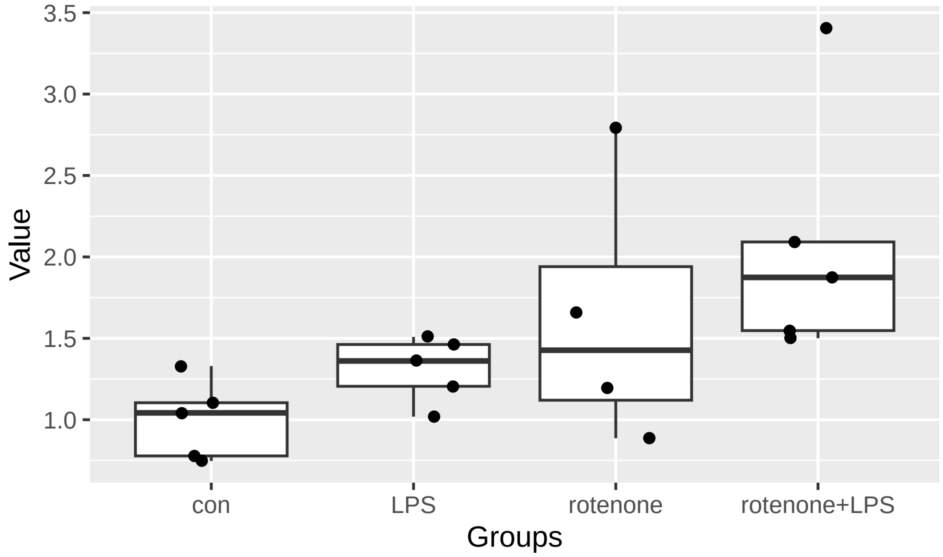


```
##             Df Sum Sq Mean Sq F value Pr(>F)
## d1[, 2]      3  3.204   1.068   3.266 0.0509 .
## Residuals   15  4.905   0.327
## ---
## Signif. codes:  0 '***' 0.001 '**' 0.01 '*' 0.05 '.' 0.1 ' ' 1
```

```
## The result of anova is
##  p = 0.0509
##  A statistically significant difference do not exist between groups
```

Fig4_D.young mice_rotenone_qPCR_IL-10 Data analysis

Data structure

```
str(d1)
```

```
## 'data.frame':    19 obs. of  3 variables:
##  $ subject: int  1 2 3 4 5 6 7 8 9 10 ...
##  $ group  : chr  "con" "con" "con" "con" ...
##  $ IL.10  : num  0.716 1.103 1.103 1.254 0.824 ...
```

Explorative data analysis with graphics

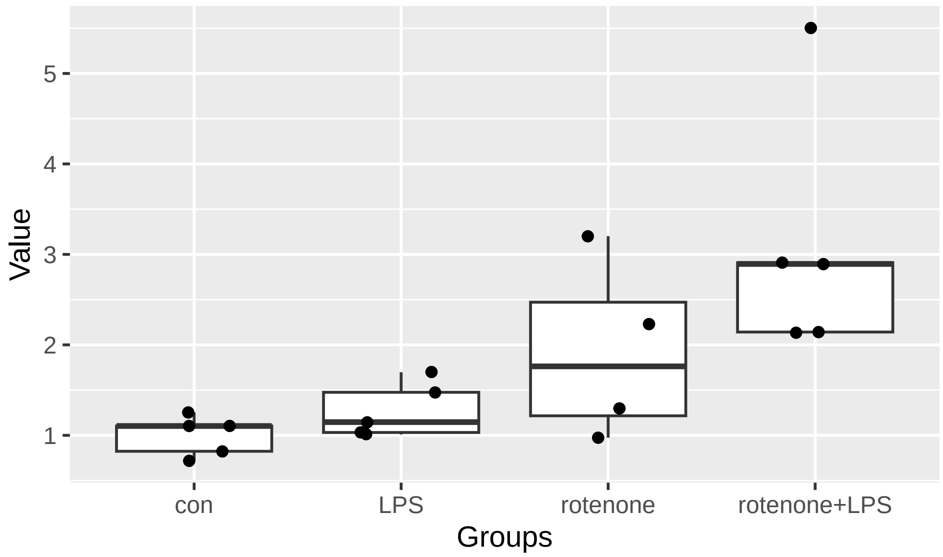


```
##             Df Sum Sq Mean Sq F value  Pr(>F)
## d1[, 2]      3  13.30   4.435   5.899 0.00724 **
## Residuals   15  11.28   0.752
## ---
## Signif. codes:  0 '***' 0.001 '**' 0.01 '*' 0.05 '.' 0.1 ' ' 1
```

```
## The result of anova is
##  p = 0.007
##  A statistically significant difference exist between groups
```

```
##   Tukey multiple comparisons of means
##     95% family-wise confidence level
##
## Fit: aov(formula = d1[, 3] ~ d1[, 2], data = d1)
##
## $`d1[, 2]`
##                             diff        lwr      upr     p adj
## LPS-con                0.2722066 -1.3083880 1.852801 0.9586713
## rotenone-con           0.9251435 -0.7513302 2.601617 0.4128830
## rotenone+LPS-con       2.1160458  0.5354512 3.696640 0.0075171
## rotenone-LPS           0.6529369 -1.0235368 2.329411 0.6818143
## rotenone+LPS-LPS       1.8438392  0.2632446 3.424434 0.0199147
## rotenone+LPS-rotenone 1.1909023 -0.4855714 2.867376 0.2149021
```

Fig4_D.young mice_rotenone_qPCR_IL-6 Data analysis

Data structure

```
str(d1)
```

```
## 'data.frame':    19 obs. of  3 variables:
##  $ subject: int  1 2 3 4 5 6 7 8 9 10 ...
##  $ group  : chr  "con" "con" "con" "con" ...
##  $ IL.6   : num  0.812 1.006 1.12 1.08 0.982 ...
```

Explorative data analysis with graphics

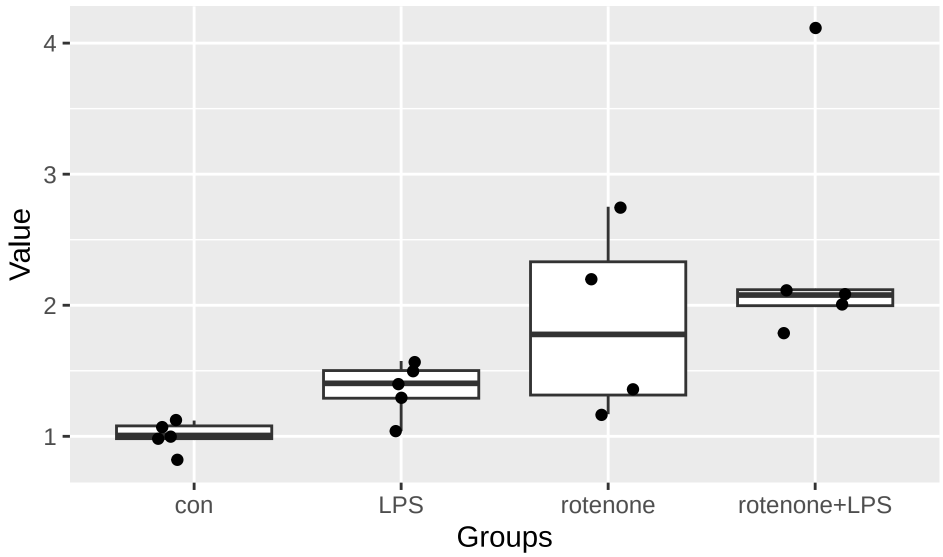


```
##              Df Sum Sq Mean Sq F value Pr(>F)
## d1[, 2]       3  5.674  1.8914   5.136 0.0122 *
## Residuals    15  5.525  0.3683
## ---
## Signif. codes:  0 '***' 0.001 '**' 0.01 '*' 0.05 '.' 0.1 ' ' 1
```

```
## The result of anova is
##  p = 0.012
##  A statistically significant difference exist between groups
```

```
##   Tukey multiple comparisons of means
##     95% family-wise confidence level
##
## Fit: aov(formula = d1[, 3] ~ d1[, 2], data = d1)
##
## $`d1[, 2]`
##                              diff        lwr      upr     p adj
## LPS-con               0.3620128 -0.7442230 1.468249 0.7825220
## rotenone-con          0.8689120 -0.3044283 2.042252 0.1870809
## rotenone+LPS-con      1.4188310  0.3125952 2.525067 0.0103405
## rotenone-LPS          0.5068992 -0.6664411 1.680239 0.6093547
## rotenone+LPS-LPS      1.0568182 -0.0494176 2.163054 0.0635486
## rotenone+LPS-rotenone 0.5499190 -0.6234213 1.723259 0.5469120
```

Fig4_E.Western blot_rotenone_NLRP3_Data analysis

Boohwi Hong

```
## Present data is ** Western blot_rotenone_NLRP3.csv **
##
## **  Data structure  **
## 'data.frame':    10 obs. of  3 variables:
##  $ subject: int  1 2 3 4 5 6 7 8 9 10
##  $ group  : chr  "con" "con" "con" "con" ...
##  $ NLRP3  : num  0.844 0.886 0.938 1.253 1.079 ...
##
## ** Explorative data analysis with graphics**
```

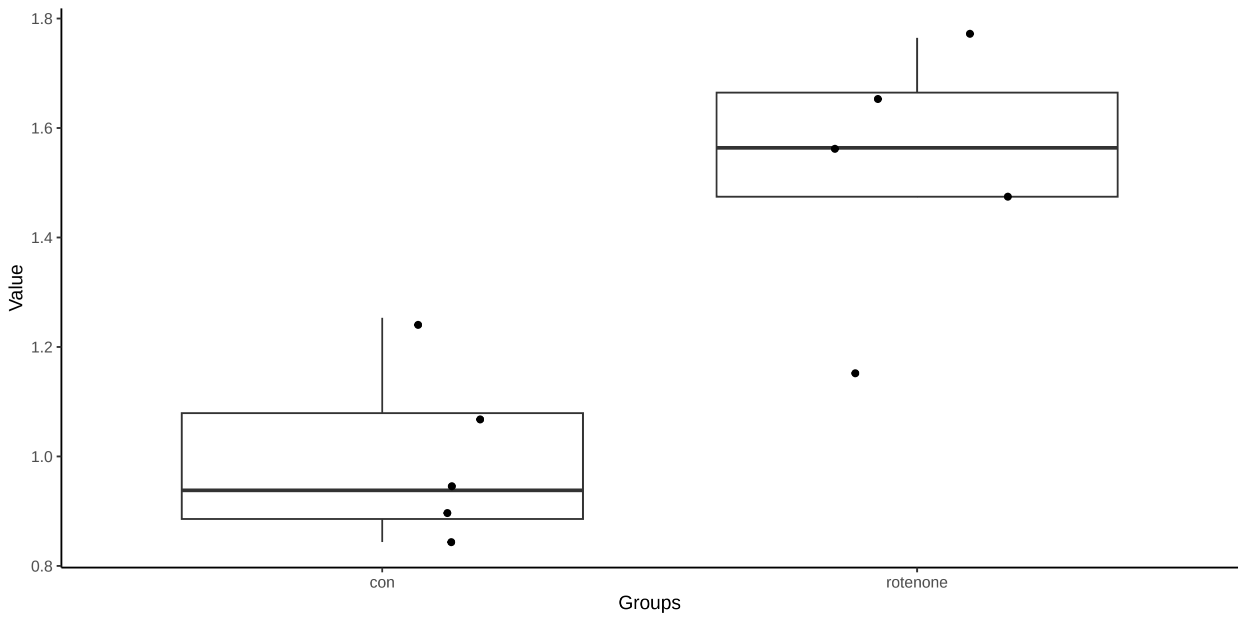


```
## 1. Normality assumption test by Shapiro_Wilk test is
##  p = 0.83
##  Normality assumption was not rejected
## 2. Equal variance test by Bartlett test is
##  p = 0.551
##  Equal variance assumption was not rejected
## 3. The result of anova is
##  p = 0.003
##  A statistically significant difference exist between groups
##
##   Tukey multiple comparisons of means
##     95% family-wise confidence level
##
```

```
## Fit: aov(formula = d1[, 3] ~ d1[, 2], data = d1)
##
## $`d1[, 2]`
##                   diff       lwr       upr    p adj
## rotenone-con 0.5263294 0.2332297 0.8194291 0.003249
```

Fig4_E.Western blot_rotenone_pro-IL-1beta_Data analysis

Boohwi Hong

```
## Present data is ** Western blot_rotenone_pro-IL-1beta.csv **
##
## **  Data structure  **
## 'data.frame':    10 obs. of  3 variables:
##  $ subject : int  1 2 3 4 5 6 7 8 9 10
##  $ group   : chr  "con" "con" "con" "con" ...
##  $ IL.1beta: num  0.626 0.689 0.93 1.409 1.346 ...
##
## ** Explorative data analysis with graphics**
```

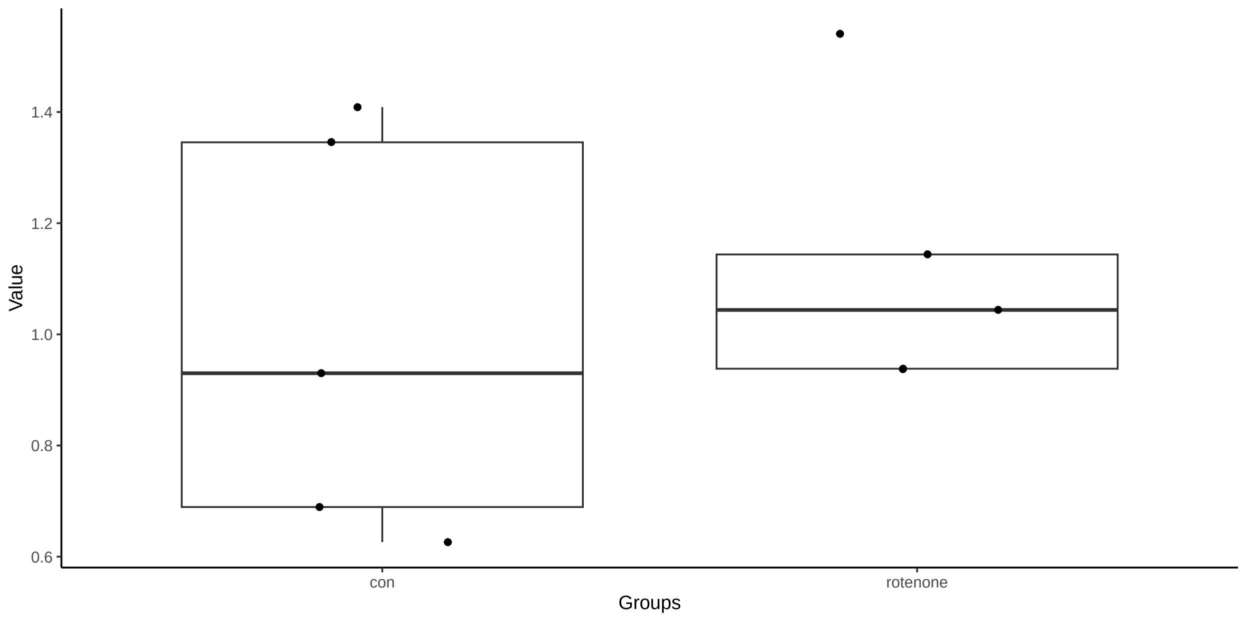


```
## 1. Normality assumption test by Shapiro_Wilk test is
##  p = 0.162
##  Normality assumption was not rejected
## 2. Equal variance test by Bartlett test is
##  p = 0.486
##  Equal variance assumption was not rejected
## 3. The result of anova is
##  p = 0.557
##  A statistically significant difference do not exist between groups
##
```

Fig4_E.Western blot_rotenone_cleaved-caspase1 Data analysis

Boohwi Hong

```
## Present data is ** Western blot_rotenone_cleaved-caspase1.csv **
##
## **  Data structure  **
## 'data.frame':    10 obs. of  3 variables:
##  $ subject          : int  1 2 3 4 5 6 7 8 9 10
##  $ group            : chr  "con" "con" "con" "con" ...
##  $ cleaved.caspase1: num  0.731 0.703 0.97 1.195 1.401 ...
##
## ** Explorative data analysis with graphics**
```

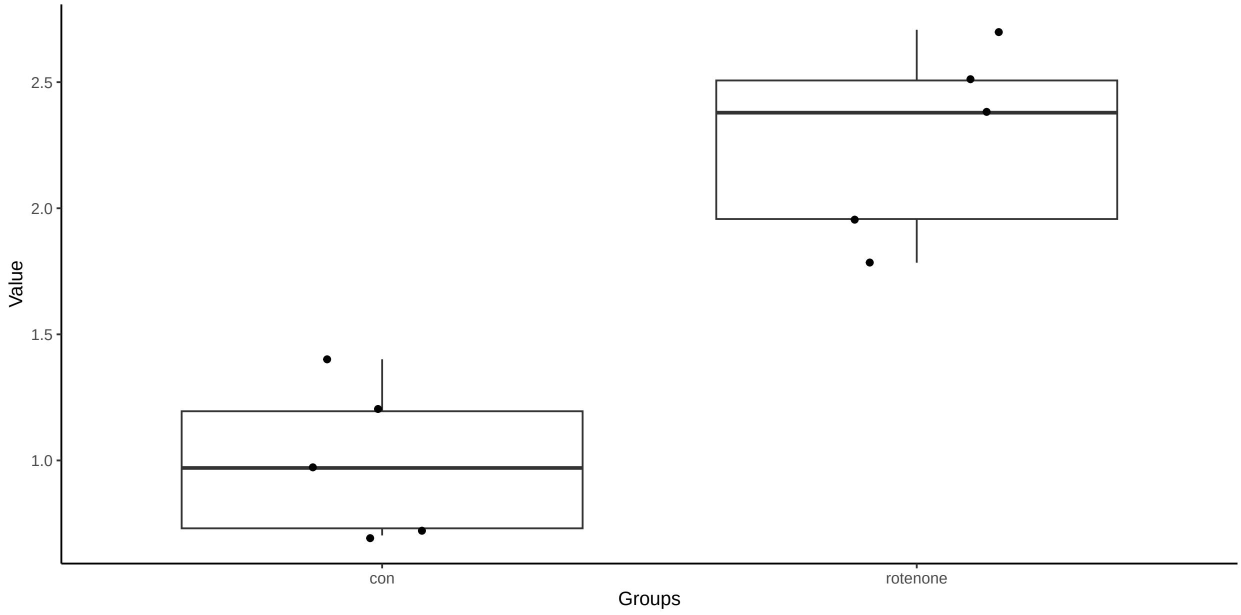


```
## 1. Normality assumption test by Shapiro_Wilk test is
##  p = 0.44
##  Normality assumption was not rejected
## 2. Equal variance test by Bartlett test is
##  p = 0.641
##  Equal variance assumption was not rejected
## 3. The result of anova is
##  p = 0.000
##  A statistically significant difference exist between groups
##
##   Tukey multiple comparisons of means
##     95% family-wise confidence level
##
```

```
## Fit: aov(formula = d1[, 3] ~ d1[, 2], data = d1)
##
## $`d1[, 2]`
##                  diff       lwr      upr     p adj
## rotenone-con 1.26687 0.7631849 1.770556 0.0004051
```

Barnes maze_primary latency Data analysis using R

Boohwi Hong

Package install

Data import

Data structure

```
str(dd)
```

```
## 'data.frame':    172 obs. of  4 variables:
##  $ subject: int  1 2 3 4 5 6 7 8 9 10 ...
##  $ group  : chr  "con" "con" "con" "con" ...
##  $ trial  : chr  "day1" "day1" "day1" "day1" ...
##  $ value  : num  49.7 57.1 38.3 45.9 96.6 ...
```

```
shapiro.test(dd$value)
```

```
##
##  Shapiro-Wilk normality test
##
## data:  dd$value
## W = 0.78515, p-value = 1.287e-14
```

Explorative data analysis with graphics

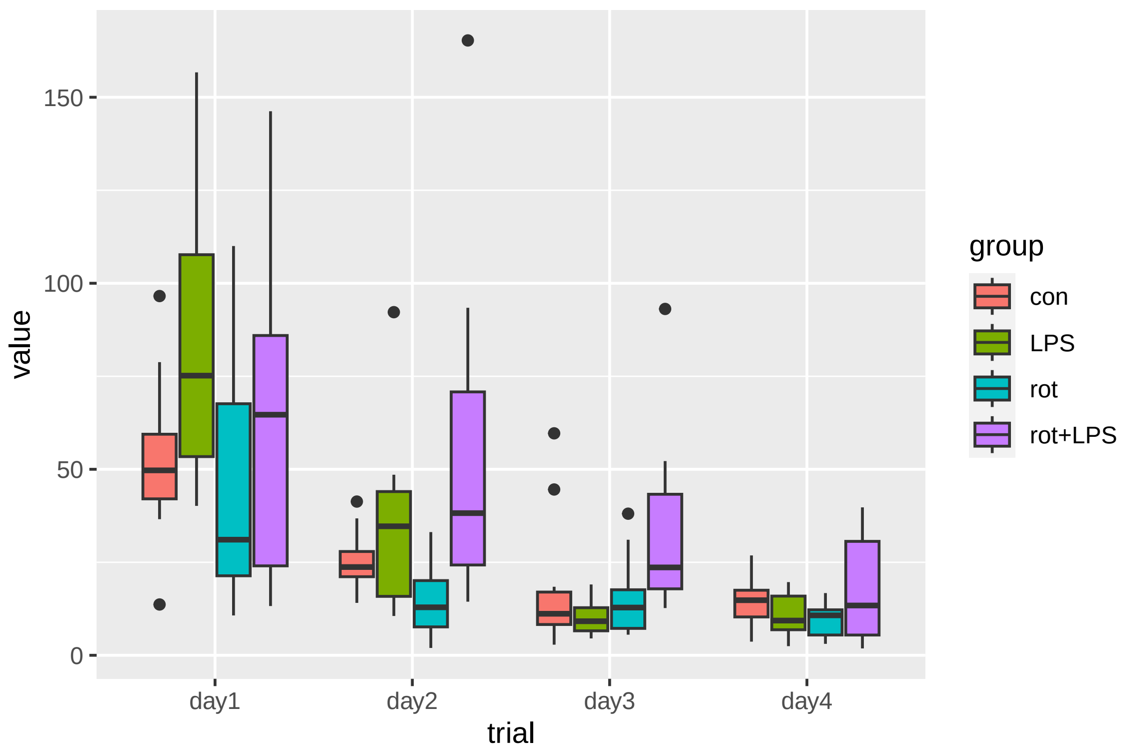

Model fit

```
##  F1 LD F1 Model
##  -----------------------
##  Check that the order of the time and group levels are correct.
##  Time level:   day1 day2 day3 day4
##  Group level:   con rot LPS rot+LPS
##  If the order is not correct, specify the correct order in time.order or group.order.

##              Statistic df      p-value
## group        17.51155  3 5.545962e-04
## trial       140.23799  3 3.358494e-30
## group:trial  39.25930  9 1.034406e-05

##              Statistic       df      p-value
## group        6.504124 2.563998 4.982697e-04
## trial       52.554569 2.568909 1.783804e-29
## group:trial  3.235050 6.376109 2.826849e-03

##        Statistic      df1      df2     p-value
## group  6.504124 2.563998 31.51396 0.002314232
```

Interpretation of result

```
## 1. Difference exist between groups
##  p = 0.000

## 2. Difference exist between measurement points
##  p = 0.000

## 3. Significant interaction exist between groups and measurement points
##  p = 0.003
```

Day 1

Explorative data analysis with graphics

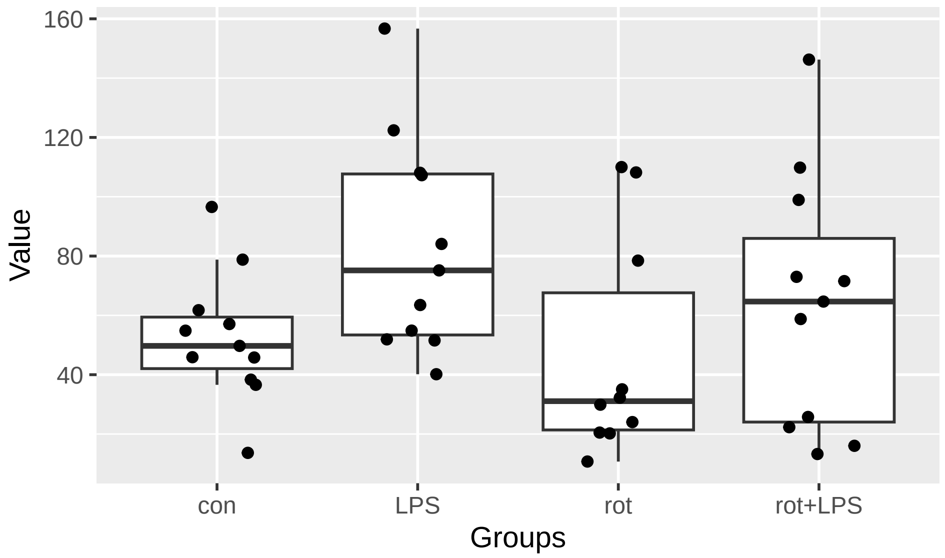

Result

```
## 1. Normality assumption test by Shapiro_Wilk test is
##  p = 0.047
##  Normality assumption was rejected
## 2. The result of Kruskall_Wallis test:
##  p = 0.101
##  A statistically significant difference do not exist between groups
```

Day 2

Explorative data analysis with graphics

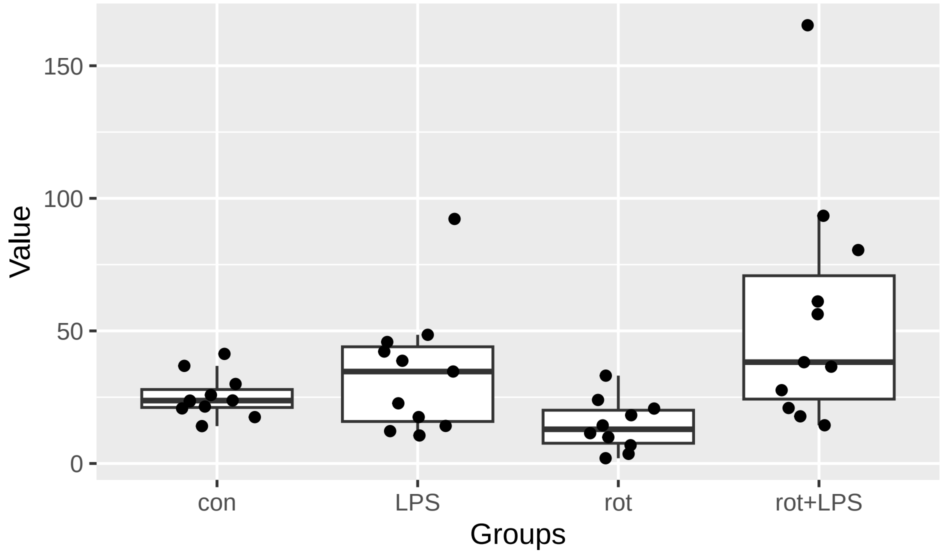

Result

```
## 1. Normality assumption test by Shapiro_Wilk test is
##  p = 0
##  Normality assumption was rejected
## 2. The result of Kruskall_Wallis test:
##  p = 0.005
##  A statistically significant difference exist between groups

## Dunn (1964) Kruskal-Wallis multiple comparison

##   p-values adjusted with the Benjamini-Hochberg method.

##      Comparison          Z      P.unadj       P.adj
## 1     con - LPS -0.4414607 0.6588794943 0.658879494
## 2     con - rot  1.9734941 0.0484392981 0.096878596
## 3     LPS - rot  2.4043156 0.0162027771 0.048608331
## 4 con - rot+LPS -1.5960503 0.1104775669 0.165716350
## 5 LPS - rot+LPS -1.1545896 0.2482585406 0.297910249
## 6 rot - rot+LPS -3.5310797 0.0004138671 0.002483202
```

Day 3

Explorative data analysis with graphics

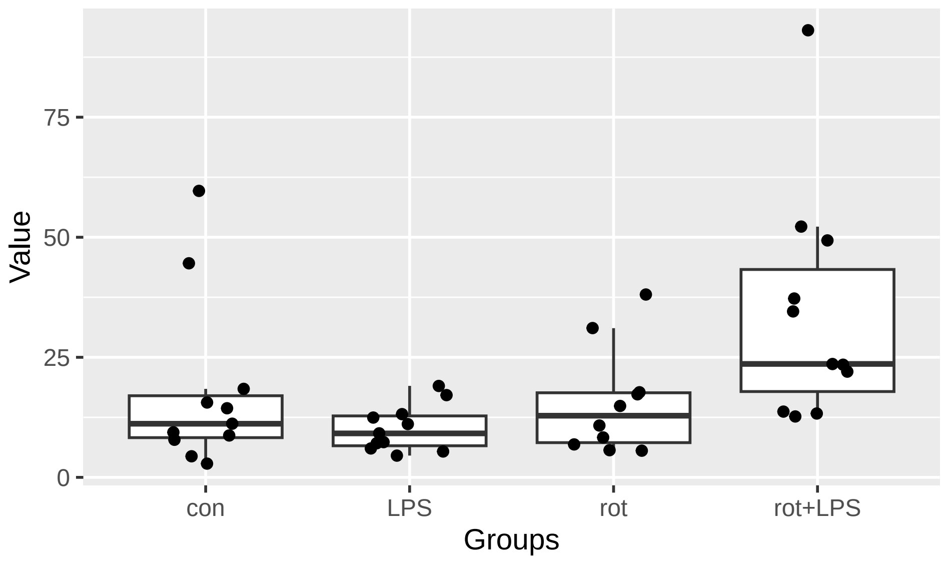

Result

```
## 1. Normality assumption test by Shapiro_Wilk test is
##  p = 0
##  Normality assumption was rejected
## 2. The result of Kruskall_Wallis test:
##  p = 0.006
##  A statistically significant difference exist between groups

## Dunn (1964) Kruskal-Wallis multiple comparison

##   p-values adjusted with the Benjamini-Hochberg method.

##      Comparison           Z      P.unadj       P.adj
## 1     con - LPS  1.00177624 0.3164516737 0.474677511
## 2     con - rot -0.01988407 0.9841358518 0.984135852
## 3     LPS - rot -0.99751758 0.3185133449 0.382216014
## 4 con - rot+LPS -2.37709617 0.0174495380 0.052348614
## 5 LPS - rot+LPS -3.37887241 0.0007278378 0.004367027
## 6 rot - rot+LPS -2.29992425 0.0214525117 0.042905023
```

Day 4

Explorative data analysis with graphics

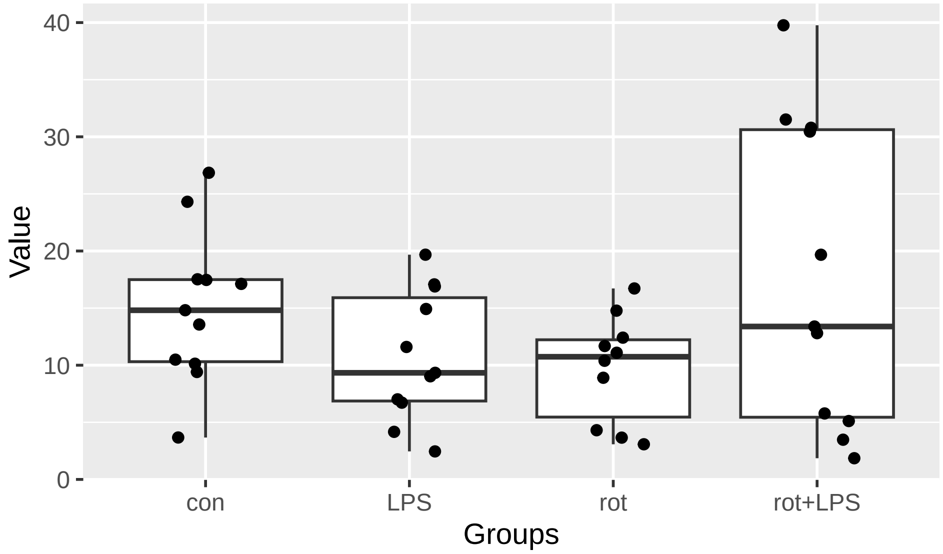

Result

```
## 1. Normality assumption test by Shapiro_Wilk test is
##  p = 0.757
##  Normality assumption was not rejected
## 2. Equal variance test by Bartlett test is
##  p = 0.004
##  Equal variance assumption was rejected
## 3. The result of Welch ANOVA is
##  p = 0.119
##  A statistically significant difference do not exist between groups
```

Fig4_F.Barnes maze primary length Data analysis using R

Boohwi Hong

Package install

Data import

Data structure

```
str(dd)
```

```
## 'data.frame':    172 obs. of  4 variables:
##  $ subject: int  1 2 3 4 5 6 7 8 9 10 ...
##  $ group  : chr  "con" "con" "con" "con" ...
##  $ trial  : chr  "day1" "day1" "day1" "day1" ...
##  $ value  : num  503 578 361 418 856 ...
```

```
shapiro.test(dd$value)
```

```
##
##  Shapiro-Wilk normality test
##
## data:  dd$value
## W = 0.84794, p-value = 4.277e-12
```

Explorative data analysis with graphics

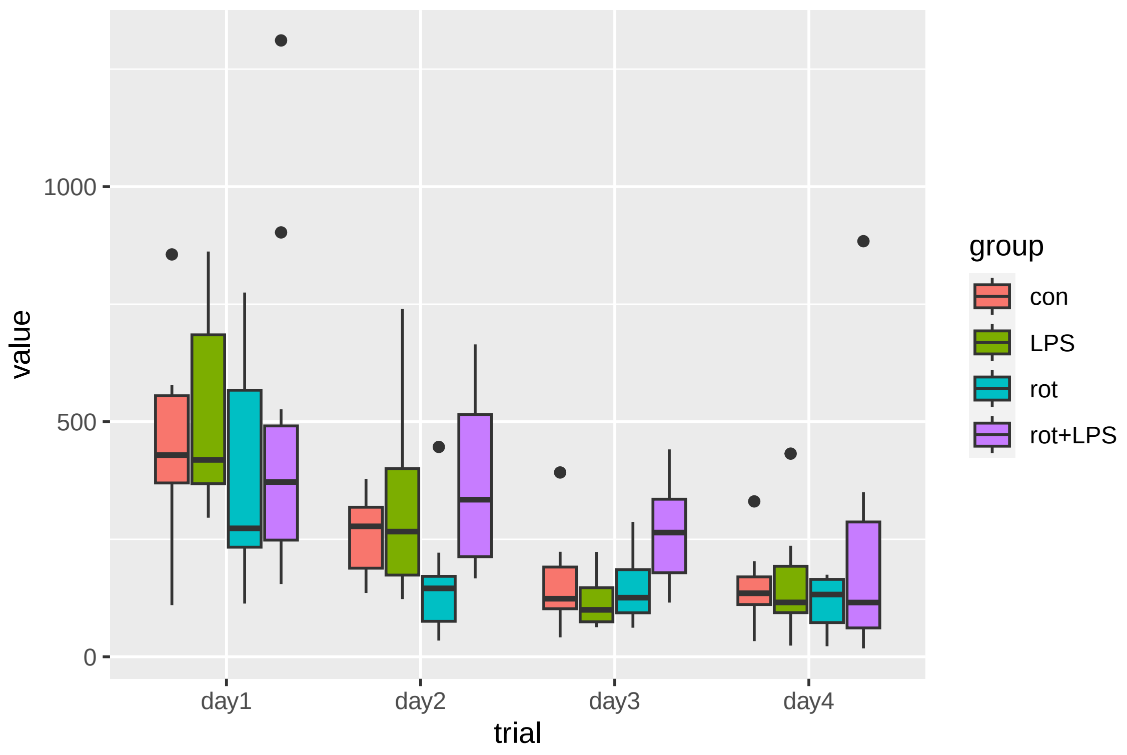

Model fit

```
##  F1 LD F1 Model
##  -----------------------
##  Check that the order of the time and group levels are correct.
##  Time level:   day1 day2 day3 day4
##  Group level:   con rot LPS rot+LPS
##  If the order is not correct, specify the correct order in time.order or group.order.

##              Statistic df      p-value
## group        22.64569  3 4.786603e-05
## trial        78.15374  3 7.638369e-17
## group:trial  43.74437  9 1.573046e-06

##              Statistic       df      p-value
## group        7.091432 2.865242 1.239577e-04
## trial       37.504403 2.614484 1.893424e-21
## group:trial  2.117680 6.297660 4.484102e-02

##        Statistic      df1     df2      p-value
## group  7.091432 2.865242 36.8894 0.0008215044
```

Interpretation of result

```
## 1. Difference exist between groups
##  p = 0.000

## 2. Difference exist between measurement points
##  p = 0.000

## 3. Significant interaction exist between groups and measurement points
##  p = 0.045
```

Day 1

Explorative data analysis with graphics

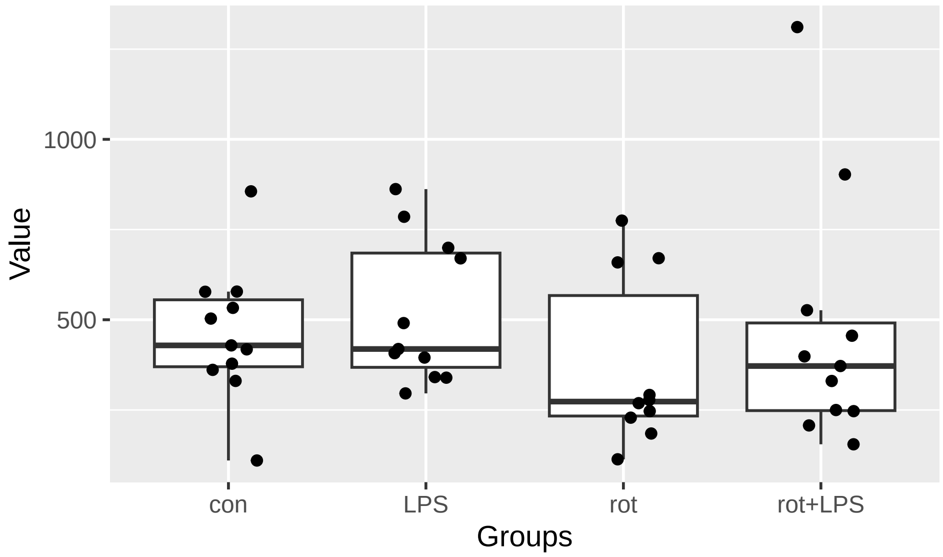

Result

```
## 1. Normality assumption test by Shapiro_Wilk test is
##  p = 0.001
##  Normality assumption was rejected
## 2. The result of Kruskall_Wallis test:
##  p = 0.212
##  A statistically significant difference do not exist between groups
```

Day 2

Explorative data analysis with graphics

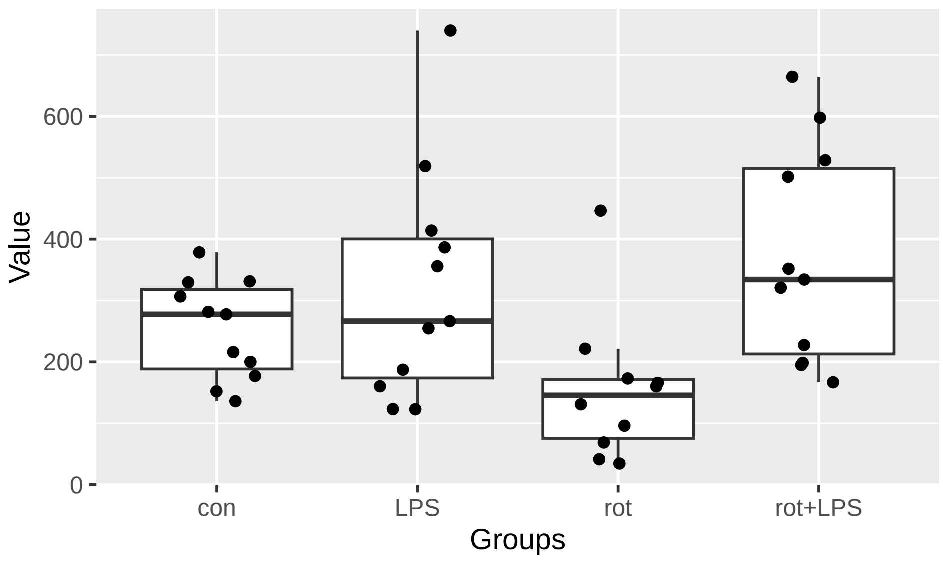

Result

```
## 1. Normality assumption test by Shapiro_Wilk test is
##  p = 0.044
##  Normality assumption was rejected
## 2. The result of Kruskall_Wallis test:
##  p = 0.008
##  A statistically significant difference exist between groups

## Dunn (1964) Kruskal-Wallis multiple comparison

##   p-values adjusted with the Benjamini-Hochberg method.

##      Comparison          Z      P.unadj       P.adj
## 1     con - LPS -0.4923985 0.6224376649 0.622437665
## 2     con - rot  1.9867501 0.0469500935 0.093900187
## 3     LPS - rot  2.4672819 0.0136143147 0.040842944
## 4 con - rot+LPS -1.3583407 0.1743556114 0.261533417
## 5 LPS - rot+LPS -0.8659422 0.3865218726 0.463826247
## 6 rot - rot+LPS -3.3123549 0.0009251411 0.005550846
```

Day 3

Explorative data analysis with graphics

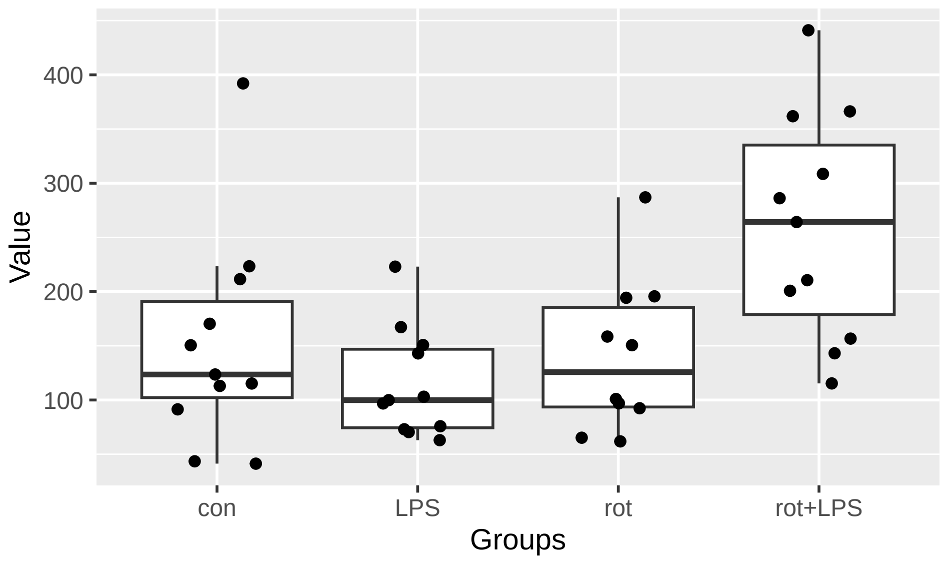

Result

```
## 1. Normality assumption test by Shapiro_Wilk test is
##  p = 0.11
##  Normality assumption was not rejected
## 2. Equal variance test by Bartlett test is
##  p = 0.123
##  Equal variance assumption was not rejected
## 3. The result of anova is
##  p = 0.001
##  A statistically significant difference exist between groups
```

```
##   Tukey multiple comparisons of means
##     95% family-wise confidence level
##
## Fit: aov(formula = d1[, 3] ~ d1[, 2], data = d1)
##
## $`d1[, 2]`
##                  diff        lwr       upr     p adj
## LPS-con     -37.25072 -133.74400  59.24257 0.7296070
## rot-con     -11.98749 -110.86368  86.88870 0.9879215
## rot+LPS-con 107.17708   10.68380 203.67037 0.0244320
## rot-LPS      25.26322  -73.61297 124.13941 0.9019594
## rot+LPS-LPS 144.42780   47.93452 240.92108 0.0014330
## rot+LPS-rot 119.16458   20.28839 218.04077 0.0127491
```

Day 4

Explorative data analysis with graphics

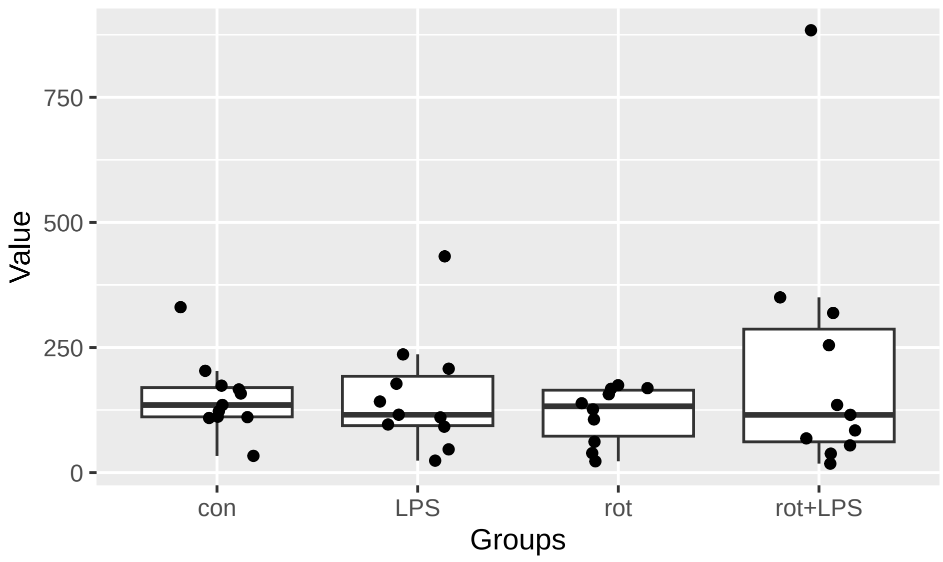

Result

```
## 1. Normality assumption test by Shapiro_Wilk test is
##  p = 0
##  Normality assumption was rejected
## 2. The result of Kruskall_Wallis test:
##  p = 0.913
##  A statistically significant difference do not exist between groups
```

Fig4_F.Barnes maze_primary errors Data analysis using R

Boohwi Hong

Package install

Data import

Data structure

```r
str(dd)
```

```
## 'data.frame':    172 obs. of  4 variables:
##  $ subject: int  1 2 3 4 5 6 7 8 9 10 ...
##  $ group  : chr  "con" "con" "con" "con" ...
##  $ trial  : chr  "day1" "day1" "day1" "day1" ...
##  $ value  : num  9.67 12 6.67 7 15.67 ...
```

```r
shapiro.test(dd$value)
```

```
##
##  Shapiro-Wilk normality test
##
## data:  dd$value
## W = 0.93412, p-value = 4.343e-07
```

Explorative data analysis with graphics

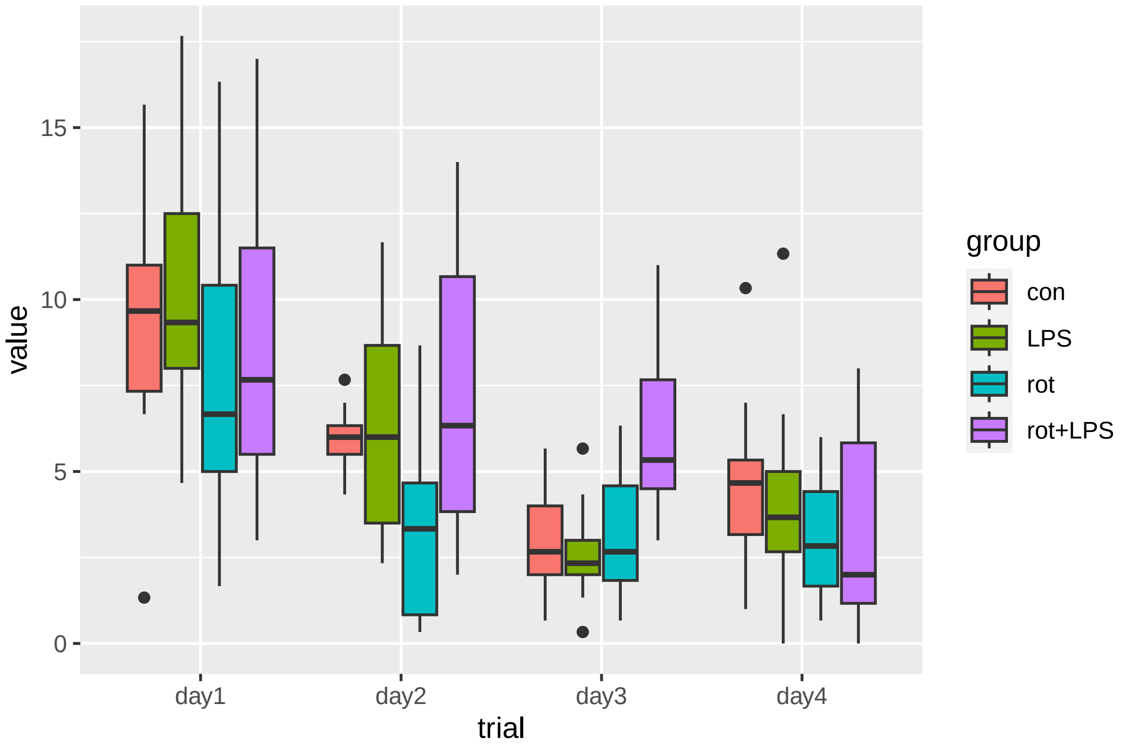

Model fit

```
##  F1 LD F1 Model
##  -----------------------
##  Check that the order of the time and group levels are correct.
##  Time level:   day1 day2 day3 day4
##  Group level:   con rot LPS rot+LPS
##  If the order is not correct, specify the correct order in time.order or group.order.

##              Statistic df      p-value
## group        14.53811  3 2.257102e-03
## trial        68.25298  3 1.009909e-14
## group:trial  34.79023  9 6.487847e-05

##              Statistic       df      p-value
## group        3.982147 2.481627 1.205689e-02
## trial       27.380497 2.769167 1.579823e-16
## group:trial  2.350883 6.998083 2.127205e-02

##       Statistic      df1      df2   p-value
## group  3.982147 2.481627 30.17444 0.0221079
```

Interpretation of result

```
## 1. Difference exist between groups
##  p = 0.012

## 2. Difference exist between measurement points
##  p = 0.000

## 3. Significant interaction exist between groups and measurement points
##  p = 0.021
```

Day 1

Explorative data analysis with graphics

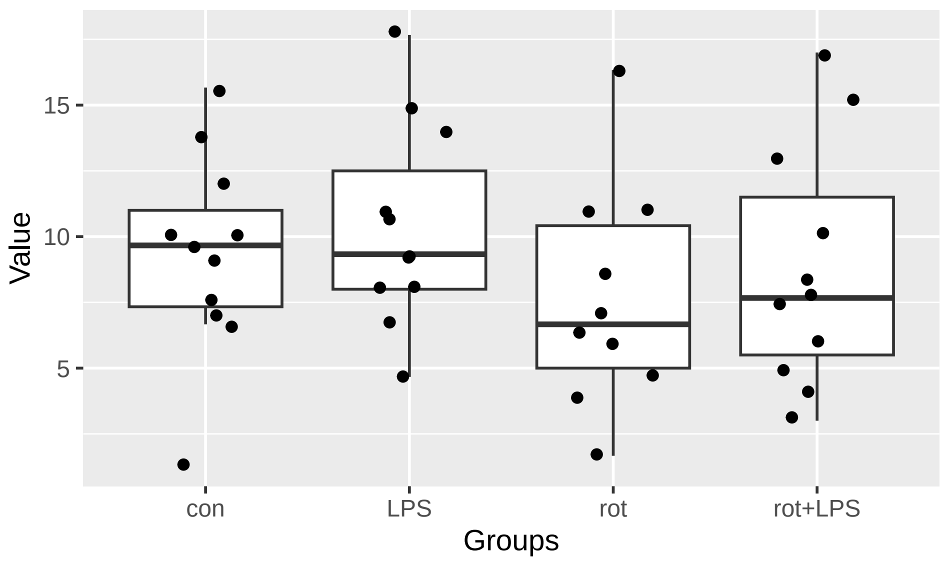

Result

```
## 1. Normality assumption test by Shapiro_Wilk test is
##  p = 0.347
##  Normality assumption was not rejected
## 2. Equal variance test by Bartlett test is
##  p = 0.930
##  Equal variance assumption was not rejected
## 3. The result of anova is
##  p = 0.5065
##  A statistically significant difference do not exist between groups
```

Day 2

Explorative data analysis with graphics

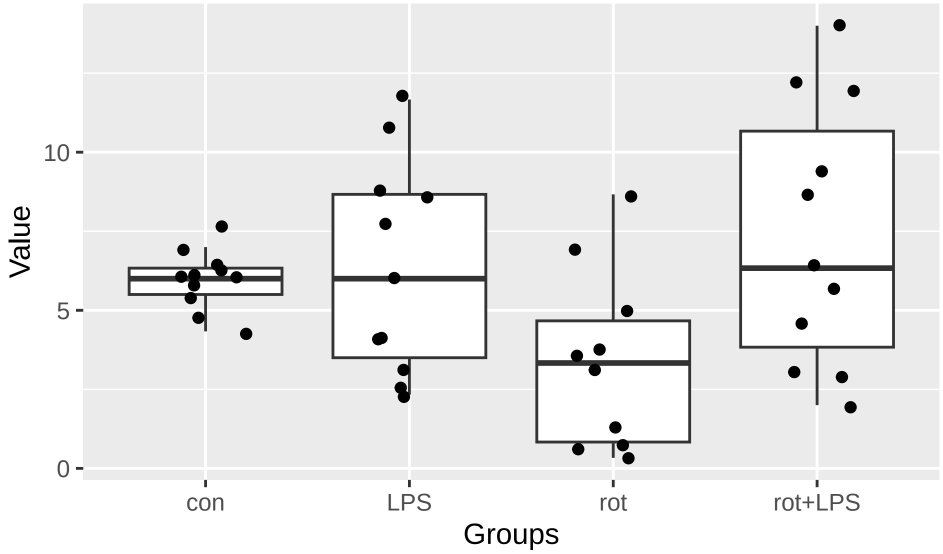

Result

```
## 1. Normality assumption test by Shapiro_Wilk test is
##  p = 0.365
##  Normality assumption was not rejected
## 2. Equal variance test by Bartlett test is
##  p = 0.001
##  Equal variance assumption was rejected
## 3. The result of Welch ANOVA is
##  p = 0.066
##  A statistically significant difference do not exist between groups
```

Day 3

Explorative data analysis with graphics

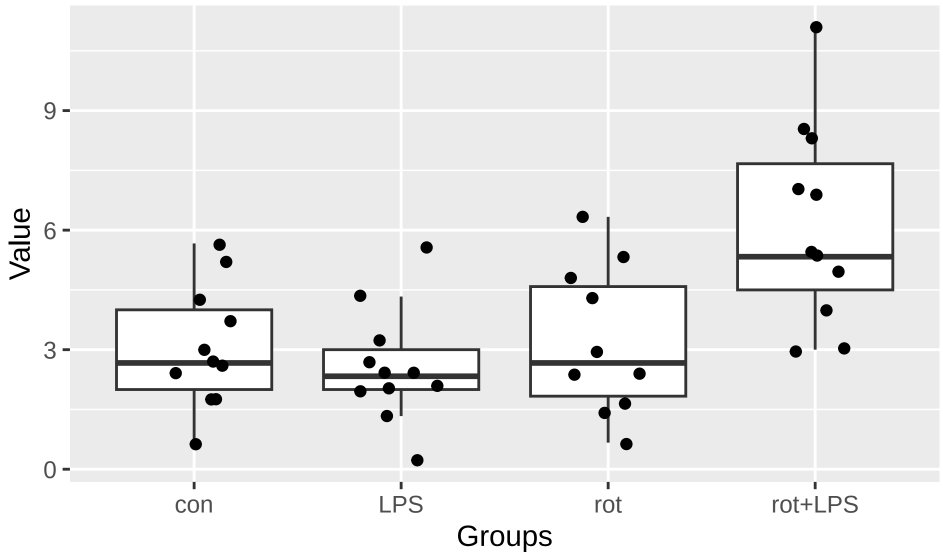

Result

```
## 1. Normality assumption test by Shapiro_Wilk test is
##  p = 0.347
##  Normality assumption was not rejected
## 2. Equal variance test by Bartlett test is
##  p = 0.298
##  Equal variance assumption was not rejected
## 3. The result of anova is
##  p = 0.000
##  A statistically significant difference exist between groups
```

```
##   Tukey multiple comparisons of means
##     95% family-wise confidence level
##
## Fit: aov(formula = d1[, 3] ~ d1[, 2], data = d1)
##
## $`d1[, 2]`
##                   diff        lwr      upr     p adj
## LPS-con     -0.4848485 -2.6535179 1.683821 0.9314656
## rot-con      0.1393939 -2.0828309 2.361619 0.9982743
## rot+LPS-con  3.0909091  0.9222397 5.259578 0.0025009
## rot-LPS      0.6242424 -1.5979824 2.846467 0.8744954
## rot+LPS-LPS  3.5757576  1.4070882 5.744427 0.0004239
## rot+LPS-rot  2.9515152  0.7292903 5.173740 0.0052201
```

Day 4

Explorative data analysis with graphics

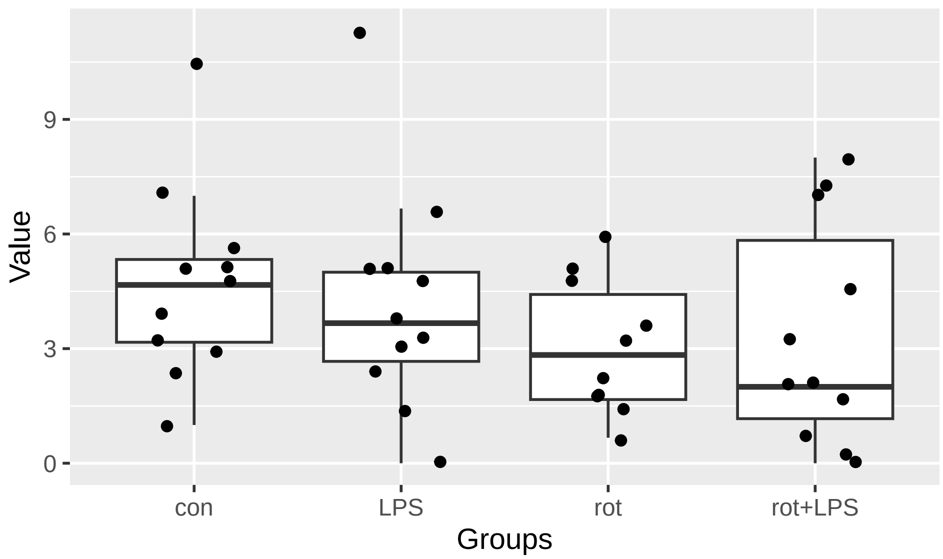

Result

```
## 1. Normality assumption test by Shapiro_Wilk test is
##  p = 0.09
##  Normality assumption was not rejected
## 2. Equal variance test by Bartlett test is
##  p = 0.435
##  Equal variance assumption was not rejected
## 3. The result of anova is
##  p = 0.4623
##  A statistically significant difference do not exist between groups
```

Fig4_H.Barnes maze_Probe test_duration_percent Data analysis

Boohwi Hong

Package install

Data import

Data structure

```
str(d1)
```

```
## 'data.frame':    43 obs. of  3 variables:
##  $ subject : int  1 2 3 4 5 6 7 8 9 10 ...
##  $ group   : chr  "con" "con" "con" "con" ...
##  $ Duration: num  16.9 51.7 26.2 37.3 41.5 ...
```

Explorative data analysis with graphics

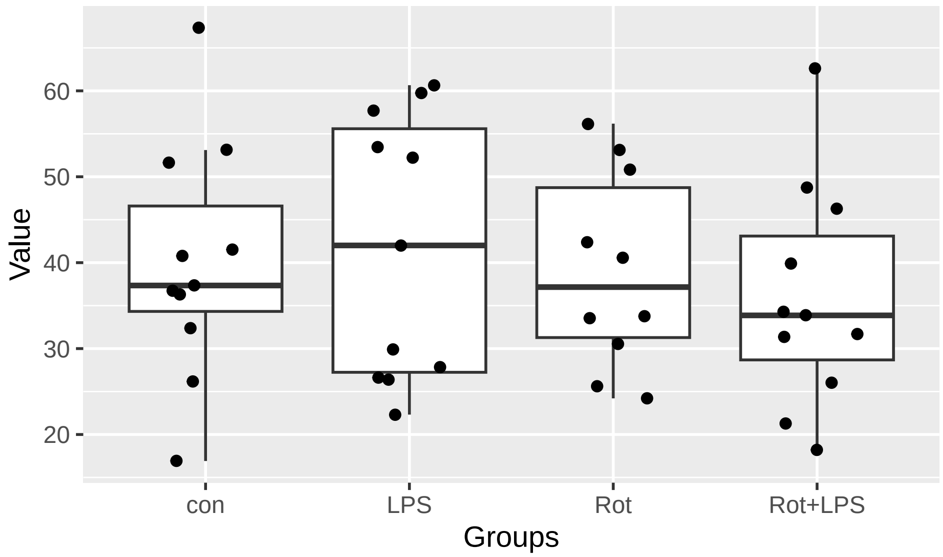

Easystat function developed by S. Park (available at https://rpubs.com/goodlebang)

Statistical Result

```r
easystat(d1)
```

```
## 1. Normality assumption test by Shapiro_Wilk test is
##  p = 0.185
##  Normality assumption was not rejected
## 2. Equal variance test by Bartlett test is
##  p = 0.846
##  Equal variance assumption was not rejected
## 3. The result of anova is
##  p = 0.7753
##  A statistically significant difference do not exist between groups
```

Fig4_H.Barnes maze_Probe test_path length Data analysis

Boohwi Hong

Package install

Data import

Data structure

```
str(d1)
```

```
## 'data.frame':    43 obs. of  3 variables:
##  $ subject : int  1 2 3 4 5 6 7 8 9 10 ...
##  $ group   : chr  "con" "con" "con" "con" ...
##  $ Duration: num  2.836 0.903 0.219 3.894 0.335 ...
```

Explorative data analysis with graphics

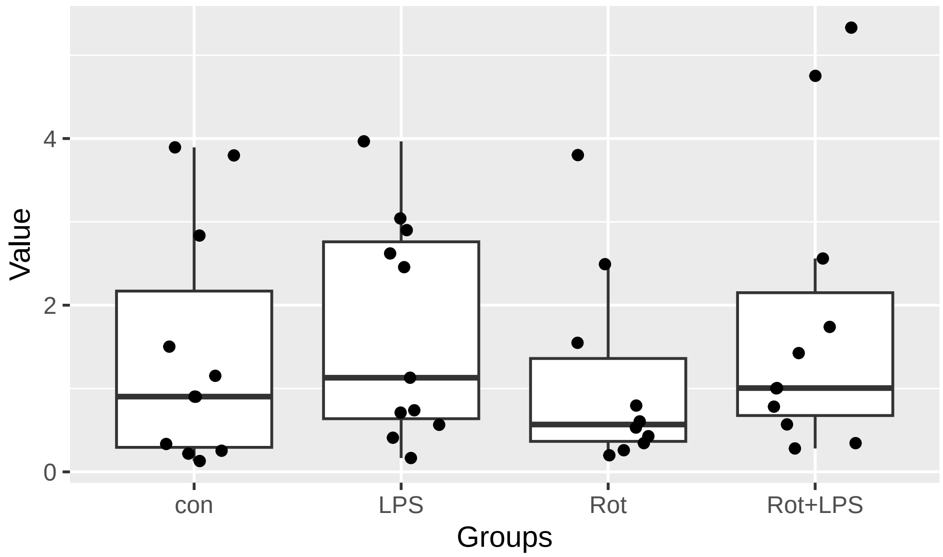

Easystat function developed by S. Park (available at https://rpubs.com/goodlebang)

Statistical Result

```r
easystat(d1)
```

```
## 1. Normality assumption test by Shapiro_Wilk test is
##  p = 0
##  Normality assumption was rejected
## 2. The result of Kruskall_Wallis test:
##  p = 0.577
##  A statistically significant difference do not exist between groups
```

Fig4_H.Barnes maze_Probe test_latency Data analysis

Boohwi Hong

Package install

Data import

Data structure

```
str(d1)
```

```
## 'data.frame':    43 obs. of  3 variables:
##  $ subject: int  1 2 3 4 5 6 7 8 9 10 ...
##  $ group  : chr  "con" "con" "con" "con" ...
##  $ Latency: num  34.5 7.03 0.6 43.2 1.67 ...
```

Explorative data analysis with graphics

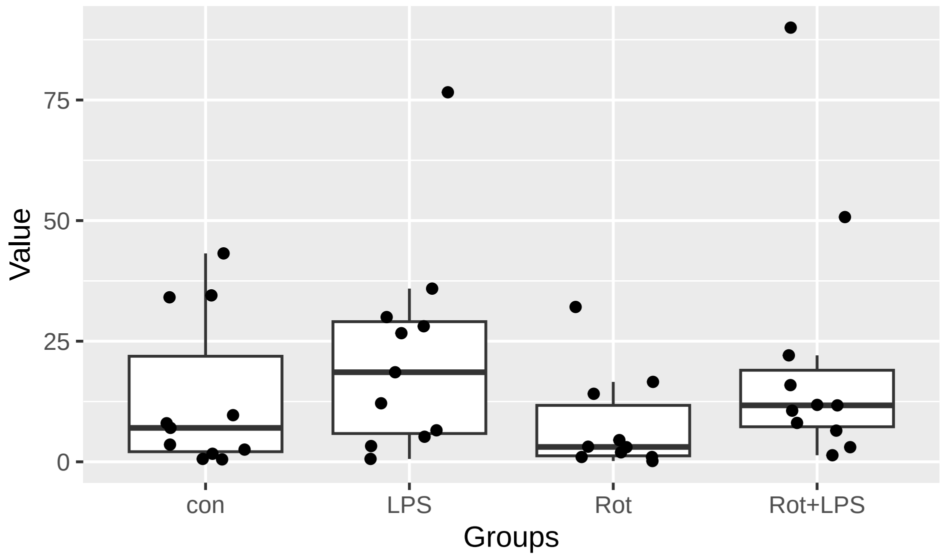

Easystat function developed by S. Park (available at https://rpubs.com/goodlebang)

Statistical Result

```
easystat(d1)
```

```
## 1. Normality assumption test by Shapiro_Wilk test is
##  p = 0
##  Normality assumption was rejected
## 2. The result of Kruskall_Wallis test:
##  p = 0.148
##  A statistically significant difference do not exist between groups
```

Fig4_I.Fear chamber_freezing_percent Data analysis

Boohwi Hong

Package install

Data import

Data structure

```r
str(d1)
```

```
## 'data.frame':    43 obs. of  3 variables:
##  $ subject : int  1 2 3 4 5 6 7 8 9 10 ...
##  $ group   : chr  "con" "con" "con" "con" ...
##  $ freezing: num  85.7 60 87.2 66.4 73.9 ...
```

Explorative data analysis with graphics

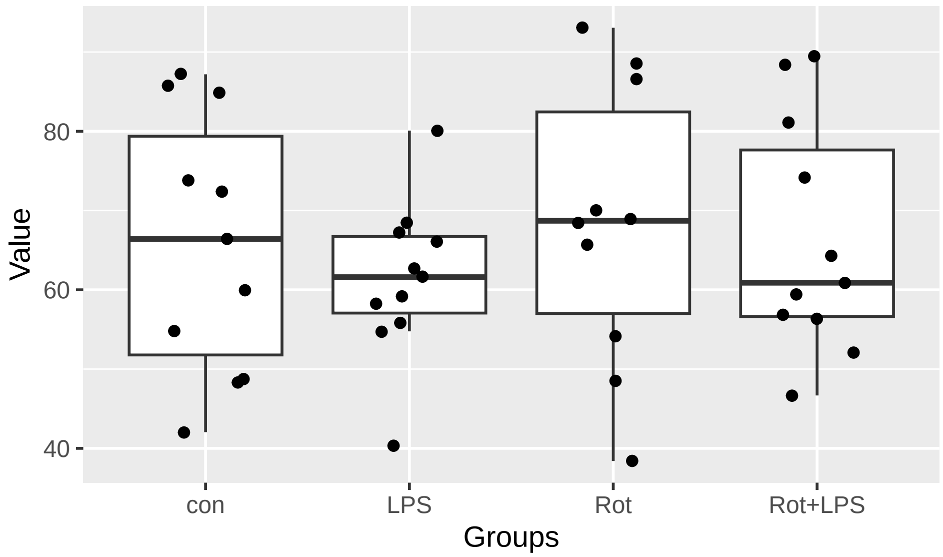

Easystat function developed by S. Park (available at https://rpubs.com/goodlebang)

Statistical Result

```
easystat(d1)
```

```
## 1. Normality assumption test by Shapiro_Wilk test is
##  p = 0.236
##  Normality assumption was not rejected
## 2. Equal variance test by Bartlett test is
##  p = 0.363
##  Equal variance assumption was not rejected
## 3. The result of anova is
##  p = 0.7439
##  A statistically significant difference do not exist between groups
```

Figure5 Statistics

Fig5_B.LPS post12days_OCR_Basal Data analysis

Data structure

```
str(d1)
```

```
## 'data.frame':    11 obs. of  4 variables:
##  $ subject: int  1 2 3 4 5 6 7 8 9 10 ...
##  $ group  : chr  "con" "con" "con" "con" ...
##  $ OCR    : num  189 250 195 185 208 ...
##  $ X      : logi  NA NA NA NA NA NA ...
```

Explorative data analysis with graphics

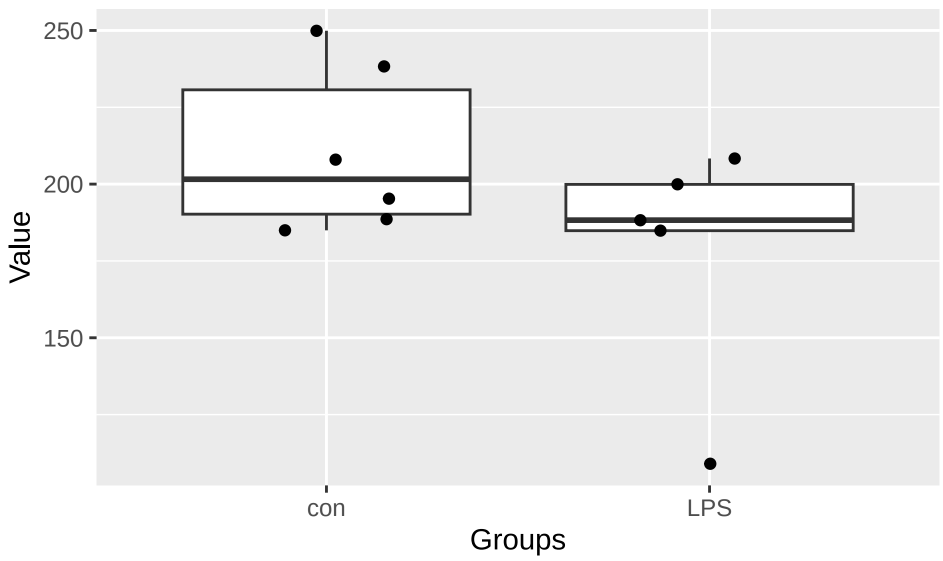


```
##
##  Welch Two Sample t-test
##
## data:  value by group
## t = 1.5625, df = 6.8941, p-value = 0.1628
## alternative hypothesis: true difference in means between group con and group LPS is not equal to 0
## 95 percent confidence interval:
##  -16.95547  82.40801
## sample estimates:
## mean in group con mean in group LPS
##          210.8103          178.0841
```

```
## The result of t-test:
##  p = 0.163
##  A statistically significant difference do not exist between groups
```

Fig5_B.LPS post 12days_OCR_State3 Data analysis

Data structure

```
str(d1)
```

```
## 'data.frame':    11 obs. of  4 variables:
##  $ subject: int  1 2 3 4 5 6 7 8 9 10 ...
##  $ group  : chr  "con" "con" "con" "con" ...
##  $ OCR    : num  591 750 634 625 465 ...
##  $ X      : logi  NA NA NA NA NA NA ...
```

Explorative data analysis with graphics

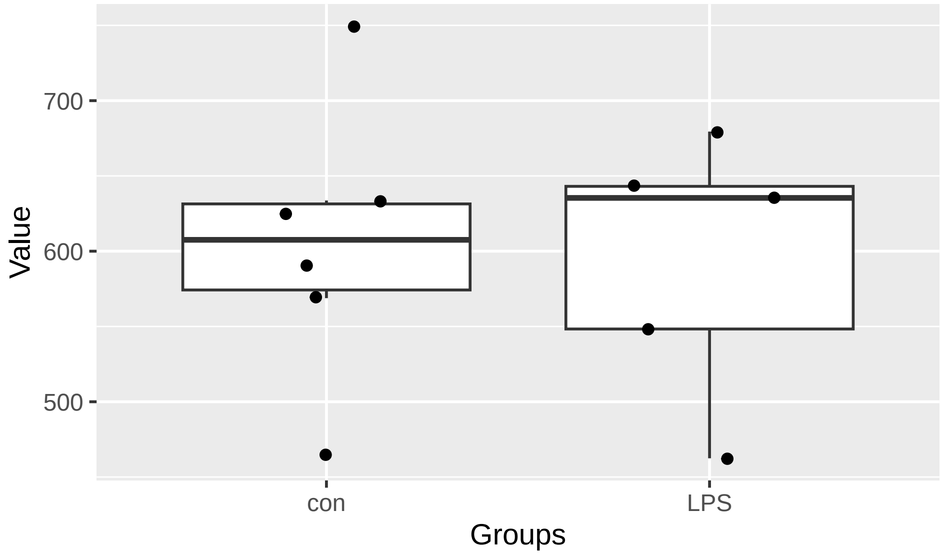


```
##
##  Welch Two Sample t-test
##
## data:  value by group
## t = 0.21207, df = 8.8176, p-value = 0.8369
## alternative hypothesis: true difference in means between group con and group LPS is not equal to 0
## 95 percent confidence interval:
##  -112.4321  135.6123
## sample estimates:
## mean in group con mean in group LPS
##          605.3309          593.7408
```

```
## The result of t-test:
##  p = 0.837
##  A statistically significant difference do not exist between groups
```

Fig5_B.LPS post 12days_OCR_State4o Data analysis

Data structure

```
str(d1)
```

```
## 'data.frame':    11 obs. of  4 variables:
##  $ subject: int  1 2 3 4 5 6 7 8 9 10 ...
##  $ group  : chr  "con" "con" "con" "con" ...
##  $ OCR    : num  147 206 142 128 106 ...
##  $ X      : logi  NA NA NA NA NA NA ...
```

Explorative data analysis with graphics

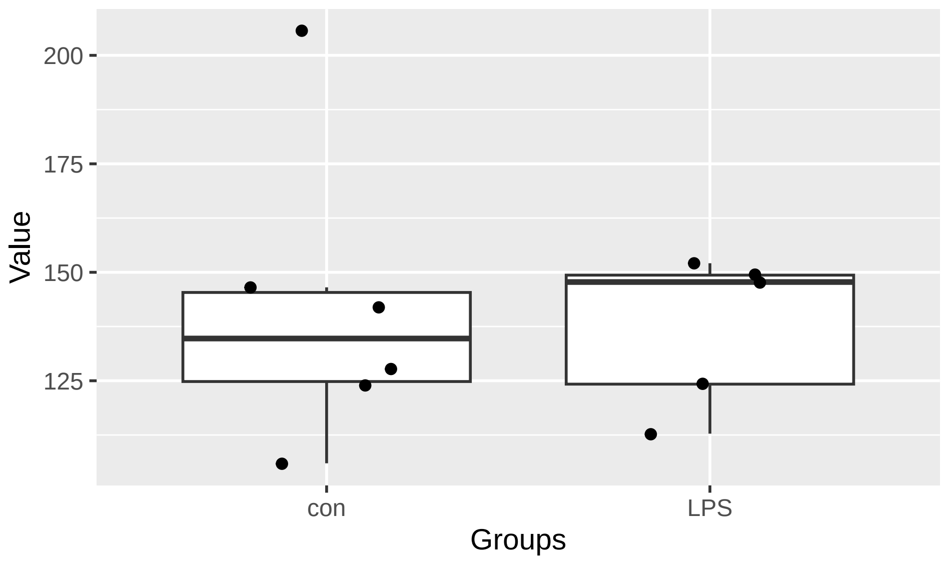


```
##
##  Welch Two Sample t-test
##
## data:  value by group
## t = 0.29025, df = 7.7, p-value = 0.7793
## alternative hypothesis: true difference in means between group con and group LPS is not equal to 0
## 95 percent confidence interval:
##  -32.68473  42.02457
## sample estimates:
## mean in group con mean in group LPS
##          141.9135          137.2436
```

```
## The result of t-test:
##  p = 0.779
##  A statistically significant difference do not exist between groups
```

Fig5_B.LPS post 12days_OCR_State3u Data analysis

Data structure

```
str(d1)
```

```
## 'data.frame':    11 obs. of  4 variables:
##  $ subject: int  1 2 3 4 5 6 7 8 9 10 ...
##  $ group  : chr  "con" "con" "con" "con" ...
##  $ OCR    : num  491 702 574 499 268 ...
##  $ X      : logi  NA NA NA NA NA NA ...
```

Explorative data analysis with graphics

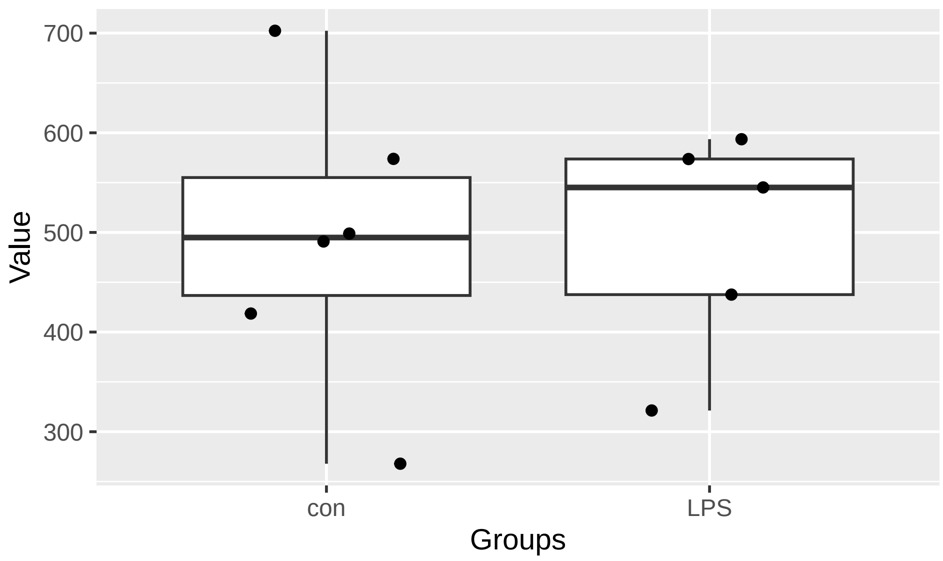


```
##
##  Welch Two Sample t-test
##
## data:  value by group
## t = -0.027627, df = 8.9816, p-value = 0.9786
## alternative hypothesis: true difference in means between group con and group LPS is not equal to 0
## 95 percent confidence interval:
##  -179.6095  175.2767
## sample estimates:
## mean in group con mean in group LPS
##          492.0783          494.2447
```

```
## The result of t-test:
##  p = 0.979
##  A statistically significant difference do not exist between groups
```

Fig5_C.LPS post 12days_RCR Data analysis

Data structure

```
str(d1)
```

```
## 'data.frame':    11 obs. of  3 variables:
##  $ subject: int  1 2 3 4 5 6 7 8 9 10 ...
##  $ group  : chr  "con" "con" "con" "con" ...
##  $ RCR    : num  2.84 2.57 3.02 3.35 2.56 ...
```

Explorative data analysis with graphics

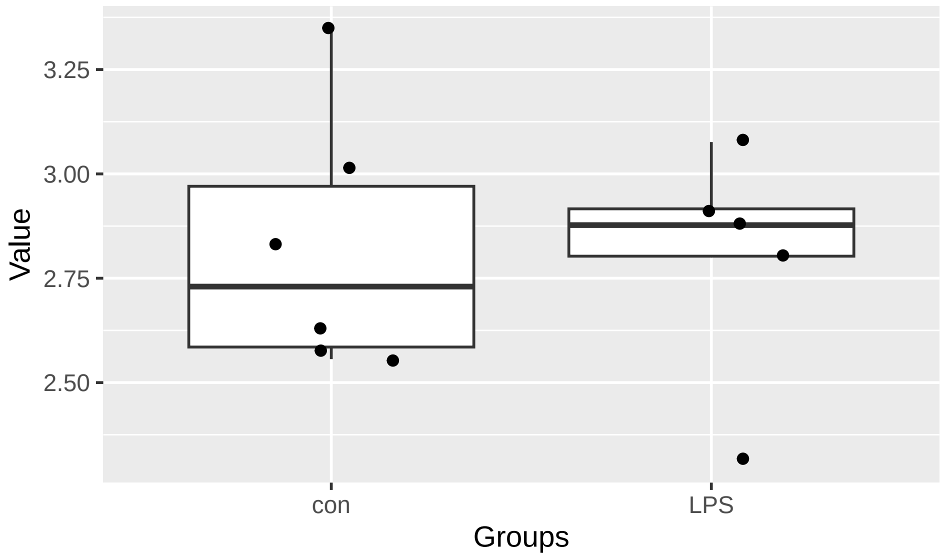


```
##
##  Welch Two Sample t-test
##
## data:  value by group
## t = 0.15724, df = 8.8622, p-value = 0.8786
## alternative hypothesis: true difference in means between group con and group LPS is not equal to 0
## 95 percent confidence interval:
##  -0.3830683  0.4401533
## sample estimates:
## mean in group con mean in group LPS
##          2.825541          2.796999
```

```
## The result of t-test:
##  p = 0.879
##  A statistically significant difference do not exist between groups
```

Fig5_C.LPS post 12days_RCRu Data analysis

Data structure

```
str(d1)
```

```
## 'data.frame':    11 obs. of  4 variables:
##  $ subject: int  1 2 3 4 5 6 7 8 9 10 ...
##  $ group  : chr  "con" "con" "con" "con" ...
##  $ RCRu   : num  2.42 2.44 2.77 2.76 1.7 ...
##  $ X      : logi  NA NA NA NA NA NA ...
```

Explorative data analysis with graphics

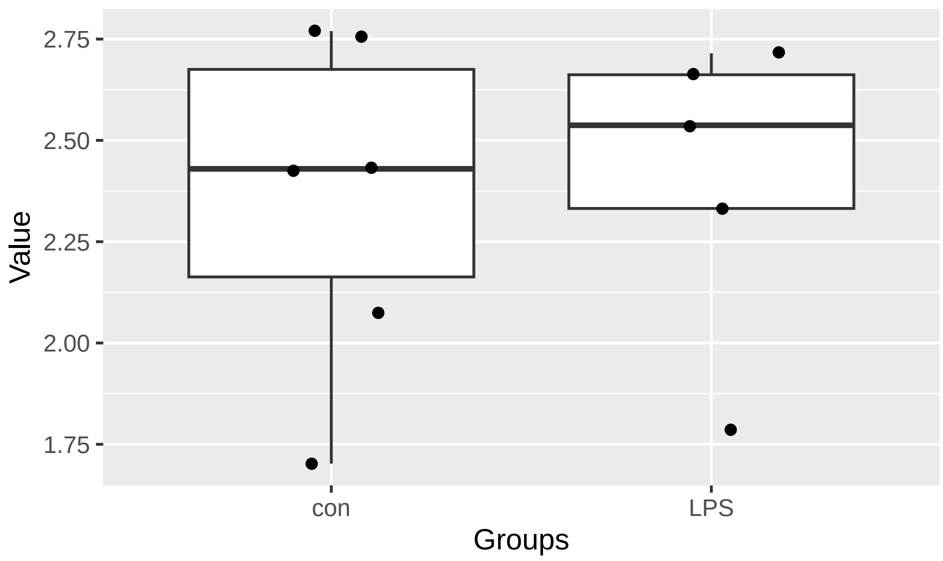


```
##
##  Welch Two Sample t-test
##
## data:  value by group
## t = -0.18985, df = 8.8747, p-value = 0.8537
## alternative hypothesis: true difference in means between group con and group LPS is not equal to 0
## 95 percent confidence interval:
##  -0.5858532  0.4953106
## sample estimates:
## mean in group con mean in group LPS
##          2.360459          2.405730
```

```
## The result of t-test:
##  p = 0.854
##  A statistically significant difference do not exist between groups
```

Fig5_D.LPS post12days_Western Blot_ATP5A Data analysis

Data structure

```
str(d1)
```

```
## 'data.frame':    11 obs. of  3 variables:
##  $ subject: int  1 2 3 4 5 6 7 8 9 10 ...
##  $ group  : chr  "con" "con" "con" "con" ...
##  $ ATP5A  : num  1.499 0.915 0.794 0.847 0.945 ...
```

Explorative data analysis with graphics

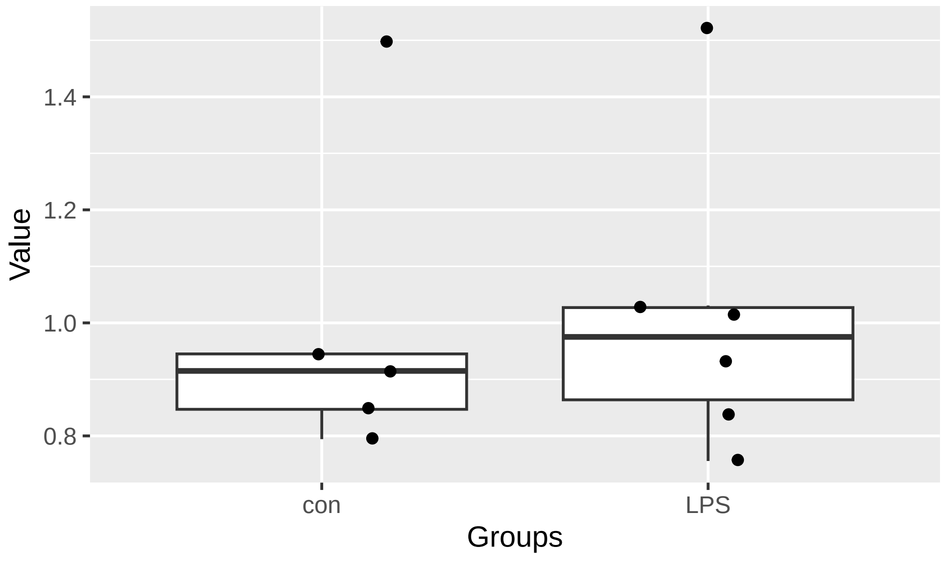


```
##
##  Welch Two Sample t-test
##
## data:  value by group
## t = -0.096501, df = 8.4178, p-value = 0.9254
## alternative hypothesis: true difference in means between group con and group LPS is not equal to 0
## 95 percent confidence interval:
##  -0.4003083  0.3678833
## sample estimates:
## mean in group con mean in group LPS
##          1.000000          1.016212
```

```
## The result of t-test:
##  p = 0.925
##  A statistically significant difference do not exist between groups
```

Fig5_D.LPS post12days_Western Blot_UQCRC2 Data analysis

Data structure

```
str(d1)
```

```
## 'data.frame':    11 obs. of  3 variables:
##  $ subject: int  1 2 3 4 5 6 7 8 9 10 ...
##  $ group  : chr  "con" "con" "con" "con" ...
##  $ UQCRC2 : num  1.442 0.962 0.75 0.903 0.944 ...
```

Explorative data analysis with graphics

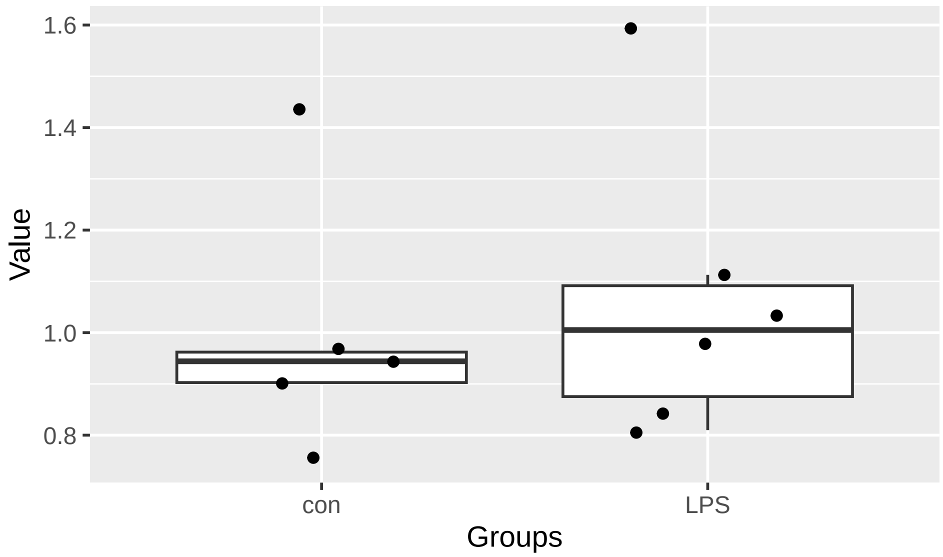


```
##
##  Welch Two Sample t-test
##
## data:  value by group
## t = -0.37111, df = 8.8859, p-value = 0.7192
## alternative hypothesis: true difference in means between group con and group LPS is not equal to 0
## 95 percent confidence interval:
##  -0.4344562  0.3122043
## sample estimates:
## mean in group con mean in group LPS
##          1.000000          1.061126
```

```
## The result of t-test:
##  p = 0.719
##  A statistically significant difference do not exist between groups
```

Fig5_D.LPS post12days_Western Blot_SDHB Data analysis

Data structure

```
str(d1)
```

```
## 'data.frame':    11 obs. of  3 variables:
##  $ subject: int  1 2 3 4 5 6 7 8 9 10 ...
##  $ group  : chr  "con" "con" "con" "con" ...
##  $ SDHB   : num  1.243 1.006 0.857 0.863 1.032 ...
```

Explorative data analysis with graphics

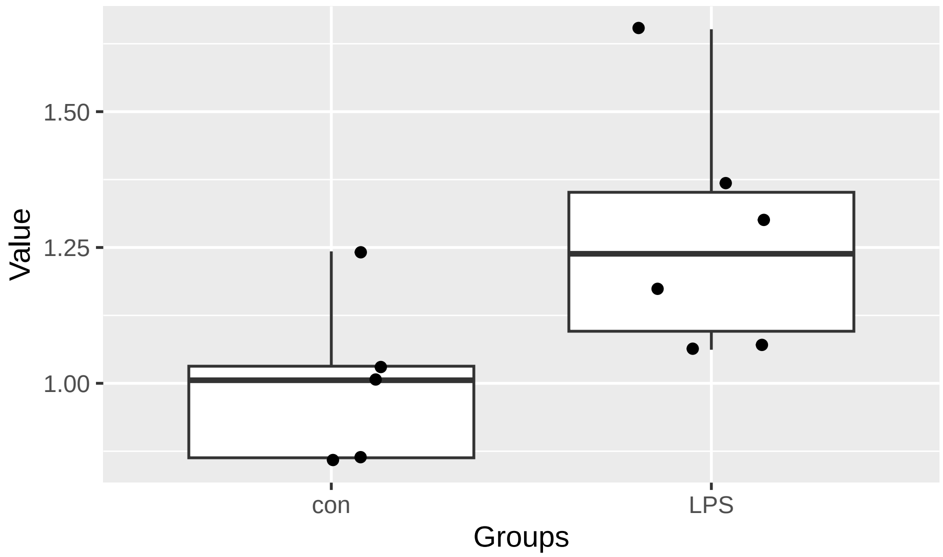


```
##
##  Welch Two Sample t-test
##
## data:  value by group
## t = -2.3563, df = 8.8241, p-value = 0.04339
## alternative hypothesis: true difference in means between group con and group LPS is not equal to 0
## 95 percent confidence interval:
##  -0.53256966 -0.01004734
## sample estimates:
## mean in group con mean in group LPS
##          1.000000          1.271308
```

```
## The result of t-test:
##  p = 0.043
##  A statistically significant difference exist between groups
```

Fig5_D.LPS post12days_Western Blot_NDUFB8 Data analysis

Data structure

```
str(d1)
```

```
## 'data.frame':    11 obs. of  3 variables:
##  $ subject: int  1 2 3 4 5 6 7 8 9 10 ...
##  $ group  : chr  "con" "con" "con" "con" ...
##  $ NDUFB8 : num  0.947 1.073 0.915 1.076 0.99 ...
```

Explorative data analysis with graphics

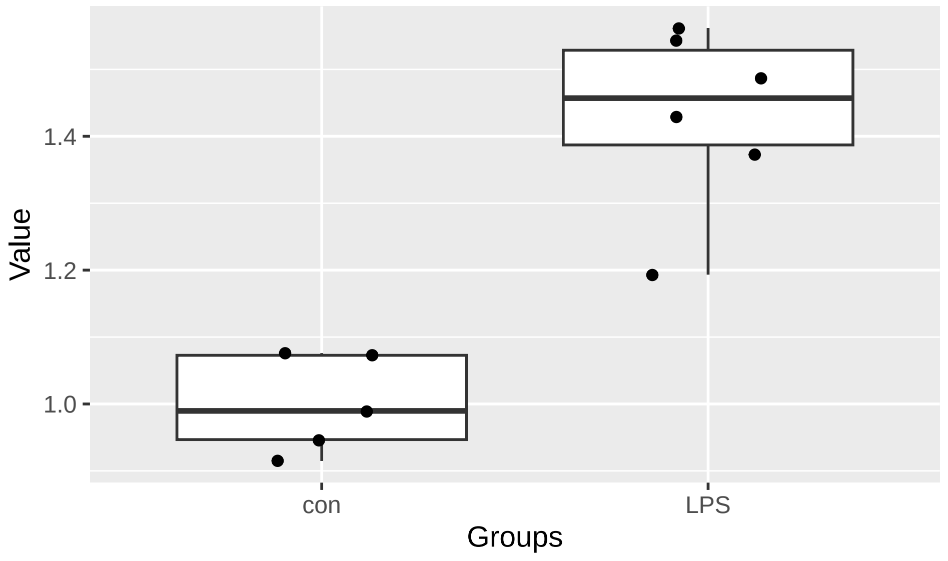


```
##
##  Welch Two Sample t-test
##
## data:  value by group
## t = -6.6908, df = 7.8733, p-value = 0.0001657
## alternative hypothesis: true difference in means between group con and group LPS is not equal to 0
## 95 percent confidence interval:
##  -0.5798256 -0.2819721
## sample estimates:
## mean in group con mean in group LPS
##          1.000000          1.430899
```

```
## The result of t-test:
##  p = 0
##  A statistically significant difference exist between groups
```

Fig5_E.LPS post12days_qPCR_TNFalpha Data analysis

Data structure

```
str(d1)
```

```
## 'data.frame':    11 obs. of  3 variables:
##  $ subject : int  1 2 3 4 5 6 7 8 9 10 ...
##  $ group   : chr  "con" "con" "con" "con" ...
##  $ TNFalpha: num  0.0408 0.9734 1.1966 1.6939 1.0954 ...
```

Explorative data analysis with graphics

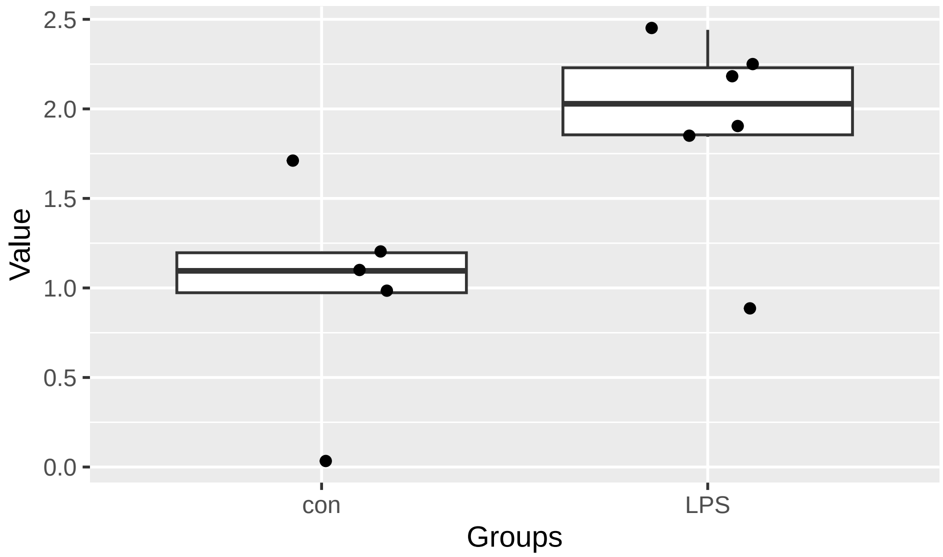


```
##
##  Welch Two Sample t-test
##
## data:  value by group
## t = -2.5837, df = 8.3429, p-value = 0.03134
## alternative hypothesis: true difference in means between group con and group LPS is not equal to 0
## 95 percent confidence interval:
##  -1.7172062 -0.1036434
## sample estimates:
## mean in group con mean in group LPS
##          1.000000          1.910425
```

```
## The result of t-test:
##  p = 0.031
##  A statistically significant difference exist between groups
```

Fig5_E.LPS post12days_qPCR_IL-1beta Data analysis

Data structure

```
str(d1)
```

```
## 'data.frame':    11 obs. of  3 variables:
##  $ subject : int  1 2 3 4 5 6 7 8 9 10 ...
##  $ group   : chr  "con" "con" "con" "con" ...
##  $ IL.1beta: num  0.0496 0.6713 1.2766 1.9053 1.0972 ...
```

Explorative data analysis with graphics

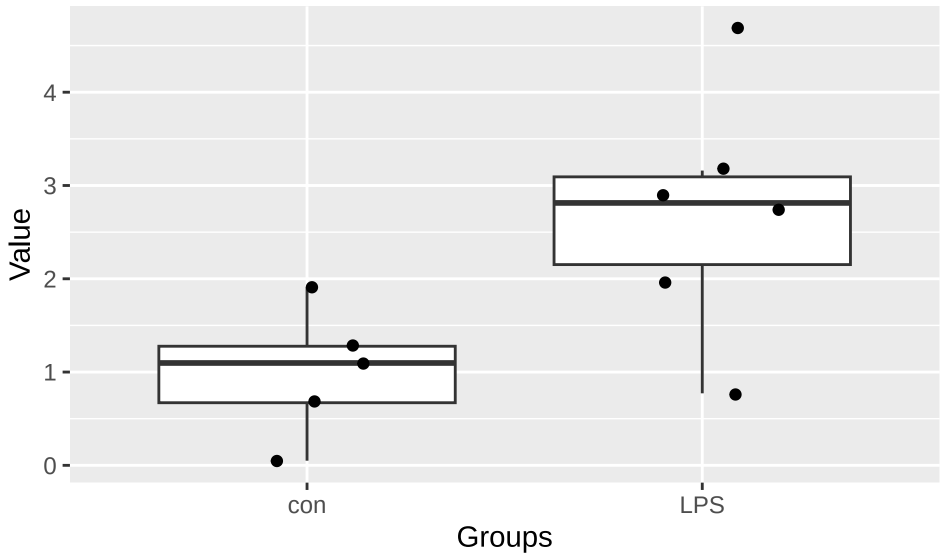


```
##
##  Welch Two Sample t-test
##
## data:  value by group
## t = -2.7683, df = 7.8542, p-value = 0.0248
## alternative hypothesis: true difference in means between group con and group LPS is not equal to 0
## 95 percent confidence interval:
##  -3.1176097 -0.2790193
## sample estimates:
## mean in group con mean in group LPS
##          1.000000          2.698314
```

```
## The result of t-test:
##  p = 0.025
##  A statistically significant difference exist between groups
```

Fig5_E.LPS post12days_qPCR_IL-10 Data analysis

Data structure

```
str(d1)
```

```
## 'data.frame':    11 obs. of  3 variables:
##  $ subject: int  1 2 3 4 5 6 7 8 9 10 ...
##  $ group  : chr  "con" "con" "con" "con" ...
##  $ IL.10  : num  0.0443 0.6266 1.4268 2.2333 0.6691 ...
```

Explorative data analysis with graphics

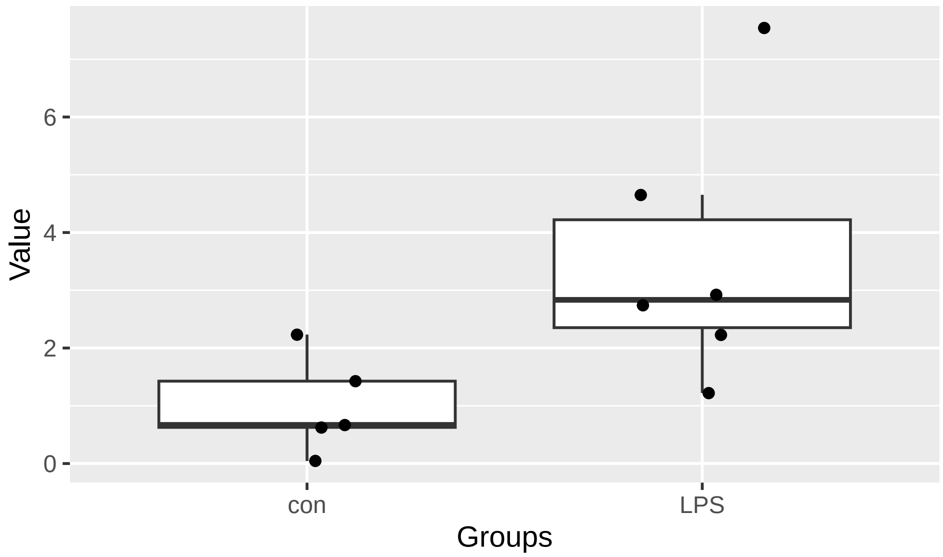


```
##
##  Welch Two Sample t-test
##
## data:  value by group
## t = -2.5659, df = 6.6022, p-value = 0.03914
## alternative hypothesis: true difference in means between group con and group LPS is not equal to 0
## 95 percent confidence interval:
##  -4.9301722 -0.1710328
## sample estimates:
## mean in group con mean in group LPS
##          1.000000          3.550603
```

```
## The result of t-test:
##  p = 0.039
##  A statistically significant difference exist between groups
```

Fig5_E.LPS post12days_qPCR_IL-6 Data analysis

Data structure

```
str(d1)
```

```
## 'data.frame':    12 obs. of  3 variables:
##  $ subject: int  1 2 3 4 5 6 7 8 9 10 ...
##  $ group  : chr  "con" "con" "con" "con" ...
##  $ IL.6   : num  0.0392 0.8073 1.1459 1.8392 1.1683 ...
```

Explorative data analysis with graphics

```
## Warning: Removed 1 rows containing non-finite values (`stat_boxplot()`).
```

```
## Warning: Removed 1 rows containing missing values (`geom_point()`).
```

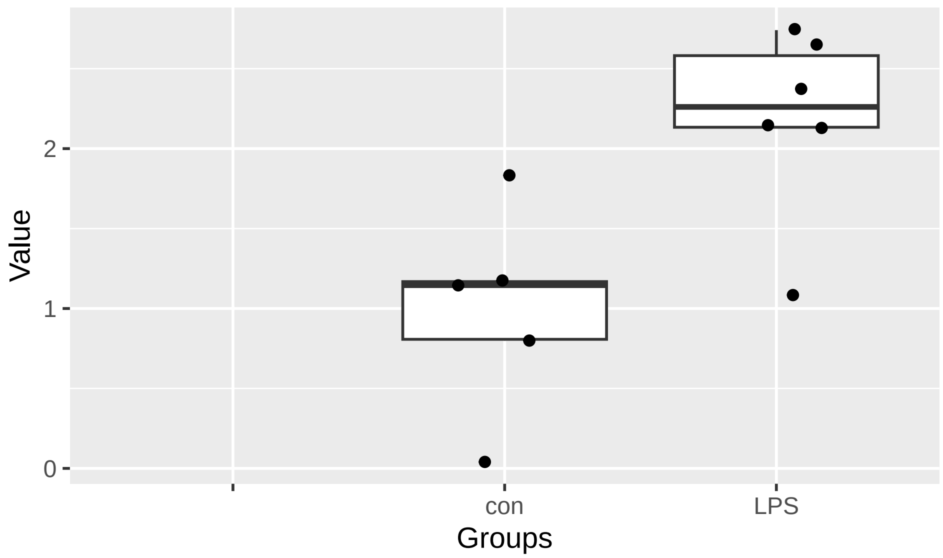


```
##
##  Welch Two Sample t-test
##
## data:  value by group
## t = -3.1209, df = 8.2748, p-value = 0.01363
## alternative hypothesis: true difference in means between group con and group LPS is not equal to 0
## 95 percent confidence interval:
```

```
##  -2.0606989 -0.3152477
## sample estimates:
## mean in group con mean in group LPS
##          1.000000          2.187974
```

```
## The result of t-test:
##  p = 0.014
##  A statistically significant difference exist between groups
```

SFigure3 Statistics

SFig3_A.Weight change_old mice Data analysis

Boohwi Hong

Package install

Data import

Data structure

```
str(d1)
```

```
## 'data.frame':    168 obs. of  4 variables:
##  $ subject: int  1 2 3 4 5 6 7 8 9 10 ...
##  $ group  : chr  "con" "con" "con" "con" ...
##  $ trial  : chr  "day1" "day1" "day1" "day1" ...
##  $ value  : num  58.8 37.7 36.2 50.6 41.4 46.4 34.7 56.2 37.1 61.5 ...
```

```
shapiro.test(d1$value)
```

```
##
##  Shapiro-Wilk normality test
##
## data:  d1$value
## W = 0.91534, p-value = 2.68e-08
```

Explorative data analysis with graphics

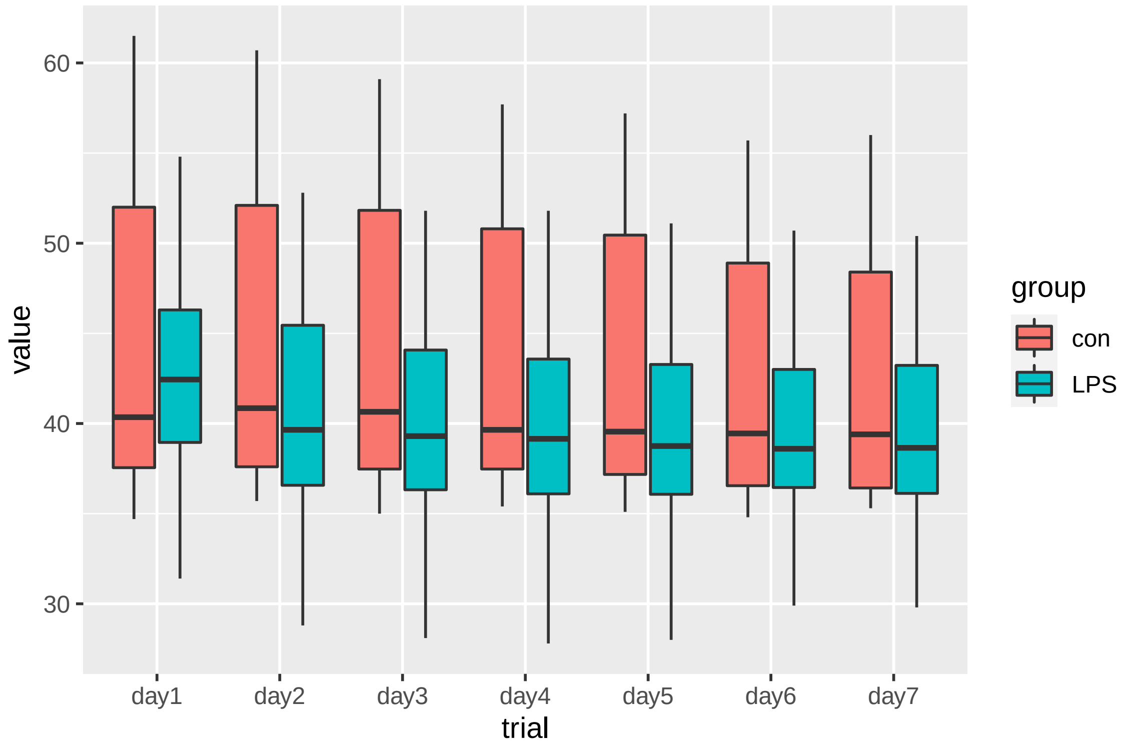

Model fit

```
##  F1 LD F1 Model
##  -----------------------
##  Check that the order of the time and group levels are correct.
##  Time level:   day1 day2 day3 day4 day5 day6 day7
##  Group level:   con LPS
##  If the order is not correct, specify the correct order in time.order or group.order.

##               Statistic df      p-value
## group         0.5648481  1 4.523134e-01
## trial        66.0676676  6 2.610687e-12
## group:trial 27.4589490  6 1.187700e-04

##               Statistic       df      p-value
## group         0.5648481 1.000000 4.523134e-01
## trial        19.7575777 3.150668 2.510144e-13
## group:trial   5.2076801 3.150668 1.099718e-03

##        Statistic df1      df2   p-value
## group 0.5648481   1 21.37913 0.4605076
```

Interpretation of result

```
## 1. Difference do not exist between groups
##  p = 0.452

## 2. Difference exist between measurement points
##  p = 0.000

## 3. Significant interaction exist between groups and measurement points
##  p = 0.001
```

SFigure4 Statistics

SFig4_B.Weight change_young mice

Data analysis using R

Boohwi Hong

Package install

Data import

Data structure

```
str(d1)
```

```
## 'data.frame':    147 obs. of  4 variables:
##  $ subject: int  1 2 3 4 5 6 7 8 9 10 ...
##  $ group  : chr  "con" "con" "con" "con" ...
##  $ trial  : int  1 1 1 1 1 1 1 1 1 1 ...
##  $ value  : num  22.3 22.6 22.5 22.1 22.2 20.9 20.8 22.2 23.7 20.6 ...
```

```
shapiro.test(d1$value)
```

```
##
##  Shapiro-Wilk normality test
##
## data:  d1$value
## W = 0.98605, p-value = 0.1453
```

Explorative data analysis with graphics

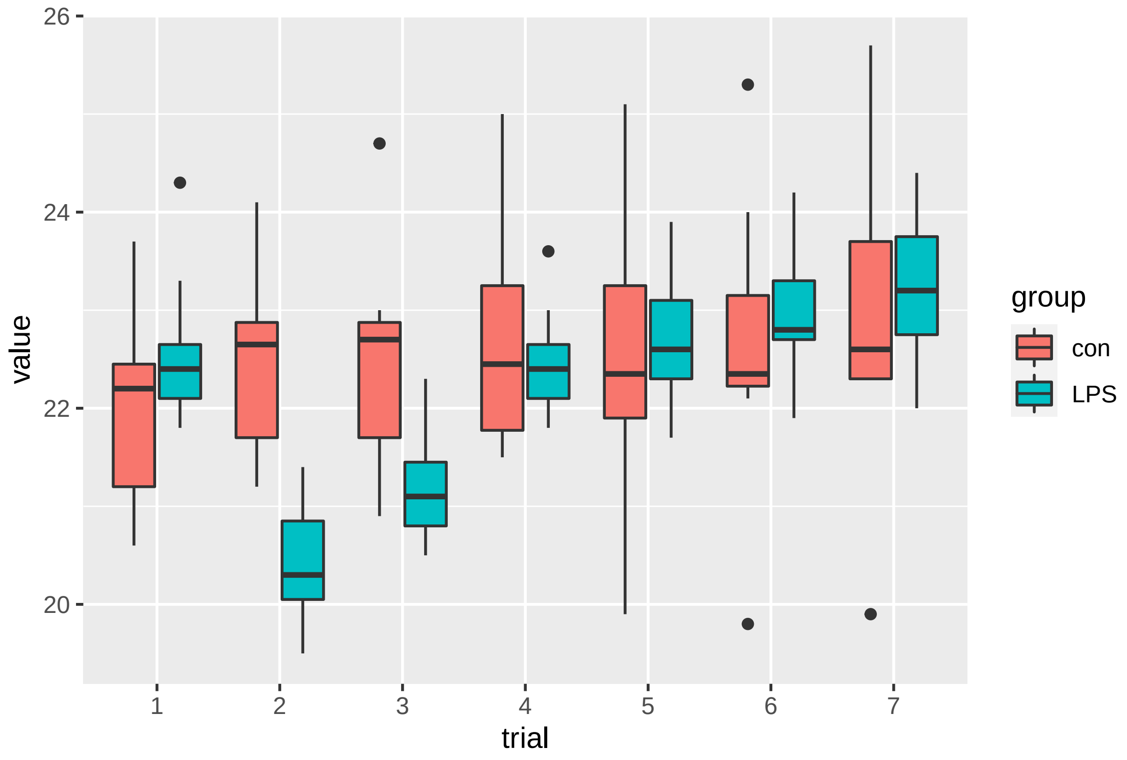

Model fit

```
##  F1 LD F1 Model
##  -----------------------
##  Check that the order of the time and group levels are correct.
##  Time level:   1 2 3 4 5 6 7
##  Group level:   con LPS
##  If the order is not correct, specify the correct order in time.order or group.order.

##                Statistic df      p-value
## group          0.2433831  1 6.217733e-01
## trial        243.7402435  6 8.920105e-50
## group:trial 166.6527361  6 2.305558e-33

##               Statistic       df      p-value
## group         0.2433831 1.000000 6.217733e-01
## trial        34.8836471 2.423213 1.069379e-18
## group:trial 25.5893804 2.423213 7.798253e-14

##       Statistic df1      df2   p-value
## group 0.2433831   1 13.62635 0.6296289
```

Interpretation of result

```
## 1. Difference do not exist between groups
##  p = 0.622

## 2. Difference exist between measurement points
##  p = 0.000

## 3. Significant interaction exist between groups and measurement points
##  p = 0.000
```

SFig4_C.Barnes maze_Primary Latency

Data analysis using R

Boohwi Hong

Package install

Data import

Data structure

```
str(d1)
```

```
## 'data.frame':    104 obs. of  4 variables:
##  $ subject: int  1 2 3 4 5 6 7 8 9 10 ...
##  $ group  : chr  "con" "con" "con" "con" ...
##  $ trial  : chr  "day1" "day1" "day1" "day1" ...
##  $ value  : num  109.6 54.2 86.8 22.5 12.5 ...
```

```
shapiro.test(d1$value)
```

```
##
##  Shapiro-Wilk normality test
##
## data:  d1$value
## W = 0.77465, p-value = 2.48e-11
```

Explorative data analysis with graphics

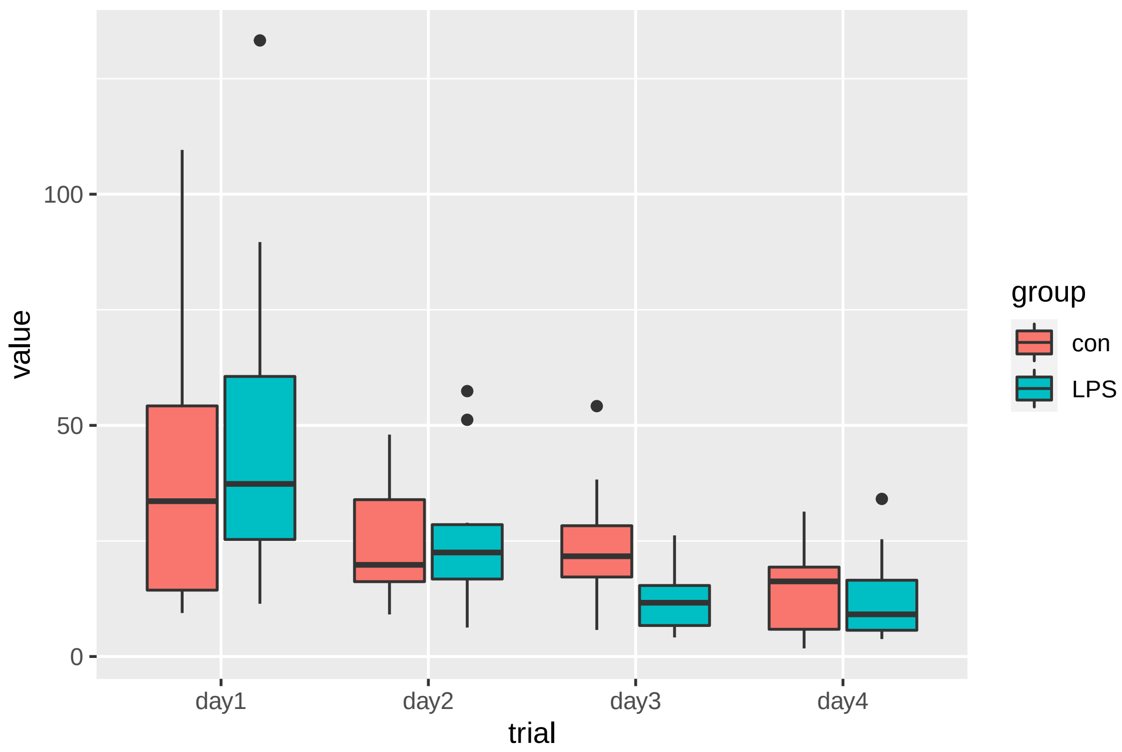

Model fit

```
##  F1 LD F1 Model
##  -----------------------
##  Check that the order of the time and group levels are correct.
##  Time level:   day1 day2 day3 day4
##  Group level:   con LPS
##  If the order is not correct, specify the correct order in time.order or group.order.

##              Statistic df      p-value
## group        0.9161287  1 3.384935e-01
## trial       25.9708385  3 9.672436e-06
## group:trial 10.9809187  3 1.182950e-02

##              Statistic       df      p-value
## group        0.9161287 1.000000 3.384935e-01
## trial       13.5174527 2.442487 1.396969e-07
## group:trial  2.6711862 2.442487 5.760378e-02

##       Statistic df1      df2   p-value
## group 0.9161287   1 19.72832 0.3500769
```

Interpretation of result

```
## 1. Difference do not exist between groups
##  p = 0.338

## 2. Difference exist between measurement points
##  p = 0.000

## 3. Significant interaction do not exist between groups and measurement points
##  p = 0.058
```

SFig4_C.Barnes maze_Primary Length

Data analysis using R

Boohwi Hong

Package install

Data import

Data structure

```
str(d1)
```

```
## 'data.frame':    104 obs. of  4 variables:
##  $ subject: int  1 2 3 4 5 6 7 8 9 10 ...
##  $ group  : chr  "con" "con" "con" "con" ...
##  $ trial  : chr  "day1" "day1" "day1" "day1" ...
##  $ value  : num  767 486 586 159 147 ...
```

```
shapiro.test(d1$value)
```

```
##
##  Shapiro-Wilk normality test
##
## data:  d1$value
## W = 0.84312, p-value = 4.035e-09
```

Explorative data analysis with graphics

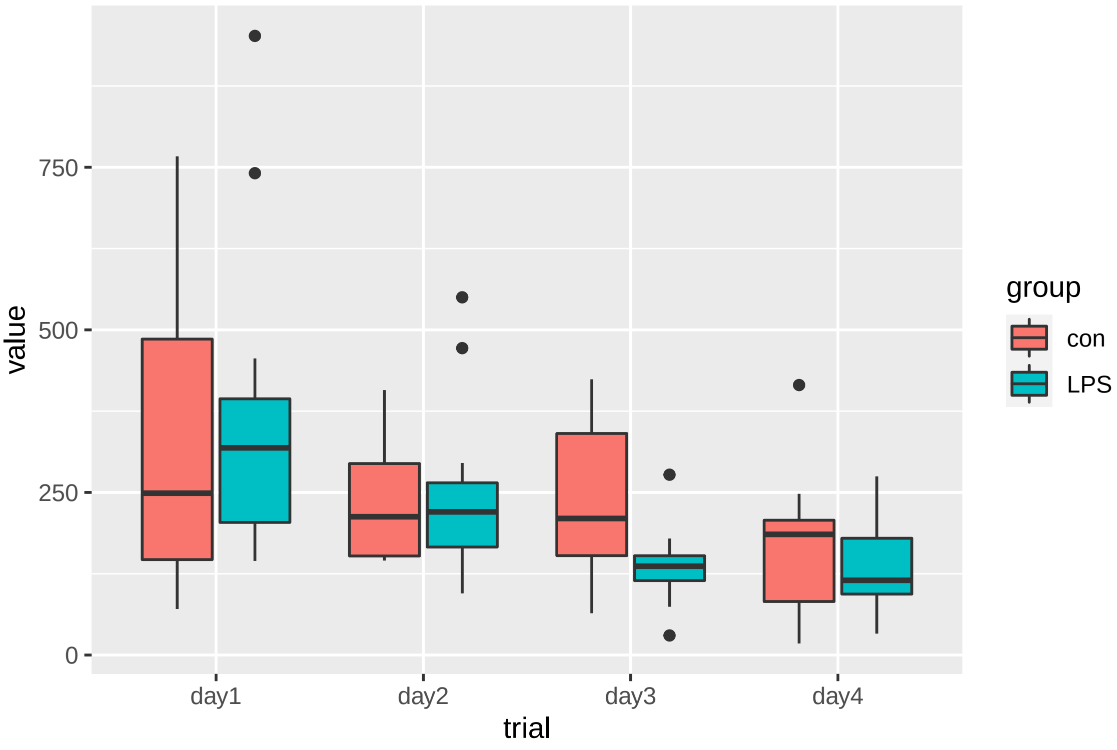

Model fit

```
##  F1 LD F1 Model
##  -----------------------
##  Check that the order of the time and group levels are correct.
##  Time level:   day1 day2 day3 day4
##  Group level:   con LPS
##  If the order is not correct, specify the correct order in time.order or group.order.

##              Statistic df      p-value
## group         1.46433  1 0.2262423495
## trial        16.96125  3 0.0007198289
## group:trial  11.59238  3 0.0089182821

##              Statistic       df      p-value
## group        1.464330 1.000000 2.262423e-01
## trial        8.791257 2.513427 3.329876e-05
## group:trial  3.251618 2.513427 2.809445e-02

##        Statistic df1      df2   p-value
## group   1.46433   1 19.36389 0.2408078
```

Interpretation of result

```
## 1. Difference do not exist between groups
##  p = 0.226

## 2. Difference exist between measurement points
##  p = 0.000

## 3. Significant interaction exist between groups and measurement points
##  p = 0.028
```

SFig4_C.Barnes maze_Primary errors

Data analysis using R

Boohwi Hong

Package install

Data import

Data structure

```
str(d1)
```

```
## 'data.frame':    104 obs. of  4 variables:
##  $ subject: int  1 2 3 4 5 6 7 8 9 10 ...
##  $ group  : chr  "con" "con" "con" "con" ...
##  $ trial  : chr  "day1" "day1" "day1" "day1" ...
##  $ value  : num  9.33 8 4.33 3 4.67 ...
```

```
shapiro.test(d1$value)
```

```
##
##  Shapiro-Wilk normality test
##
## data:  d1$value
## W = 0.94974, p-value = 0.0006048
```

Explorative data analysis with graphics

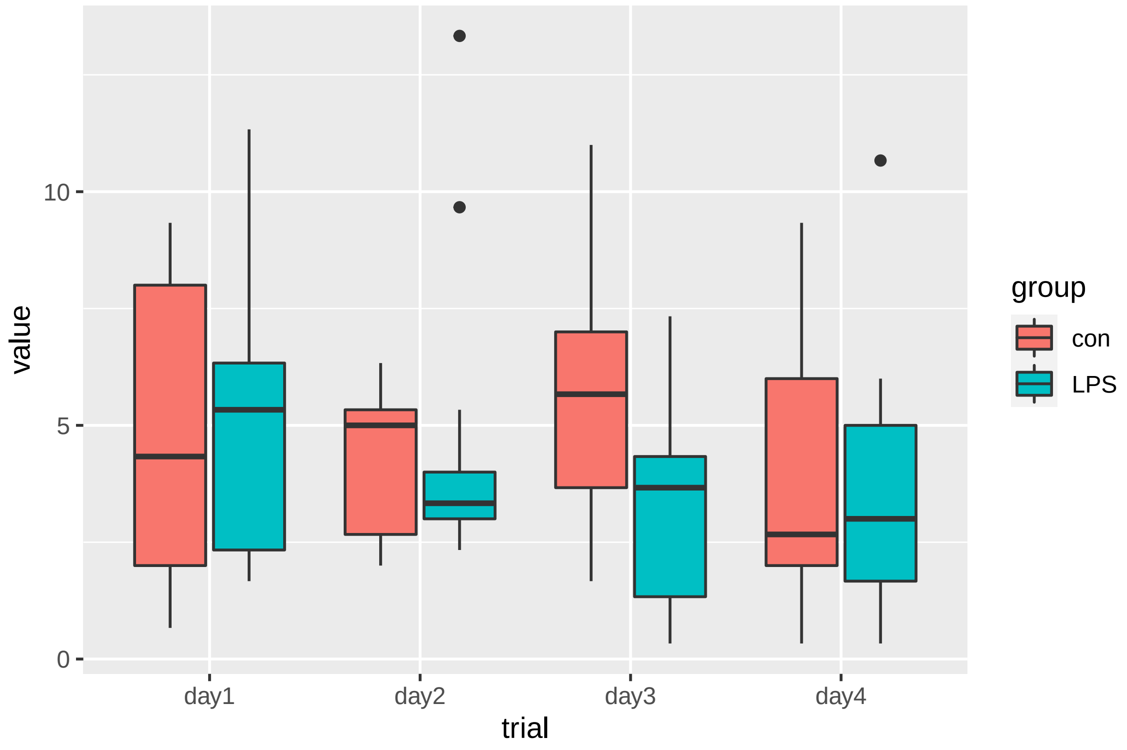

Model fit

```
##  F1 LD F1 Model
##  -----------------------
##  Check that the order of the time and group levels are correct.
##  Time level:   day1 day2 day3 day4
##  Group level:   con LPS
##  If the order is not correct, specify the correct order in time.order or group.order.

##              Statistic df   p-value
## group         1.705706  1 0.1915435
## trial         3.351822  3 0.3404949
## group:trial   7.509166  3 0.0573234

##              Statistic       df   p-value
## group         1.705706 1.000000 0.1915435
## trial         1.008550 2.318053 0.3736065
## group:trial   2.106059 2.318053 0.1134237

##        Statistic df1      df2   p-value
## group   1.705706   1 20.50936 0.2060027
```

Interpretation of result

```
## 1. Difference do not exist between groups
##  p = 0.192

## 2. Difference do not exist between measurement points
##  p = 0.374

## 3. Significant interaction do not exist between groups and measurement points
##  p = 0.113
```

SFig4_D.Barnes maze_Probe test_Duration Data analysis

Data structure

```
str(d1)
```

```
## 'data.frame':    26 obs. of  3 variables:
##  $ subject : int  1 2 3 4 5 6 7 8 9 10 ...
##  $ group   : chr  "con" "con" "con" "con" ...
##  $ Duration: num  21.1 42.2 44.3 52.3 55 ...
```

Explorative data analysis with graphics

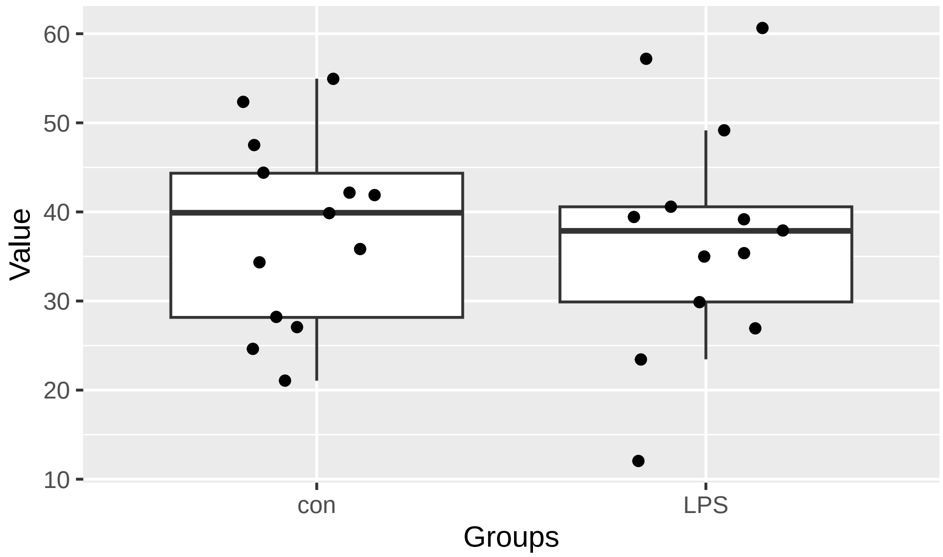


```
##
##  Welch Two Sample t-test
##
## data:  value by group
## t = 0.12109, df = 22.951, p-value = 0.9047
## alternative hypothesis: true difference in means between group con and group LPS is not equal to 0
## 95 percent confidence interval:
##  -9.165815 10.305399
## sample estimates:
## mean in group con mean in group LPS
##          38.01158          37.44178
```

```
## The result of t-test:
##  p = 0.905
##  A statistically significant difference do not exist between groups
```

SFig4_D.Barnes maze_Probe test_Distance Data analysis

Data structure

```
str(d1)
```

```
## 'data.frame':    26 obs. of  3 variables:
##  $ subject : int  1 2 3 4 5 6 7 8 9 10 ...
##  $ group   : chr  "con" "con" "con" "con" ...
##  $ Distance: num  2.844 2.188 0.227 0.518 0.828 ...
```

Explorative data analysis with graphics

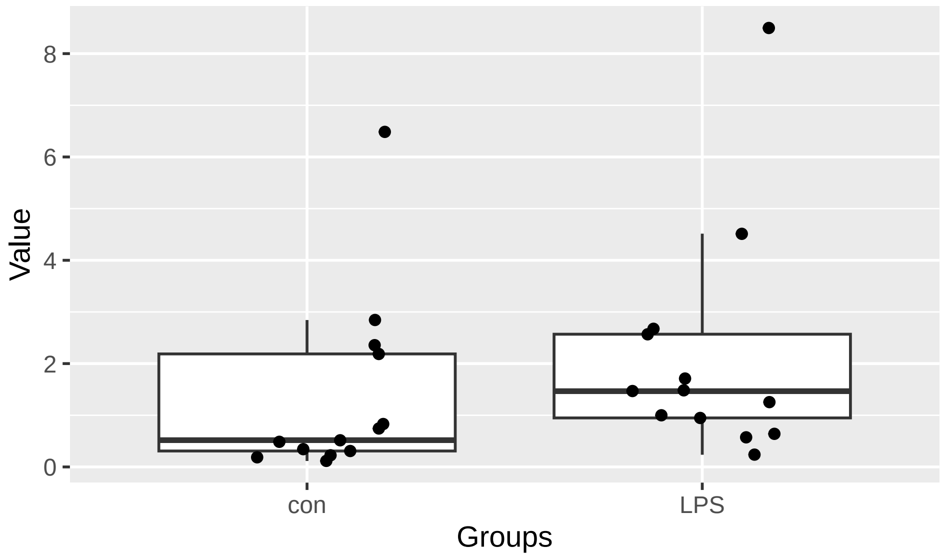


```
##
##  Welch Two Sample t-test
##
## data:  value by group
## t = -0.96519, df = 22.975, p-value = 0.3445
## alternative hypothesis: true difference in means between group con and group LPS is not equal to 0
## 95 percent confidence interval:
##  -2.404897  0.874766
## sample estimates:
## mean in group con mean in group LPS
##          1.356096          2.121161
```

```
## The result of t-test:
##  p = 0.344
##  A statistically significant difference do not exist between groups
```

SFig4_D.Barnes maze_Probe test_Latency Data analysis

Data structure

```
str(d1)
```

```
## 'data.frame':    26 obs. of  3 variables:
##  $ subject: int  1 2 3 4 5 6 7 8 9 10 ...
##  $ group  : chr  "con" "con" "con" "con" ...
##  $ Latency: num  33.8 15.31 1 3.6 4.57 ...
```

Explorative data analysis with graphics

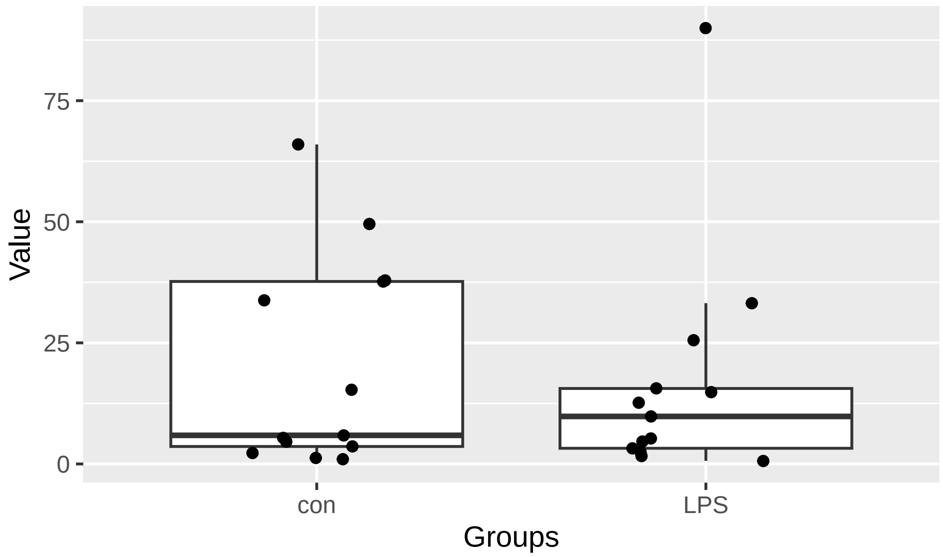


```
##
##  Welch Two Sample t-test
##
## data:  value by group
## t = 0.38116, df = 23.8, p-value = 0.7065
## alternative hypothesis: true difference in means between group con and group LPS is not equal to 0
## 95 percent confidence interval:
##  -15.19927  22.08109
## sample estimates:
## mean in group con mean in group LPS
##          20.31990          16.87898
```

```
## The result of t-test:
##  p = 0.706
##  A statistically significant difference do not exist between groups
```

SFig4_E.Fear chamber test Data analysis

Data structure

```
str(d1)
```

```
## 'data.frame':    26 obs. of  3 variables:
##  $ subject : int  1 2 3 4 5 6 7 8 9 10 ...
##  $ group   : chr  "con" "con" "con" "con" ...
##  $ Freezing: num  63.5 58.1 27 61.5 51.4 ...
```

Explorative data analysis with graphics

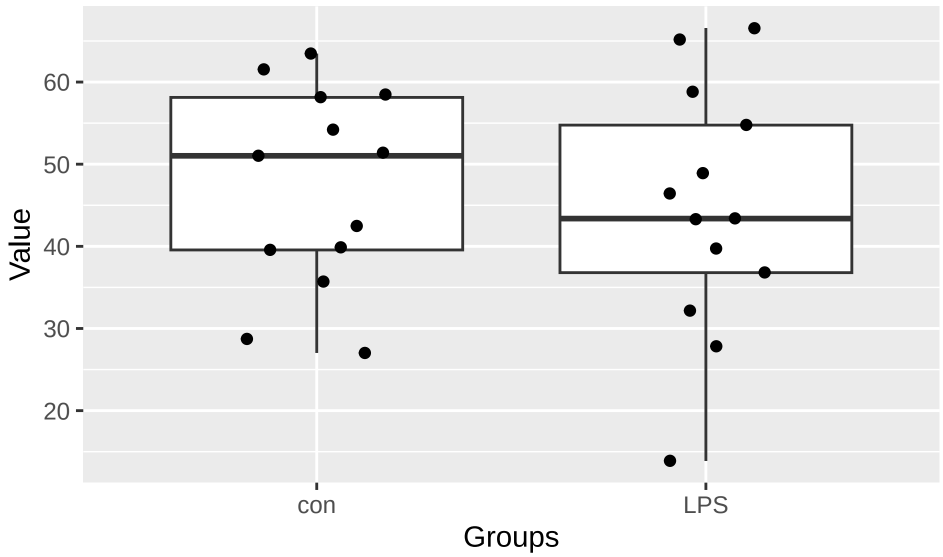


```
##
##  Welch Two Sample t-test
##
## data:  value by group
## t = 0.4854, df = 23.103, p-value = 0.632
## alternative hypothesis: true difference in means between group con and group LPS is not equal to 0
## 95 percent confidence interval:
##  -8.512873 13.734412
## sample estimates:
## mean in group con mean in group LPS
##          47.04923          44.43846
```

```
## The result of t-test:
##  p = 0.632
##  A statistically significant difference do not exist between groups
```
